# Mild and efficient synthesis and base-promoted rearrangement of novel isoxazolo[4,5-*b*]pyridines

**DOI:** 10.3762/bjoc.20.94

**Published:** 2024-05-14

**Authors:** Vladislav V Nikol’skiy, Mikhail E Minyaev, Maxim A Bastrakov, Alexey M Starosotnikov

**Affiliations:** 1 N.D. Zelinsky Institute of Organic Chemistry RAS, Leninsky prosp. 47, 119991 Moscow, Russia

**Keywords:** aromatic nitro compounds, Boulton–Katritzky rearrangement, isoxazolo[4,5-*b*]pyridines, nucleophilic substitution, 1,2,3-triazoles

## Abstract

An efficient method for the synthesis of isoxazolo[4,5-*b*]pyridines has been developed on the basis of readily available 2-chloro-3-nitropyridines via the intramolecular nucleophilic substitution of the nitro group as a key step. The previously unknown base-promoted Boulton–Katritzky rearrangement of isoxazolo[4,5-*b*]pyridine-3-carbaldehyde arylhydrazones into 3-hydroxy-2-(2-aryl[1,2,3]triazol-4-yl)pyridines was observed.

## Introduction

**Figure 1:**
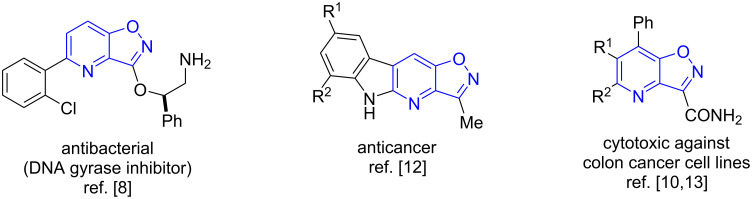
Some examples of biologically active isoxazolo[4,5-*b*]pyridines with antibacterial [8], anticancer [12] and cytotoxic [10,13] acitivities.

Nitrogen heterocycles represent a very important class of organic compounds that has found application in various fields of chemistry and materials science. These compounds are widespread in medicinal chemistry [1–3], production of high-energy-density compounds [4–7], and many others. In particular, isoxazolo[4,5-*b*]pyridines are of considerable interest due to their remarkable variety of biological activity, such as antibacterial [8], anticancer [9] or antiproliferative [10]. In addition, isoxazolo[4,5-*b*]pyridines were found to inhibit cytochrome P450 CYP17 responsible for the biosynthesis of androgens and estrogen precursors [11]. Some biologically active isoxazolo[4,5-*b*]pyridines are shown on Figure 1.

**Scheme 1:**
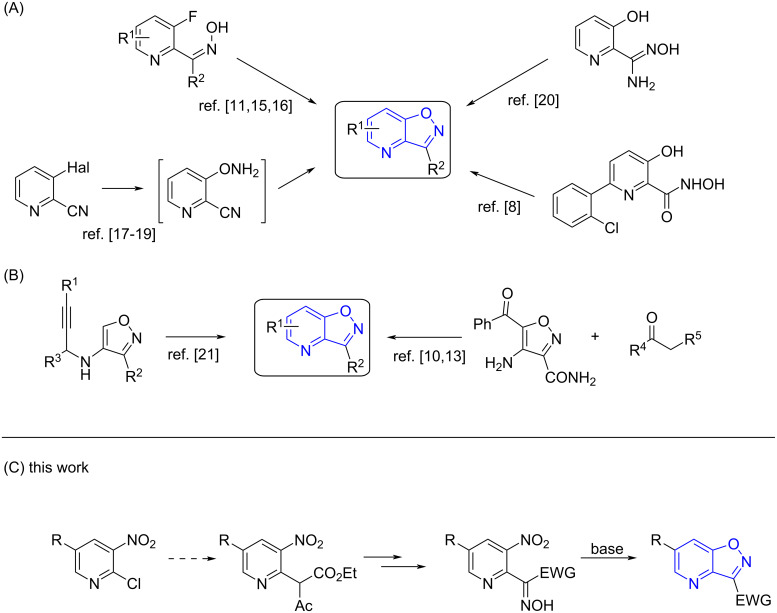
Methods for the synthesis of isoxazolo[4,5-*b*]pyridines: (A) annulation of an isoxazole fragment to a pyridine ring; (B) annulation of a pyridine ring to a functionalized isoxazole core; (C) synthesis from available 3-nitropyridines.

A number of isoxazolo[4,5-*b*]pyridines has been described in patents, however, there are only a few methods for their synthesis reported in the literature. First representatives of this heterocyclic system were described by Gewald et al. in 1980 [14]. The known methods are usually based on either annulation of an isoxazole fragment to a pyridine cycle or vice versa formation of a pyridine ring based on appropriately substituted isoxazoles. In the first case (Scheme 1A) 3-halo- [11,15–19] or 3-hydroxypyridines [8,20] bearing a suitable functionality in position 2 were used for the cyclization. Alternatively (Scheme 1B), isoxazolo[4,5-*b*]pyridines can be constructed via intramolecular cyclization of 4-(propargylamino)isoxazoles [21] or through reactions of 4-amino-5-benzoylisoxazoles with ketones or 1,3-dicarbonyl compounds [10,13]. These and some additional examples of isoxazolo[4,5-*b*]pyridine core synthesis have been summarized in a microreview [22].

Here, we wish to report an efficient method for the synthesis of isoxazolo[4,5-*b*]pyridines bearing electron-withdrawing groups (EWG) at positions 3 and 6 starting from readily available 2-chloro-3-nitro-6-R-pyridines as shown in Scheme 1C. Since the key step of the synthesis is the intramolecular nucleophilic substitution of the aromatic nitro group, we assumed that the presence of an electron-withdrawing substituent at the pyridine ring would facilitate this transformation.

## Results and Discussion

**Scheme 2:**
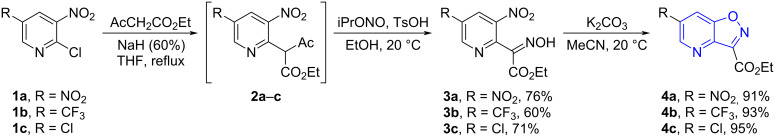
Synthesis of ethyl 6-R-isoxazolo[4,5-*b*]pyridine-3-carboxylates **4a**–**c**.

According to the general synthetic scheme (Scheme 1C), commercially available 2-chloro-3-nitropyridines **1a**–**c** were reacted with ethyl acetoacetate in the presence of NaH to give compounds **2a**–**c** which were not isolated and directly subjected to an in situ nitrosation affording isonitroso compounds **3a**–**c** in good yields. Cyclization of the latter under the action of K_2_CO_3_ in MeCN at room temperature gave previously unknown ethyl isoxazolo[4,5-*b*]pyridine-3-carboxylates **4a**–**c** (Scheme 2).

To the best of our knowledge only one compound (ethyl 5,7-dimethylisoxazolo[4,5-*b*]pyridine-3-carboxylate) has been synthesized using a similar method, however, the cyclization occurred under drastic conditions (NaH, DMF, 130 °C) as it was reported in patent literature [23].

**Scheme 3:**
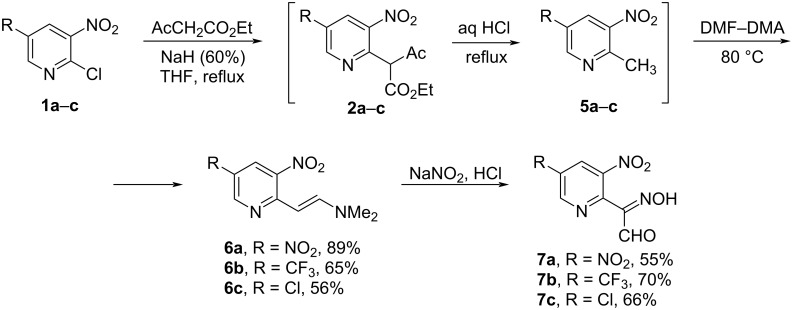
Synthesis of isonitroso compounds **7**.

We assumed that a similar synthetic route (nitrosation/S_N_Ar) would be applicable for the synthesis of isoxazolo[4,5-*b*]pyridine derivatives bearing other EWG in position 3, for example a formyl group. Thus, the key isonitroso compounds **7** were synthesized from chlorides **1a**–**c** via in situ formation of pyridylacetoacetic esters **2a**–**c** followed by decarbonylation to give 2-methyl-3-nitropyridines **5a**–**c** [24] which were used in the next step without purification. Their reactions with DMF-DMA afforded enamines **6** which, upon nitrosation, were converted into oximes **7a**–**c** in moderate yields (Scheme 3).

**Scheme 4:**
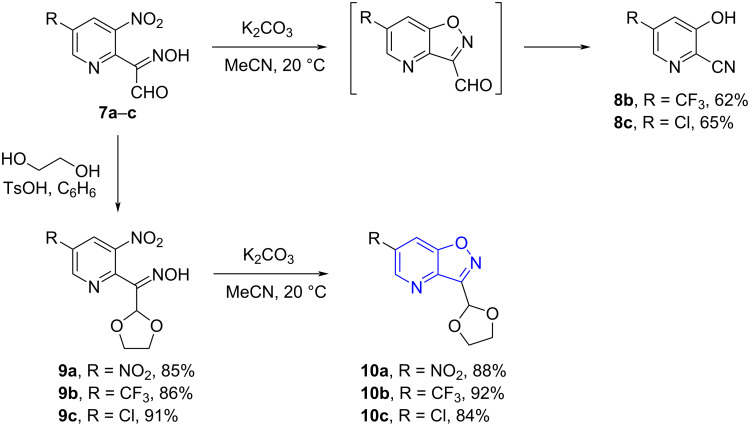
Base-promoted cyclization of compounds **7a**–**c**.

When compounds **7** were treated with K_2_CO_3_ 3-hydroxypyridine-2-carbonitriles **8** were obtained as sole products (Scheme 4). Apparently, a cyclization of oximes **7** to 3-formylisoxazolo[4,5-*b*]pyridine took place, followed by a base-promoted decarbonylation/isoxazole ring opening. Such transformations have been previously reported for benzo[*d*]isoxazoles with a carbonyl or carboxyl group in position 3 or 3-unsubstituted benzo[*d*]isoxazoles [25–29]. This means that the formyl group of compounds **7** should be protected prior to the attempted isoxazole ring formation. Indeed, reactions of **7a**–**c** with ethylene glycol gave dioxolane derivatives **9a**–**c** which were converted into isoxazolo[4,5-*b*]pyridines **10a**–**c** in high yields under mild conditions (Scheme 4).

**Scheme 5:**
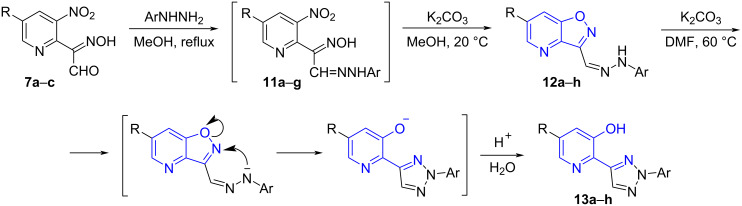
Synthesis and rearrangement of arylhydrazones **12**.

The obtained result prompted us to try another protecting group for the formyl function, namely arylhydrazone. Thus, reactions of compounds **7a**–**c** with a number of arylhydrazines afforded the corresponding hydrazones **11** which were not isolated and subjected to cyclization under the action of K_2_CO_3_ (Scheme 5). In most cases the isoxazolo[4,5-*b*]pyridines **12** were obtained in pure form, however, cyclization of hydrazone **11a** provided an inseparable mixture of two compounds which could be attributed to the target isoxazolo[4,5-*b*]pyridine **12a** and triazole **13a** formed as a result of Boulton–Katritzky rearrangement (Scheme 5). When this mixture was treated with K_2_CO_3_ in DMF at 60 °C, compound **13a** was isolated in 92% yield (from **7a**) (Table 1, entry 1). Such rearrangement has been reported previously for the benzo[*d*]isoxazole series [30], however, it has not been observed for isoxazolo[4,5-*b*]pyridine derivatives. It was found that the similar rearrangement of the other arylhydrazones **12b**–**h** strongly depends on the aryl substituent. Indeed, the 2,4-dinitrophenylhydrazones **12b**,**e**, and **h** did not undergo recyclization even under drastic conditions, apparently due to a low nucleophilicity of the hydrazone anion (Table 1, entries 2, 5, and 8). All other compounds **12** bearing no electron-withdrawing groups in the aryl moiety readily afforded the corresponding triazole derivatives in high yields under relatively mild conditions (K_2_CO_3_, DMF, 60 °C, Scheme 5). Substituents in the pyridine ring did not affect this transformation thus indicating that they do not participate in the stabilization of the pyridine-3-olate anion.

**Table 1:** Yields of compounds **12** and **13**.

Entry	R	Ar	Compound **12**, yield (%)	Compound **13**, yield (%)

1	NO_2_	C_6_H_5_	**12a**, not isolated	**13a**, 92
2	NO_2_	2,4-(NO_2_)_2_C_6_H_3_	**12b**, 87	**13b**, n.r.^a^
3	CF_3_	C_6_H_5_	**12c**, 85	**13c**, 95
4	CF_3_	2-Cl-C_6_H_4_	**12d**, 82	**13d**, 91
5	CF_3_	2,4-(NO_2_)_2_C_6_H_3_	**12e**, 71	**13e**, n.r.
6	Cl	C_6_H_5_	**12f**, 79	**13f**, 90
7	Cl	4-CH_3_-C_6_H_4_	**12g**, 76	**13g**, 95
8	Cl	2,4-(NO_2_)_2_C_6_H_3_	**12h**, 74	**13h**, n.r.

^a^No reaction.

**Figure 2:**
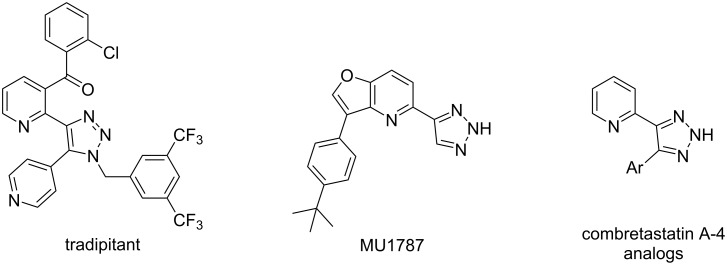
Biologically active analogs of compounds **13**.

It should be noted that the 4-(2-pyridyl)[1,2,3]triazole fragment is part of some pharmaceutically oriented molecules such as tradipitant, an experimental neurokinin-1 receptor antagonist [31], MU1787, a highly selective inhibitor of homeodomain-interacting protein kinases (HIPKs) [32], and combretastatin A-4 analogs evaluated for their anticancer properties against a panel of 60 human cancer cell lines [33] (Figure 2).

**Figure 3:**
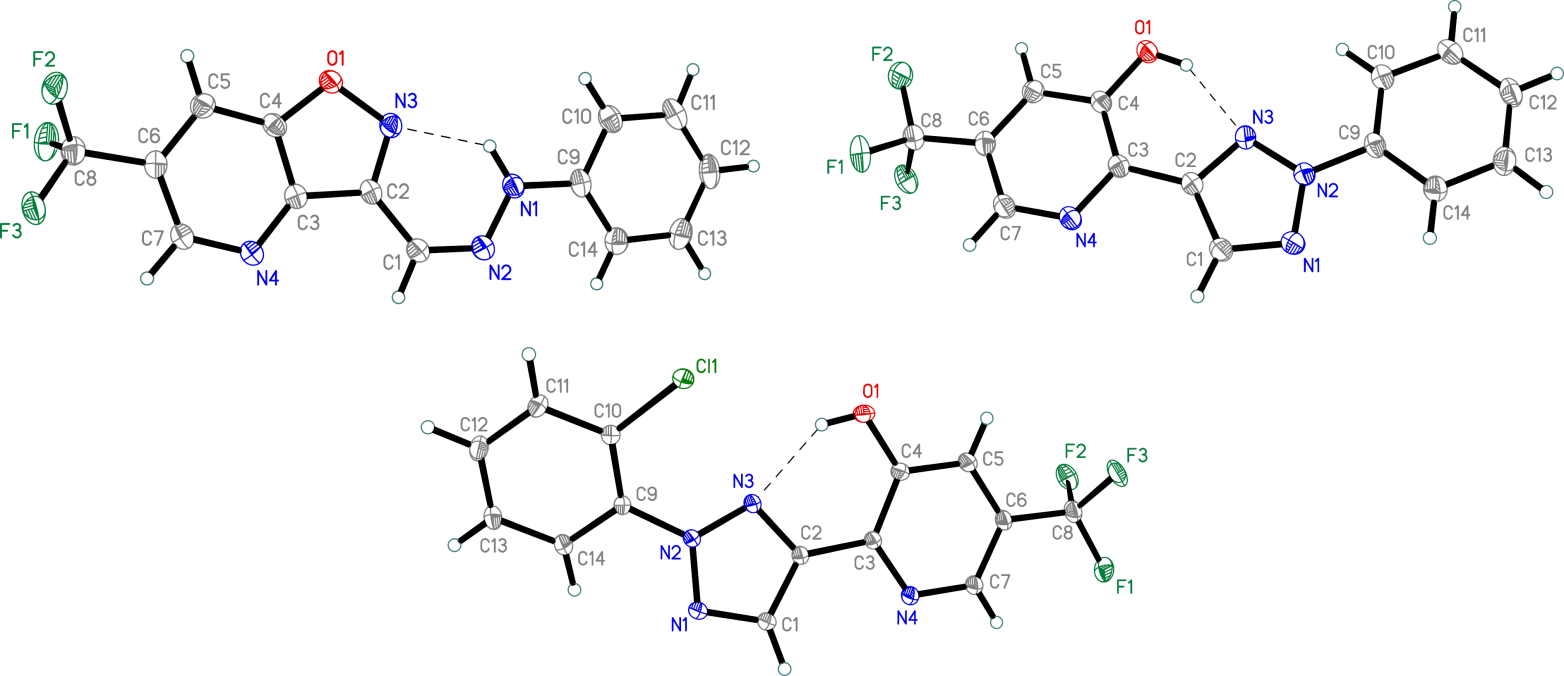
X-ray crystal structures of compounds **12c** (top left; the second crystallographically unique molecule is not shown), **13c** (top right), and **13d** (bottom) with thermal ellipsoids set at a 50% probability level. Intermolecular hydrogen bonds are drawn with dashed lines.

The structures of all new compounds were confirmed by ^1^H and ^13^C NMR and HRMS. X-ray diffraction studies were performed for compounds **12c** and **13c**,**d** (Figure 3; see Supporting Information File 1 for details) that allowed us to unambiguously establish the structures of both the starting hydrazones and rearrangement products.

## Conclusion

In summary, we have developed an efficient method for the synthesis of isoxazolo[4,5-*b*]pyridines based on the intramolecular nucleophilic substitution of the nitro group. The method comprises readily available starting materials, mild reaction conditions, easy work-up and high product yields. It was found that isoxazolo[4,5-*b*]pyridine-3-carbaldehyde arylhydrazones readily undergo a base-promoted Boulton–Katritzky rearrangement to give otherwise inaccessible 3-hydroxy-2-(2-aryl[1,2,3]triazol-4-yl)pyridines in excellent yields. As a result, a wide range of polyfunctional pyridines was synthesized, which can be considered as prospective platform for the design of pharmacology-oriented heterocyclic systems.

## Supporting Information

File 1 Experimental section, NMR spectra and X-ray analysis data.

## Data Availability

All data that supports the findings of this study is available in the published article and/or the supporting information to this article.
