# Supplementary material for: Mild and efficient synthesis and base-promoted rearrangement of novel isoxazolo[4,5-b]pyridines
Source: Beilstein J Org Chem. 2024 May 14;20:1069–75. doi: 10.3762/bjoc.20.94 (PMC11106669; doi:10.3762/bjoc.20.94)
Supplement: File 1 — Experimental section, NMR spectra and X-ray analysis data. [file Beilstein_J_Org_Chem-20-1069-s001.pdf]

BEILSTEIN JOURNAL OF ORGANIC CHEMISTRY

Supporting Information

for

Mild and efficient synthesis and base-promoted rearrangement of novel isoxazolo[4,5-*b*]pyridines

Vladislav V. Nikol’skiy, Mikhail E. Minyaev, Maxim A. Bastrakov and Alexey M. Starosotnikov

Beilstein J. Org. Chem. **2024,** *20,* 1069–1075. doi:10.3762/bjoc.20.94

Experimental section, NMR spectra and X-ray analysis data

License and Terms: This is a supporting information file under the terms of the Creative Commons Attribution License (https://creativecommons.org/licenses/by/4.0). Please note that the reuse, redistribution and reproduction in particular requires that the author(s) and source are credited and that individual graphics may be subject to special legal provisions.
The license is subject to the *Beilstein Journal of Organic Chemistry* terms and conditions: (https://www.beilstein-journals.org/bjoc/terms)

Experimental

All chemicals were of commercial grade and used directly without purification. Melting points were measured on a Stuart SMP20 apparatus (Stuart (Bibby Scientific), Stone, UK). ^{1}H and ^{13}C NMR spectra were recorded on a Bruker AM-300 (at 300.13 and 75.13 MHz, respectively, Bruker Biospin, Ettlingen, Germany) or a Bruker Avance DRX 500 (at 500 and 125 MHz, respectively, Bruker Biospin, Germany) in DMSO-d_6 or $CDCl_3$. *J* values are given in Hz. HRMS spectra were recorded on a Bruker micrOTOF II mass spectrometer using ESI. All reactions were monitored by TLC analysis using Merck TLC Silica gel 60 F_{254} plates, which were visualized with UV light. Compounds **1a–c** were purchased from commercial suppliers.

Synthesis of compounds 3a–c (general procedure):

To a stirred suspension of NaH (60% in mineral oil, 0.32 g, 8 mmol) in anhydrous THF (15 mL) ethyl acetoacetate (0.52 g, 4 mmol) was added dropwise. After 15 minutes of stirring, an appropriate 2-chloro-3-nitropyridine **1** (4 mmol) was added portion-wise. The reaction mixture was refluxed for 1–6 h (monitored with TLC), poured into 1 M aqueous HCl (75 mL) and extracted with $CHCl_3$. The extract was evaporated under reduced pressure and the residue was dissolved in 15 mL of ethanol. Freshly prepared isopropyl nitrite was added (1.2 mL, 12 mmol) followed by $TsOH\cdot H_2O$ (0.76 g, 4 mmol). The reaction mixture was stirred at rt overnight, diluted with ethyl acetate, washed with brine, and evaporated under reduced pressure. The crude product was purified by flash-chromatography on SiO_2 with 10% ethyl acetate in $CHCl_3$ as an eluent.

Ethyl (3,5-dinitropyridin-2-yl)(hydroxyimino)acetate (3a)

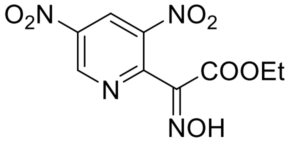

Yellow oil; yield 76%; ^{1}H NMR (300 MHz, $CDCl_3$): δ 1.26 (t, 3H, *J* = 7.2 Hz), 4.31 (q, 2H, *J* = 7.2 Hz), 9.21 (d, 1H, *J* = 1.8 Hz), 9.47 (br.s., 1H), 9.68 (d, 1H, *J* = 2.1 Hz); ^{13}C NMR (75 MHz, $CDCl_3$): δ 13.9, 63.2, 128.2, 143.8, 144.3, 145.8, 148.4, 149.9, 161.0. HRMS (ESI, m/z): calcd for $C_9H_8N_4O_7$ $[M + H]^+$: 285.0466; found: 285.0471.

Ethyl (hydroxyimino)[3-nitro-5-(trifluoromethyl)pyridin-2-yl]acetate (3b)

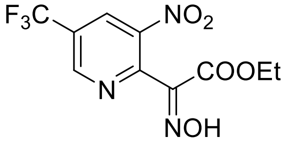

White crystals; yield 60%; mp 110-113 °C; ^{1}H NMR (300 MHz, $CDCl_3$): δ 1.33 (t, 3H, *J* = 7.1 Hz), 4.38 (q, 2H, *J* = 7.1 Hz), 8.79 (d, 1H, *J* = 1.6 Hz), 9.24 (d, 1H, *J* = 1.0 Hz), 9.77 (s, 1H). ^{13}C NMR (75 MHz, $CDCl_3$): δ 13.9, 63.0, 121.9 (q, $^{1}J_{CF}$ = 273.7 Hz), 128.2 (q, $^{2}J_{CF}$ = 35.5 Hz), 130.3 (d, $^{3}J_{CF}$ = 3.6 Hz), 144.2, 146.2, 148.2, 150.4 (d, $^{3}J_{CF}$ = 3.6 Hz), 161.1. HRMS (ESI, m/z): calcd for $C_{10}H_8F_3N_3NaO_5$ [M + Na]$^+$: 330.0308; found: 330.0310.

Ethyl (hydroxyimino)(5-chloro-3-nitropyridin-2-yl)acetate (3c)

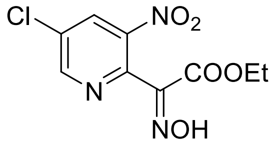

Colorless oil; yield 71%; ^{1}H NMR (300 MHz, $CDCl_3$): δ 1.33 (t, 3H, *J* = 7.1 Hz), 4.37 (q, 2H, *J* = 7.1 Hz), 8.55 (d, 1H, *J* = 2.0 Hz), 8.94 (d, 1H, *J* = 2.0 Hz), 9.70 (br.s, 1H). ^{13}C NMR (75 MHz, $CDCl_3$): δ 14.0, 62.7, 132.2, 133.2, 142.8, 144.4, 146.6, 152.9, 161.3. HRMS (ESI, m/z): calcd for $C_9H_8ClN_3O_5$ [M + H]$^+$: 274.0225; found: 274.0233.

Synthesis of compounds 6a–c (general procedure):

To a stirred suspension of NaH (60% in mineral oil, 3.20 g, 80 mmol) in anhydrous THF (160 mL) ethyl acetoacetate (5.20 g, 40 mmol) was added dropwise. After 15 minutes, an appropriate 2-chloro-3-nitropyridine (40 mmol) was added portion-wise. The reaction mixture was refluxed for 1–6 h (monitored with TLC), cooled to room temperature, and acidified with 200 mL of 15% hydrochloric acid. Most of the THF was removed under reduced pressure and the aqueous residue was refluxed for 1–2 h, cooled to rt, and extracted with $CHCl_3$. The combined extracts were evaporated under reduced pressure, the crude 2-methyl-3-nitropyridine **5** was dissolved in DMF (20 mL) and DMF-DMA (10 mL, 75 mmol) was added. The red solution was stirred at 80 °C for 1 h, cooled, and diluted with water. The precipitate was filtered off and recrystallized from isopropanol.

(*E*)-2-(3,5-Dinitropyridin-2-yl)-*N*,*N*-dimethylethylenamine (6a)

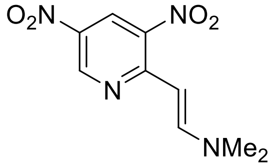

Red crystals; yield 89%; mp 195-196 °C; ^{1}H NMR (300 MHz, $CDCl_3$): δ 3.10 (s, 3H), 3.33 (s, 3H), 6.41 (d, 1H, *J* = 12.0 Hz), 8.44 (d, 1H, *J* = 12.0 Hz), 8.97 (s, 1H), 9.10 (s, 1H). ^{13}C NMR (125.76 MHz, DMSO-d_6): δ 37.6, 45.9, 92.2, 130.3, 134.8, 146.8, 155.1, 156.2. HRMS (ESI, m/z): calcd for $C_9H_{10}N_4O_4$ [M + H]$^+$: 239.0775; found: 239.0780.

(*E*)-*N*,*N*-Dimethyl-2-[3-nitro-5-(trifluoromethyl)pyridin-2-yl]ethylenamine (6b)

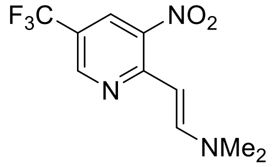

Red crystals; yield 65%; mp 89-90 °C; ^{1}H NMR (300 MHz, $CDCl_3$): δ 3.03 (br.s, 6H), 6.19 (d, 2H, J = 12.3 Hz), 8.18 (d, 2H, J = 12.3 Hz), 8.34 (s, 1H), 8.49 (s, 1H). ^{13}C NMR (75.47 MHz, $CDCl_3$): δ 37.3, 45.3, 91.4, 117.5 (q, $^{2}J_{CF}$ = 33.8 Hz), 123.3 (q, $^{1}J_{CF}$ = 269.5 Hz), 131.7 (q, $^{3}J_{CF}$ = 3.8 Hz), 136.1, 149.1 (q, $^{3}J_{CF}$ = 3.5 Hz), 153.1, 155.8. HRMS (ESI, m/z): calcd for $C_{10}H_{10}F_3N_3O_2$ [M + H]$^+$: 262.0798; found: 262.0804.

(*E*)-*N*,*N*-Dimethyl-2-(5-chloro-3-nitropyridin-2-yl)ethylenamine (6c)

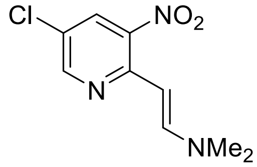

Red crystals; yield 56%; mp 124-125 °C; ^{1}H NMR (300 MHz, $CDCl_3$): δ 3.08 (br.s, 6H), 6.18 (d, 1H, J = 12.4 Hz), 8.07 (d, 1H, J = 12.4 Hz), 8.22 (s, 1H), 8.36 (s, 1H). ^{13}C NMR (75.47 MHz, $CDCl_3$): δ 25.4, 90.7, 121.8, 133.1, 137.4, 151.1, 151.6. HRMS (ESI, m/z): calcd for $C_9H_{10}ClN_3O_2$ [M + H]$^+$: 228.0534; found: 228.0537.

Synthesis of compounds 7a–c (general procedure):

A solution of $NaNO_2$ (3.80 g, 55 mmol) in H_2O (15 mL) was added dropwise to a solution of substituted enamine **6** (25 mmol) in conc. HCl (40 mL) with vigorous stirring. The temperature was kept near rt during the addition. The reaction mixture was stirred for additional 15 minutes, diluted with water, and extracted with $CHCl_3$. The combined extracts were washed several times with water, dried over anhydrous Na_2SO_4, and evaporated under reduced pressure. The crude product was purified by flash-chromatography on SiO_2 with 10% ethyl acetate in $CHCl_3$ as an eluent.

(3,5-Dinitropyridin-2-yl)(hydroxyimino)acetaldehyde (7a)

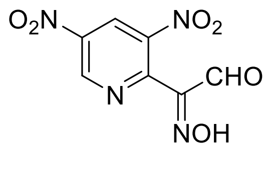

Yellow oil; yield 55%; ^{1}H NMR (300 MHz, DMSO-d_6): δ 9.21 (d, 1H, *J* = 2.0 Hz), 9.79 (d, 1H, *J* = 2.0 Hz), 9.85 (s, 1H), 13.96 (s, 1H). ^{13}C NMR (125.76 MHz, DMSO-d_6): δ 128.8, 144.1, 145.1, 147.3, 148.7, 153.1, 189.2.

(Hydroxyimino)[3-nitro-5-(trifluoromethyl)pyridin-2-yl]acetaldehyde (7b)

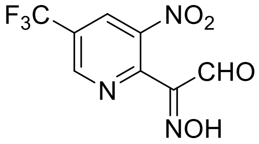

Yellow crystals; yield 70%; mp 105-108 °C; ^{1}H NMR (300 MHz, $CDCl_3$): δ 8.72 (s, 1H), 9.16 (s, 1H), 9.66 (s, 1H), 9.72 (s, 1H). ^{13}C NMR (125.76 MHz, $CDCl_3$): δ 121.7 (q, $^{1}J_{CF}$ = 273.8 Hz), 128.5 (q, $^{2}J_{CF}$ = 35.3 Hz), 130.4 (d, $^{3}J_{CF}$ = 3.4 Hz), 144.8, 146.8, 150.3 (d, $^{3}J_{CF}$ = 3.5 Hz), 154.0, 187.6. HRMS (ESI, m/z): calcd for $C_8H_4F_3N_3O_4$ $[M + H]^+$: 264.0227; found: 264.0224.

Hydroxyimino(5-chloro-3-nitropyridin-2-yl)acetaldehyde (7c)

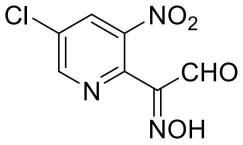

White solid; yield 66%; mp 179-180 °C with dec.; ^{1}H NMR (300 MHz, DMSO-d_6): δ 8.85 (s, 1H), 9.16 (s, 1H), 9.80 (s, 1H), 13.74 (s, 1H). ^{13}C NMR (75.47 MHz, DMSO-d_6): δ 132.2, 132.7, 141.1, 145.4, 153.0, 153.4, 189.4. HRMS (ESI, m/z): calcd for $C_7H_4ClN_3O_4$ $[M + H]^+$: 229.9963; found: 229.9967.

Synthesis of compounds 9a–c (general procedure):

An appropriate aldehyde (2mmol) was dissolved in benzene (30 mL) and ethylene glycol (0.5 mL) and a catalytic amount of $TsOH{\cdot}H_2O$ were added. The reaction mixture was refluxed for 2 h with a Dean–Stark adapter (monitored with TLC), washed with water, dried over anhydrous Na_2SO_4, and evaporated under reduced pressure.

(3,5-Dinitropyridin-2-yl)(1,3-dioxolan-2-yl)methanone oxime (9a)

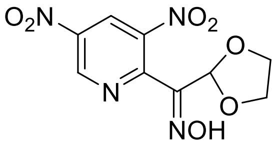

Off-white crystals; yield 85%; mp 152-153 °C; ^{1}H NMR (300 MHz, DMSO-d_6): δ 3.72-3.92 (m, 4H), 5.82 (s, 1H), 9.13 (d, 1H, *J* = 1.9 Hz), 9.73 (d, 1H, *J* = 1.9 Hz), 12.15 (s, 1H). ^{13}C NMR

(125.76 MHz, DMSO-d$_6$): δ 64.9, 101.3, 128.3, 143.6, 145.3, 147.9, 149.2, 149.4. HRMS (ESI, m/z): calcd for $C_9H_8N_4O_7$ [M + H]$^+$: 285.0466; found: 285.0465.

1,3-Dioxolan-2-yl[3-nitro-5-(trifluoromethyl)pyridin-2-yl]methanone oxime (9b)

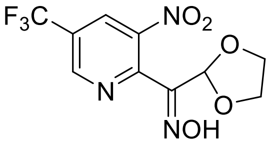

White solid; yield 86%; mp 82-83 °C; ^{1}H NMR (300 MHz, CDCl$_3$): δ 4.01 (s, 4H), 6.08 (s, 1H), 8.65 (s, 1H), 8.88 (s, 1H), 9.20 (s, 1H). ^{13}C NMR (75.47 MHz, CDCl$_3$): δ 65.3, 101.0, 122.0 (q, $^1J_{CF}$ = 271.7 Hz), 127.4 (q, $^2J_{CF}$ = 34.7 Hz), 129.7 (q, $^3J_{CF}$ = 3.5 Hz), 145.4, 148.7, 149.8 (q, $^3J_{CF}$ = 3.6 Hz), 150.6. HRMS (ESI, m/z): calcd for $C_{10}H_8F_3N_3O_5$ [M + H]$^+$: 308.0489; found: 308.0488.

(5-Chloro-3-nitropyridin-2-yl)(1,3-dioxolan-2-yl)methanone oxime (9c)

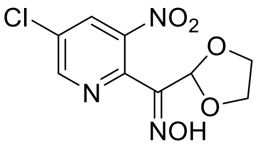

White crystals; yield 91%; mp 181-182 °C; ^{1}H NMR (300 MHz, DMSO-d$_6$): δ 3.72-3.88 (m, 4H), 5.77 (s, 1H), 8.74 (s, 1H), 9.08 (s, 1H), 11.92 (s, 1H). ^{13}C NMR (125.76 MHz, DMSO-d$_6$): δ 64.9, 101.3, 131.4, 132.3, 143.0, 145.8, 149.3, 152.2. HRMS (ESI, m/z): calcd for $C_9H_8ClN_3O_5$ [M + H]$^+$: 274.0225; found: 274.0235.

Synthesis of compounds 4a–c and 10a–c (general procedure):

To a solution of compound **3** or **9** (1 mmol) in dry MeCN (3 mL) was added finely powdered anhydrous K_2CO_3 (0.138 g, 1 mmol) and the mixture was stirred at rt for 1–12 h (monitored with TLC), then poured into water (15 mL), and acidified with conc. HCl to pH 3. The precipitate was filtered off, washed with water, and air-dried.

Ethyl 6-nitroisoxazolo[4,5-*b*]pyridine-3-carboxylate (4a)

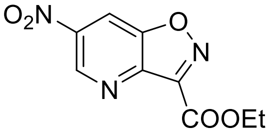

White crystals; yield 91%; mp 83-84 °C; ^{1}H NMR (300 MHz, CDCl$_3$): δ 1.45 (t, 3H, J = 7.0 Hz), 4.56 (q, 2H, J = 7.1 Hz), 8.77 (s, 1H), 9.63 (1H). ^{13}C NMR (75.47 MHz, CDCl$_3$): δ 14.2, 63.4,

114.2, 142.5, 143.8, 145.0, 151.1, 155.7, 157.8. HRMS (ESI, m/z): calcd for $C_9H_7N_3NaO_5$ [M + Na]$^+$: 260.0278; found: 260.0283.

Ethyl 6-(trifluoromethyl)isoxazolo[4,5-*b*]pyridine-3-carboxylate (4b)

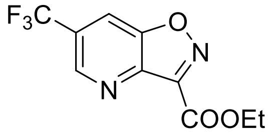

White crystals; yield 93%; mp 87-89 °C; ^{1}H NMR (300 MHz, $CDCl_3$): δ 1.45 (t, 3H, *J* = 7.0 Hz), 4.46 (q, 2H, *J* = 7.1 Hz), 8.24 (s, 1H), 9.08 (s, 1H). ^{13}C NMR (125.76 MHz, $CDCl_3$): δ 14.1, 63.2, 115.9 (q, $^3J_{CF}$ = 4.0 Hz), 122.8 (q, $^1J_{CF}$ = 273.7 Hz), 127.1 (q, $^2J_{CF}$ = 33.7 Hz), 141.1, 146.5 (d, $^3J_{CF}$ = 3.4 Hz), 150.9, 155.8, 158.2. HRMS (ESI, m/z): calcd for $C_{10}H_7F_3N_2NaO_3$ [M + Na]$^+$: 283.0301; found: 283.0302.

Ethyl 6-chloroisoxazolo[4,5-*b*]pyridine-3-carboxylate (4c)

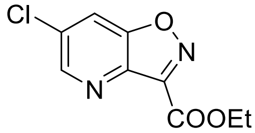

Off-white solid; yield 95%; mp 89-90 °C; ^{1}H NMR (300 MHz, $CDCl_3$): δ 1.51 (t, 3H, *J* = 7.1 Hz), 4.62 (q, 2H, *J* = 7.1 Hz), 8.05 (d, 1H, *J* = 1.4 Hz), 8.83 (d, 1H, *J* = 1.4 Hz). ^{13}C NMR (75.47 MHz, $CDCl_3$): δ 14.2, 63.0, 117.9, 133.5, 136.7, 149.4, 150.7, 157.1, 158.5. HRMS (ESI, m/z): calcd for $C_9H_7ClN_2O_3$ [M + Na]$^+$: 227.0218; found: 227.0215.

3-(1,3-Dioxolan-2-yl)-6-nitroisoxazolo[4,5-*b*]pyridine (10a)

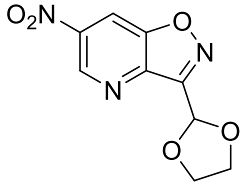

White crystals; yield 88%; mp 70-72 °C; ^{1}H NMR (300 MHz, DMSO-d_6): δ 4.11-4.36 (m, 4H), 6.55 (s, 1H), 9.31 (d, 1H, *J* = 1.7Hz), 9.60 (d, 1H, *J* = 1.7 Hz) . ^{13}C NMR (125.76 MHz, DMSO-d_6): δ 65.8, 96.3, 115.4, 141.6, 144.0, 144.4, 155.2, 157.5. HRMS (ESI, m/z): calcd for $C_9H_7N_3NaO_5$ [M + Na]$^+$: 260.0278; found: 260.0270.

3-(1,3-Dioxolan-2-yl)-6-(trifluoromethyl)isoxazolo[4,5-*b*]pyridine (10b)

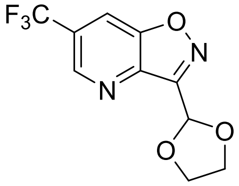

Amber oil; yield 92%; ^{1}H NMR (300 MHz, $CDCl_3$): δ 4.17-4.28 (m, 2H), 4.41-4.49 (m, 2H), 6.56 (s, 1H), 8.21 (s, 1H), 9.02 (s, 1H) . ^{13}C NMR (75.47 MHz, $CDCl_3$): δ 66.3, 97.2, 115.4 (q, $^{3}J_{CF}$ = 4.1 Hz), 123.1 (q, $^{1}J_{CF}$ = 271.7 Hz), 126.8 (q, $^{2}J_{CF}$ = 33.2 Hz), 141.6, 145.1 (q, $^{3}J_{CF}$ = 3.6 Hz), 155.1, 157.5. HRMS (ESI, m/z): calcd for $C_{10}H_7F_3N_2O_5$ $[M + H]^+$: 261.0482; found: 261.0484.

6-Chloro-3-(1,3-dioxolan-2-yl)isoxazolo[4,5-*b*]pyridine (10c)

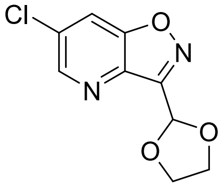

White solid; yield 84%; mp 75-76 °C; ^{1}H NMR (300 MHz, $CDCl_3$): δ 4.15-4.26 (m, 2H), 4.38-4.48 (m, 2H), 6.50 (s, 1H), 7.95 (s, 1H), 8.70 (s, 1H) . ^{13}C NMR (125.76 MHz, $CDCl_3$): δ 66.1, 97.2, 117.4, 133.0, 137.0, 147.8, 156.4, 157.1. HRMS (ESI, m/z): calcd for $C_9H_7ClN_2O_3$ $[M + H]^+$: 227.0218; found: 227.0223.

Synthesis of compounds 8b,c (general procedure):

A solution of compound **7** (1 mmol) in MeCN (3 mL) was stirred with powdered anhydrous K_2CO_3 (0.138 g, 1 mmol) at rt overnight, then poured into 1 M HCl (15 mL), and extracted with $CHCl_3$. The extract was dried over anhydrous Na_2SO_4 and evaporated under reduced pressure.

3-Hydroxy-5-(trifluoromethyl)pyridine-2-carbonitrile (8b)

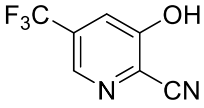

Off-white solid; yield 62%; mp 115-116 °C; ^{1}H NMR (300 MHz, DMSO-d_6): δ 7.69 (s, 1H), 8.55 (s, 1H), 12.50 (br.s, 1H). ^{13}C NMR (125.76 MHz, DMSO-d_6): δ 115.0, 121.4 (d, $^{3}J_{CF}$ = 3.7 Hz), 122.5 (q, $^{1}J_{CF}$ = 273.7 Hz), 124.3, 129.0 (q, $^{2}J_{CF}$ = 32.8 Hz), 137.7 (d, $^{3}J_{CF}$ = 3.6 Hz), 157.5. HRMS (ESI, m/z): calcd for $C_7H_3F_3N_2O$ $[M + H]^+$: 189.0270; found: 189.0270. IR (KBr): ν 768, 902, 951, 1084, 1141, 1169, 1243, 1267, 1328, 1355, 1456, 1607, 1694, 2246 (CN), 2563, 2612, 2786, 2890, 3017, 3088, 3115 cm^{-1}.

5-Chloro-3-hydroxypyridine-2-carbonitrile (8c)

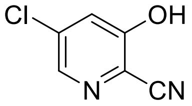

Off-white solid; yield 65%; mp 193-195 °C with dec.; ^{1}H NMR (300 MHz, DMSO-d$_6$): δ 7.53 (s, 1H), 8.26 (s, 1H), 12.31 (br.s., 1H). ^{13}C NMR (75.47 MHz, DMSO-d$_6$): δ 115.9, 119.6, 124.5, 135.8, 141.1, 158.5. MS (EI): m/z 154 [M]$^+$. IR (KBr): ν 607, 748, 885, 949, 1097, 1155, 1220, 1298, 1428, 1565, 1724, 2234 (CN), 2472, 2541, 2751, 2856, 2929, 2958, 3073, 3434 cm^{-1}.

Synthesis of compounds 12a–h (general procedure):

A solution of compound **7** (2 mmol) and arylhydrazine hydrochloride or 2,4-dinitrophenylhydrazine (2 mmol) in 10 mL of MeOH was stirred under reflux for 1–2 h (monitored with TLC) and cooled to rt. Powdered K_2CO_3 (0.552 g, 4 mmol (0.276 g, 2 mmol, in case of 2,4-dinitrophenylhydrazine)) was added and the reaction mixture was stirred overnight at rt, poured into water (50 mL), and acidified with conc. HCl to pH 3. The precipitate was filtered off and recrystallized from EtOH.

6-Nitroisoxazolo[4,5-*b*]pyridine-3-carbaldehyde phenylhydrazone (12a)

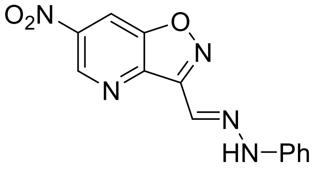

Not isolated. ^{1}H NMR (300 MHz, DMSO-d$_6$): δ 6.93 (t, 1H, J = 7.1 Hz), 7.24 (d, 2H, J = 7.9 Hz), 7.34 (t, 2H, J = 7.5 Hz), 8.21 (s, 1H), 9.23 (d, 1H, J = 1.8 Hz), 9.62 (d, 1H, J = 1.8 Hz), 11.32 (s, 1H).

6-Nitroisoxazolo[4,5-*b*]pyridine-3-carbaldehyde 2,4-dinitrophenylhydrazone (12b)

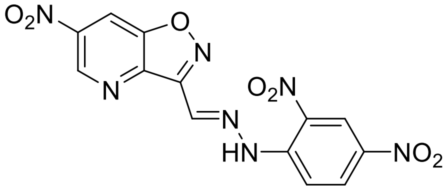

Brown solid; yield 87%; mp 251 °C with dec.; ^{1}H NMR (300 MHz, DMSO-d$_6$): δ 8.24 (d, 1H, J = 9.3 Hz), 8.55 (d, 1H, J = 9.3 Hz), 8.90 (s, 1H), 9.16 (s, 1H), 9.36 (s, 1H), 9.66 (s, 1H), 12.22 (br.s, 1H). HRMS (ESI, m/z): calcd for $C_{13}H_7N_7NaO_7$ [M + Na]$^+$: 396.0299; found: 396.0293.

6-Trifluoromethylisoxazolo[4,5-*b*]pyridine-3-carbaldehyde phenylhydrazone (12c)

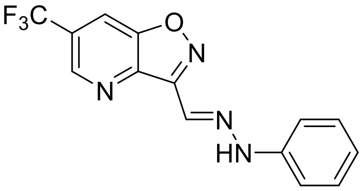

Yellow crystals; yield 85%; mp 205-206 °C; ^{1}H NMR (300 MHz, DMSO-d$_6$): δ 6.91 (t, 1H, *J* = 7.1 Hz), 7.24 (d, 2H, *J* = 7.9 Hz), 7.33 (t, 2H, *J* = 7.8 Hz), 8.21 (s, 1H), 8.88 (s, 1H), 9.25 (s, 1H), 11.28 (s, 1H). ^{13}C NMR (75.47 MHz, DMSO-d$_6$): δ 113.3, 117.0 (q, $^3J_{CF}$ = 4.0 Hz), 121.1, 122.9, 123.9 (q, $^1J_{CF}$ = 271.1 Hz), 125.7 (q, $^2J_{CF}$ = 32.4 Hz), 129.7, 142.5, 144.4, 145.5 (q, $^3J_{CF}$ = 3.5 Hz), 155.1, 155.2. HRMS (ESI, m/z): calcd for $C_{14}H_9F_3N_4O$ [M + H]$^+$: 307.0801; found: 307.0802.

6-Trifluoromethylisoxazolo[4,5-*b*]pyridine-3-carbaldehyde 2-chlorophenylhydrazone (12d)

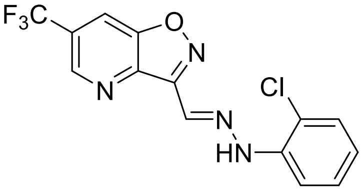

Yellow solid; yield 82%; mp 195-197 °C; ^{1}H NMR (300 MHz, DMSO-d$_6$): δ 7.60-7.69 (m, 3H), 7.79-7.83 (m, 2H), 8.56 (s, 1H), 8.68 (s, 1H), 11.41 (br.s, 1H). ^{13}C NMR (125.76 MHz, DMSO-d$_6$): δ 120.1 (d, $^3J_{CF}$ = 4.0 Hz), 123.4 (q, $^1J_{CF}$ = 272.9 Hz), 125.4 (d, $^2J_{CF}$ = 32.1 Hz), 128.3, 128.4, 128.6, 130.9, 131.5, 136.5 (d, $^3J_{CF}$ = 4.3 Hz), 136.9, 137.5, 140.1, 145.5, 151.6. HRMS (ESI, m/z): calcd for $C_{14}H_8ClF_3N_4O$ [M + H]$^+$: 341.0411; found: 341.0410.

6-Trifluoromethylisoxazolo[4,5-*b*]pyridine-3-carbaldehyde 2,4-dinitrophenylhydrazone (12e)

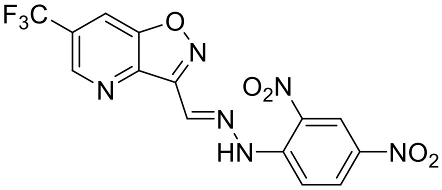

Yellow solid; yield 71%; mp 242-243 °C; ^{1}H NMR (300 MHz, DMSO-d$_6$): δ 8.17 (d, 1H, *J* = 9.5 Hz), 8.46 (d, 1H, *J* = 9.2 Hz), 8.82 (s, 1H), 8.99 (s, 1H), 9.10 (s, 1H), 9.24 (s, 1H), 12.15 (br.s, 1H). HRMS (ESI, m/z): calcd for $C_{14}H_7F_3N_6O_5$ [M - H]$^+$: 395.0357; found: 395.0350.

6-Chloroisoxazolo[4,5-*b*]pyridine-3-carbaldehyde phenylhydrazone (12f)

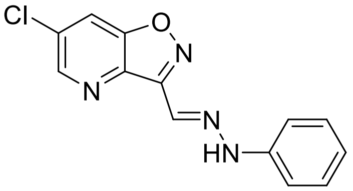

Yellow crystals; yield 79%; mp 214-215 °C; ^{1}H NMR (300 MHz, DMSO-d$_6$): δ 6.90 (t, 1H, *J* = 6.7 Hz), 7.0-7.35 (m, 4H), 8.16 (s, 1H), 8.60 (s, 1H), 8.90 (s, 1H), 11.24 (s, 1H). ^{13}C NMR (75.47 MHz, DMSO-d$_6$): δ 113.3, 118.6, 121.0, 123.2, 129.7, 132.5, 137.9, 144.5, 148.0, 154.9, 156.3. HRMS (ESI, m/z): calcd for $C_{13}H_9ClN_4O$ [M + H]$^+$: 273.0538; found: 273.0544.

6-Chloroisoxazolo[4,5-*b*]pyridine-3-carbaldehyde 4-methylphenylhydrazone (12g)

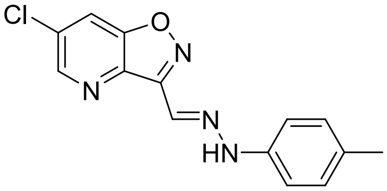

Orange crystals; yield 76%; mp 213-214 °C; ^{1}H NMR (300 MHz, DMSO-d$_6$): δ 2.26 (s, 3H), 7.12 (s, 4H), 8.12 (s, 1H), 8.59 (s, 1H), 8.89 (s, 1H), 11.15 (s, 1H). ^{13}C NMR (125.76 MHz, DMSO-d$_6$): δ 20.4, 112.8, 118.2, 121.9, 129.3, 129.7, 132.0, 137.5, 141.7, 147.6, 154.5, 155.8. HRMS (ESI, m/z): calcd for $C_{14}H_{11}ClN_4O$ [M + H]$^+$: 287.0694; found: 287.0699.

6-Chloroisoxazolo[4,5-*b*]pyridine-3-carbaldehyde 2,4-dinitrophenylhydrazone (12h)

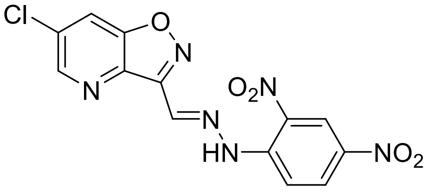

Yellow solid; yield 74%; mp 245-247 °C; ^{1}H NMR (300 MHz, DMSO-d$_6$): δ 8.21 (d, 1H, *J* = 9.6 Hz), 8.53 (dd, 1H, *J* = 9.5 Hz, *J* = 2.4 Hz), 8.72 (d, 1H, *J* = 1.8 Hz), 8.90 (d, 1H, *J* = 2.4 Hz), 8.95 (d, 1H, *J* = 2.1 Hz), 9.11 (s, 1H), 12.16 (br.s., 1H). HRMS (ESI, m/z): calcd for $C_{13}H_7ClN_6O_5$ [M + H]$^+$: 363.0239; found: 363.0239.

Synthesis of compounds 13a,c,d,f,g (general procedure):

To a solution of hydrazone **12** (1 mmol) in anhydrous DMF (3 mL) was added powdered anhydrous K_2CO_3 (0.138 g, 1 mmol) and the mixture was stirred for 1–3 h at 60 °C (monitored with TLC). Then, it was poured into water (15 mL) and acidified with conc. HCl to pH 3. The precipitate was filtered off, washed with water, and air-dried.

5-Nitro-2-(2-phenyl-2*H*-1,2,3-triazol-4-yl)pyridin-3-ol (13a)

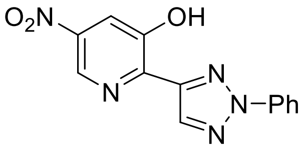

Beige solid; yield 92%; mp 253-255 °C; ^{1}H NMR (300 MHz, DMSO-d_6): δ 7.50 (t, 1H, *J* = 7.4 Hz), 7.63 (t, 2H, *J* = 7.5 Hz), 8.10-8.15 (m, 3H), 8.71 (s, 1H), 9.02 (d, 1H, *J* = 1.4 Hz), 11.76 (s, 1H). ^{13}C NMR (125.76 MHz, $CDCl_3$): δ 117.9, 118.8, 128.4, 129.9, 135.2, 137.6, 139.1, 141.6, 143.7, 145.1, 151.8. HRMS (ESI, m/z): calcd for $C_{13}H_9N_5O_3$ $[M + H]^+$: 274.0778; found: 274.0786.

5-Trifluoromethyl-2-(2-phenyl-2*H*-1,2,3-triazol-4-yl)pyridin-3-ol (13c)

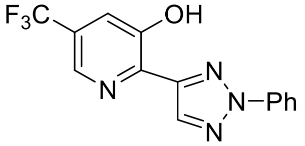

Beige solid; yield 95%; mp 140-141 °C; ^{1}H NMR (300 MHz, $CDCl_3$): δ 7.47 (t, 1H, *J* = 7.4 Hz), 7.58 (t, 2H, *J* = 7.5 Hz), 7.66 (s, 1H), 8.10 (d, 2H, *J* = 7.9 Hz), 8.54 (s, 1H), 8.57 (s, 1H), 9.76 (s, 1H). ^{13}C NMR (125.76 MHz, $CDCl_3$): δ 118.9, 121.6 (d, $^{3}J_{CF}$ = 3.5 Hz), 123.1 (q, $^{1}J_{CF}$ = 273.0 Hz), 127.3 (q, $^{2}J_{CF}$ = 33.1 Hz), 128.4, 129.6, 134.9, 136.9, 137.7 (d, $^{3}J_{CF}$ = 4.0 Hz), 138.8, 147.8, 151.3. HRMS (ESI, m/z): calcd for $C_{14}H_9F_3N_4O$ $[M + H]^+$: 307.0801; found: 307.0797.

5-Trifluoromethyl-2-(2-(2-chlorophenyl)-2*H*-1,2,3-triazol-4-yl)pyridin-3-ol (13d)

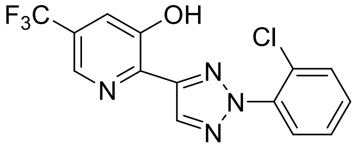

Beige solid; yield 91%; mp 152-154 °C; ^{1}H NMR (300 MHz, $CDCl_3$): δ 7.49-7.52 (m, 2H), 7.64-7.68 (m, 2H), 7.75 -7.78 (m, 1H), 8.54 (s, 1H), 8.62 (s, 1H), 9.74 (s, 1H). ^{13}C NMR (125.76 MHz, $CDCl_3$): δ 121.9, 123.0 (q, $^{1}J_{CF}$ = 272.8 Hz), 127.1, 127.5 (q, $^{2}J_{CF}$ = 33.1 Hz), 127.7, 128.8, 130.7, 131.4, 135.1, 136.6, 136.9, 137.3 (d, $^{3}J_{CF}$ = 3.7 Hz), 147.5, 151.7. HRMS (ESI, m/z): calcd for $C_{14}H_8ClF_3N_4O$ $[M + H]^+$: 341.0411; found: 341.0415.

5-Chloro-2-(2-phenyl-2*H*-1,2,3-triazol-4-yl)pyridin-3-ol (13f)

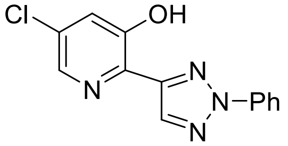

Beige solid; yield 90%; mp 153-154 °C; ^{1}H NMR (300 MHz, DMSO-d$_6$): δ 7.44-7.52 (m, 2H,), 7.61 (t, 2H, *J* = 7.5 Hz), 8.11 (d, 2H, *J* = 7.8 Hz), 8.27 (s, 1H), 8.55 (s, 1H), 11.16 (s, 1H). ^{13}C NMR (150.90 MHz, DMSO-d$_6$): δ 118.6, 123.1, 128.0, 129.8, 130.6, 134.9, 136.5, 138.8, 139.1, 145.8, 152.3. HRMS (ESI, m/z): calcd for $C_{13}H_9ClN_4O$ [M + H]$^+$: 273.0538; found: 273.0550.

5-Chloro-2-(2-(4-methylphenyl)-2*H*-1,2,3-triazol-4-yl)pyridin-3-ol (13g)

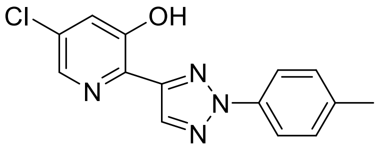

Beige solid; yield 95%; mp 171-172 °C; ^{1}H NMR (300 MHz, DMSO-d$_6$): δ 2.39 (s, 3H), 7.40 (d, 2H, *J* = 7.9 Hz), 7.49 (s, 1H), 7.99 (d, 2H, *J* = 7.9 Hz), 8.26 (s, 1H), 8.52 (s, 1H), 11.10 (br.s, 1H). ^{13}C NMR (150.90 MHz, DMSO-d$_6$): δ 20.6, 118.5, 123.1, 130.2, 130.5, 135.0, 136.2, 137.1, 137.5, 138.8, 145.6, 152.2. HRMS (ESI, m/z): calcd for $C_{14}H_{11}ClN_4O$ [M + H]$^+$: 287.0694; found: 287.0700.

NMR spectra of synthesized compounds

^{1}H and ^{13}C NMR spectra of compound **3a** in $CDCl_3$

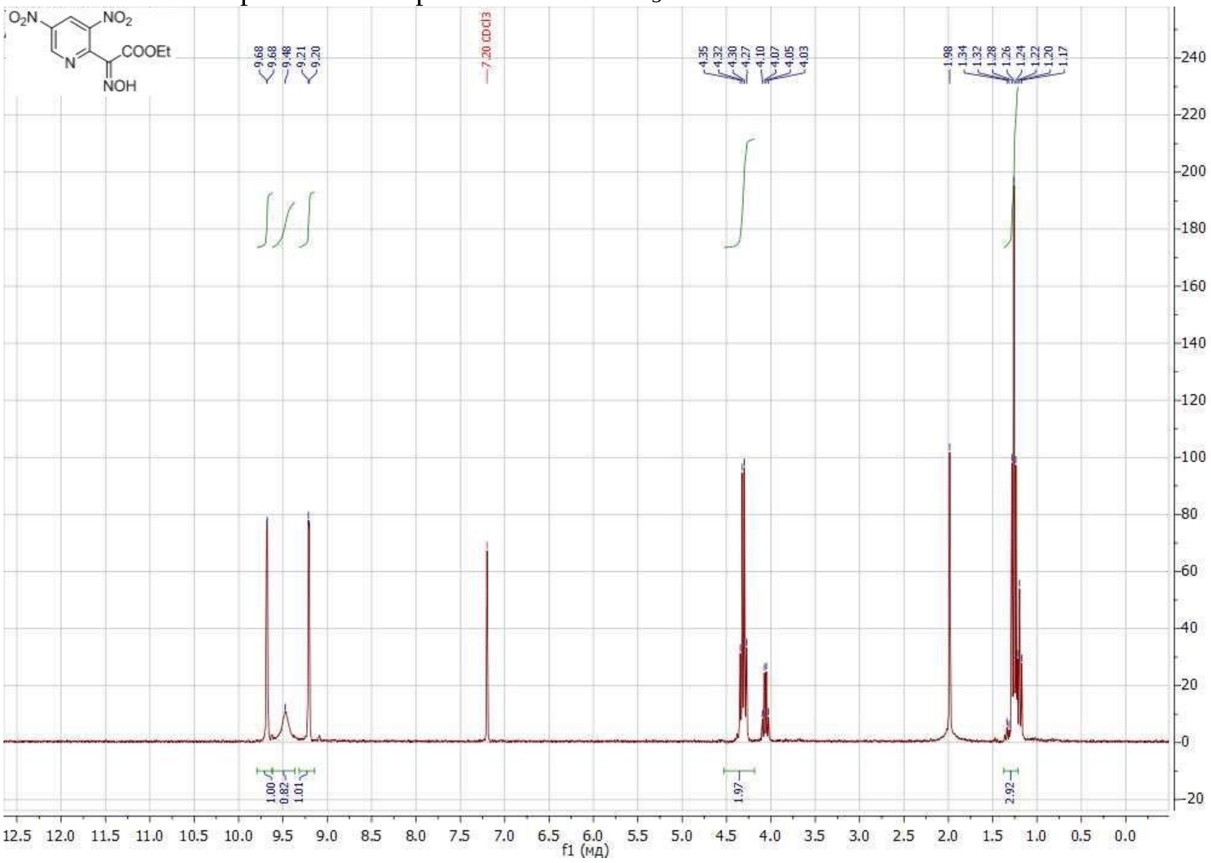

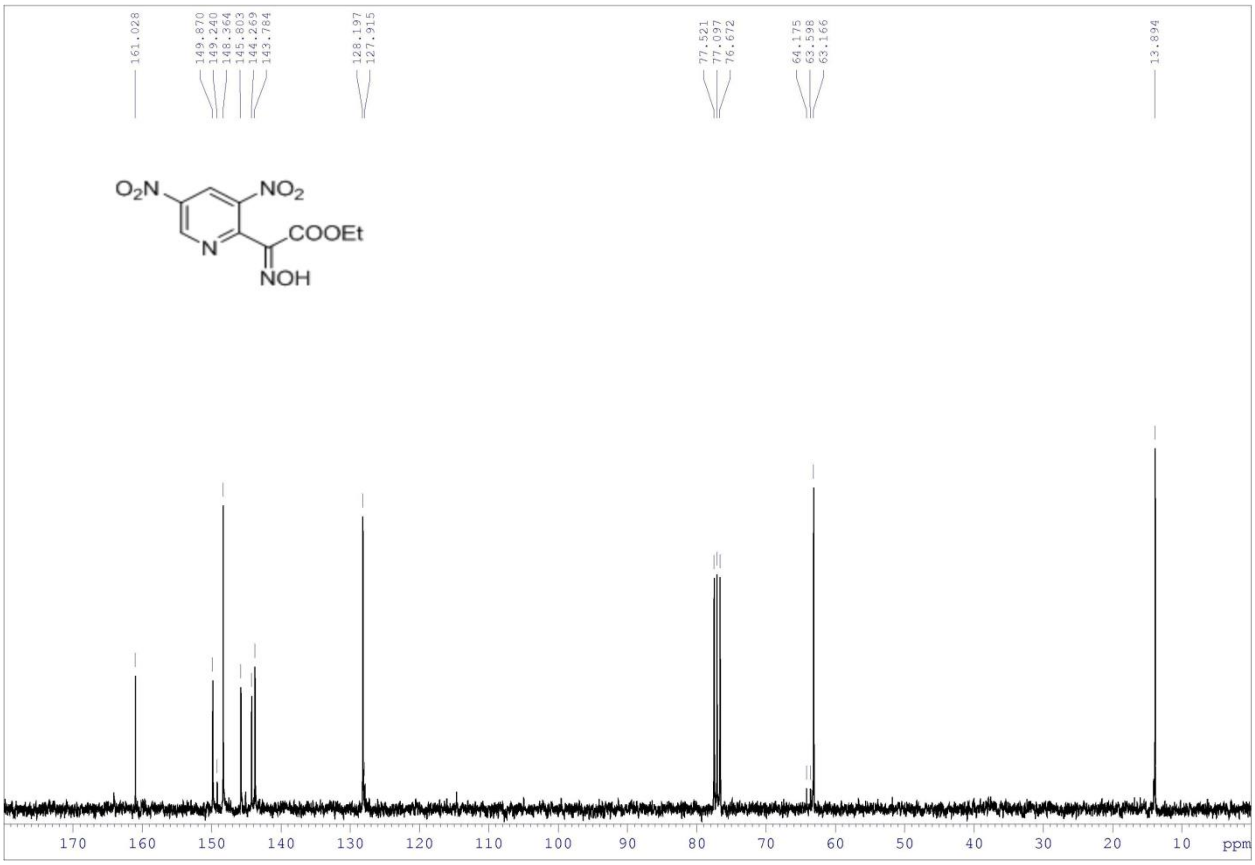

^{1}H and ^{13}C NMR spectra of compound **3b** in $CDCl_3$

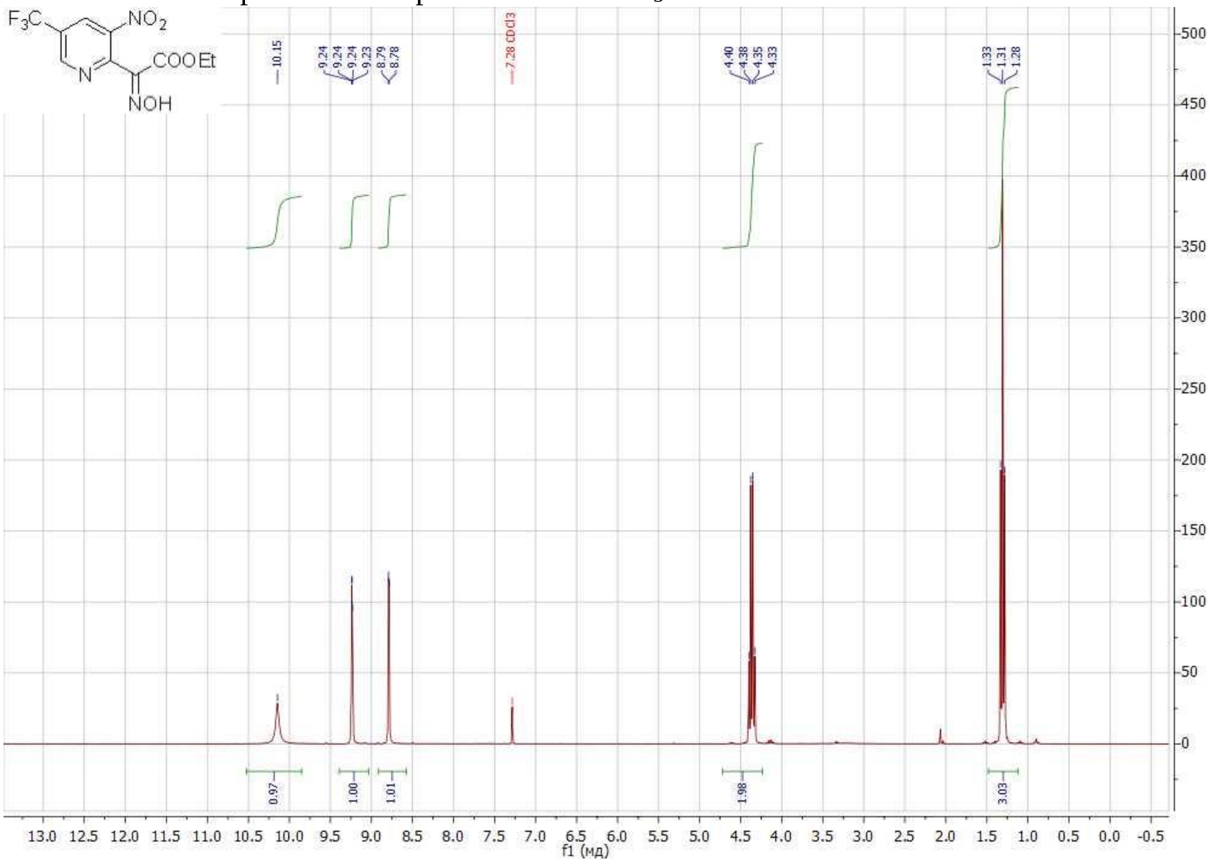

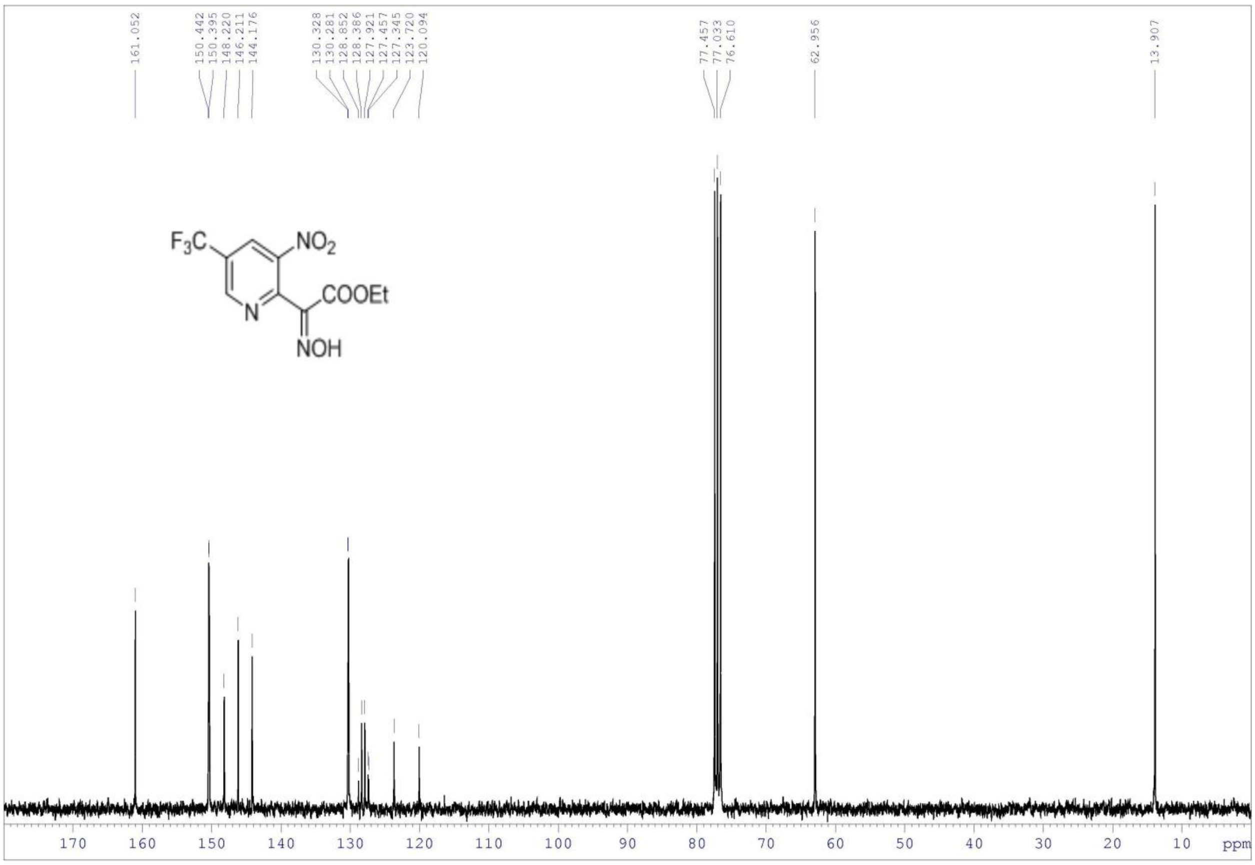

^{1}H and ^{13}C NMR spectra of compound **3c** in $CDCl_3$

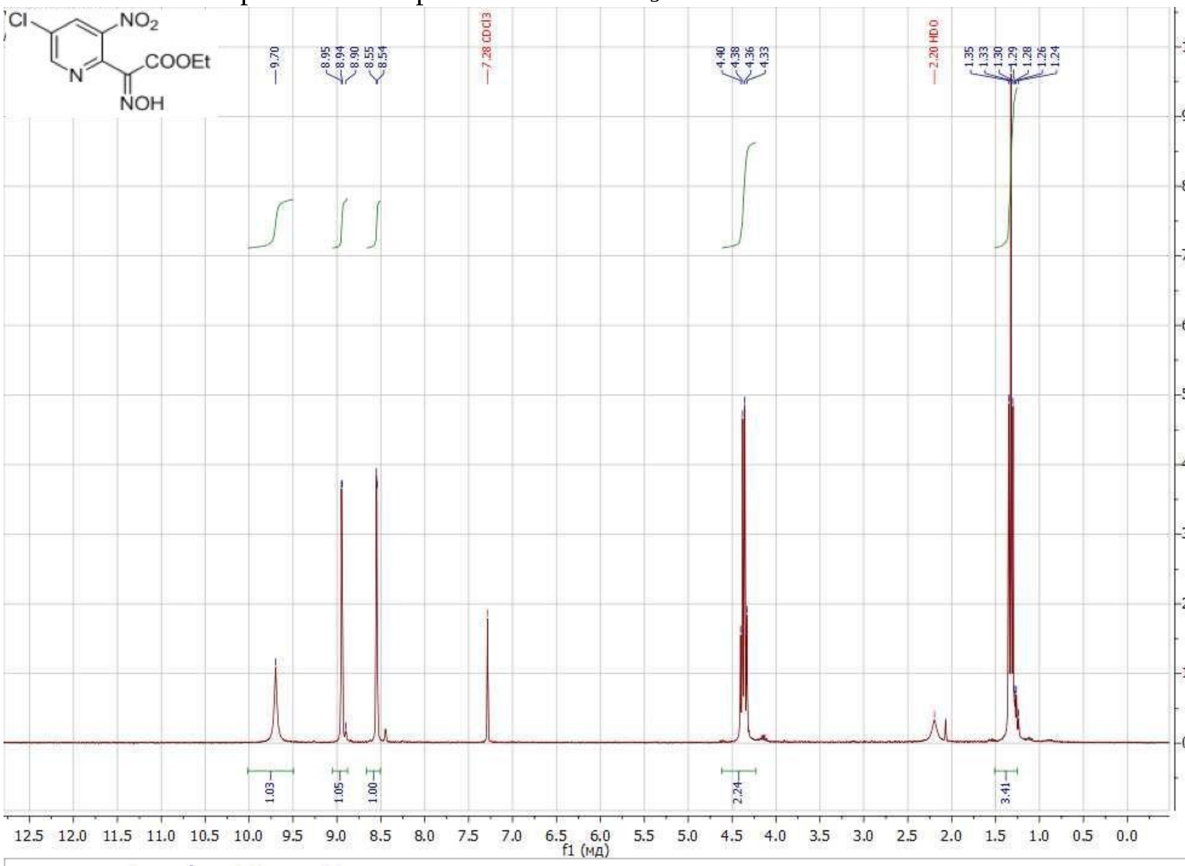

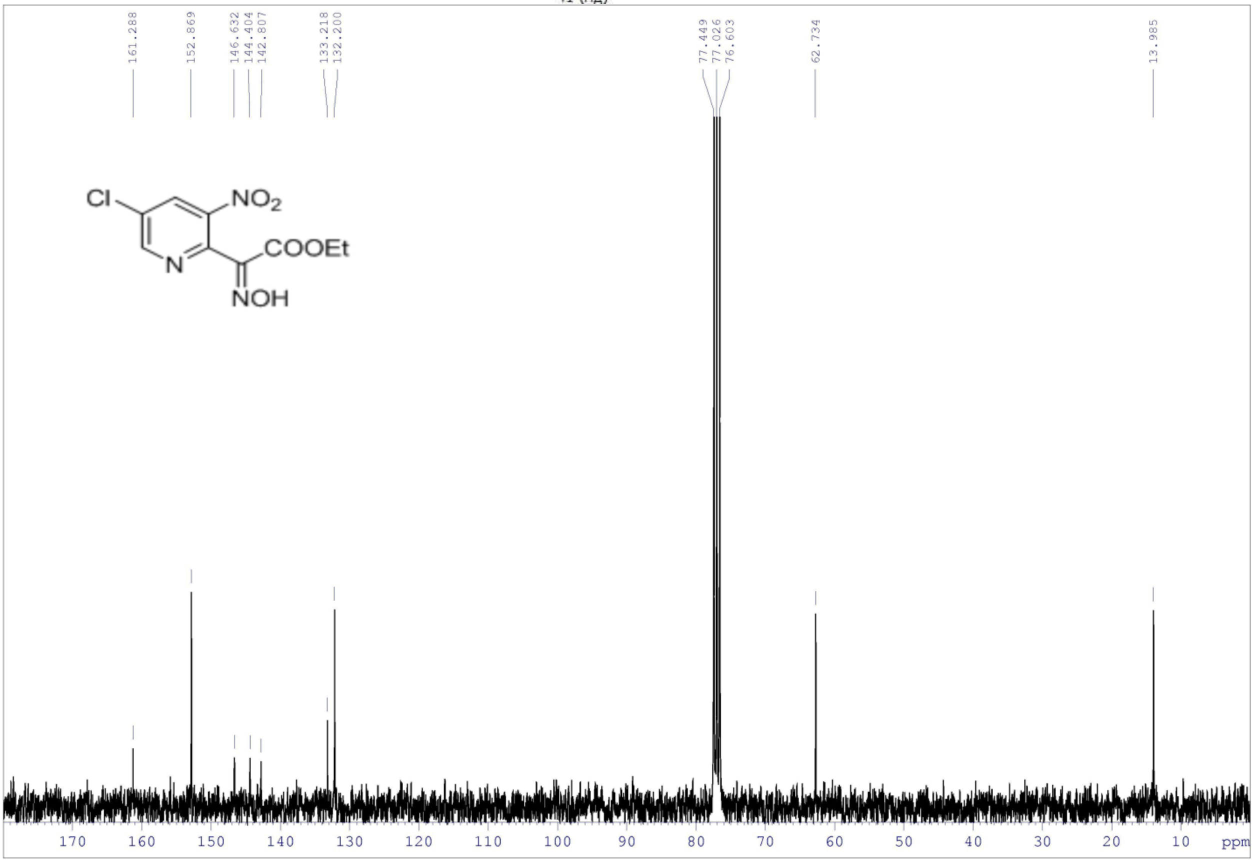

^{1}H and ^{13}C NMR spectra of compound **4a** in $CDCl_3$

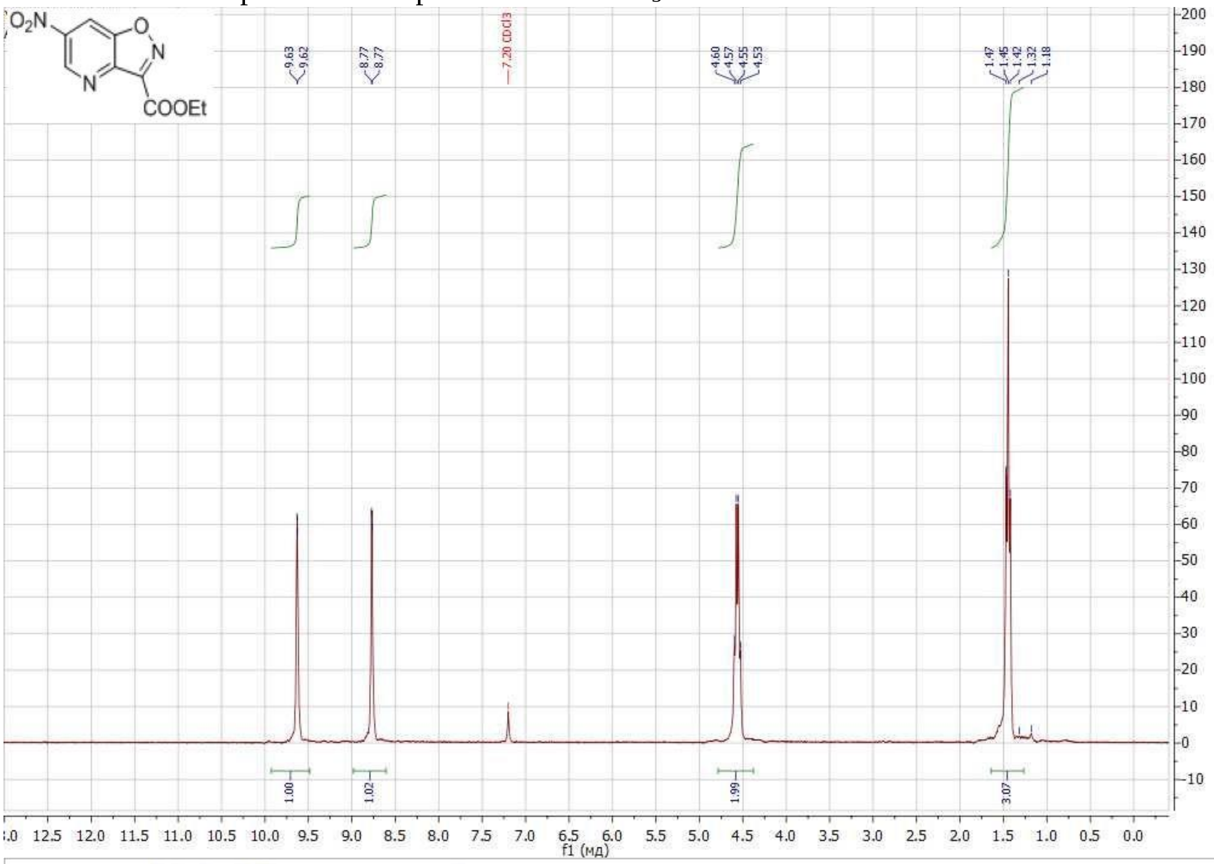

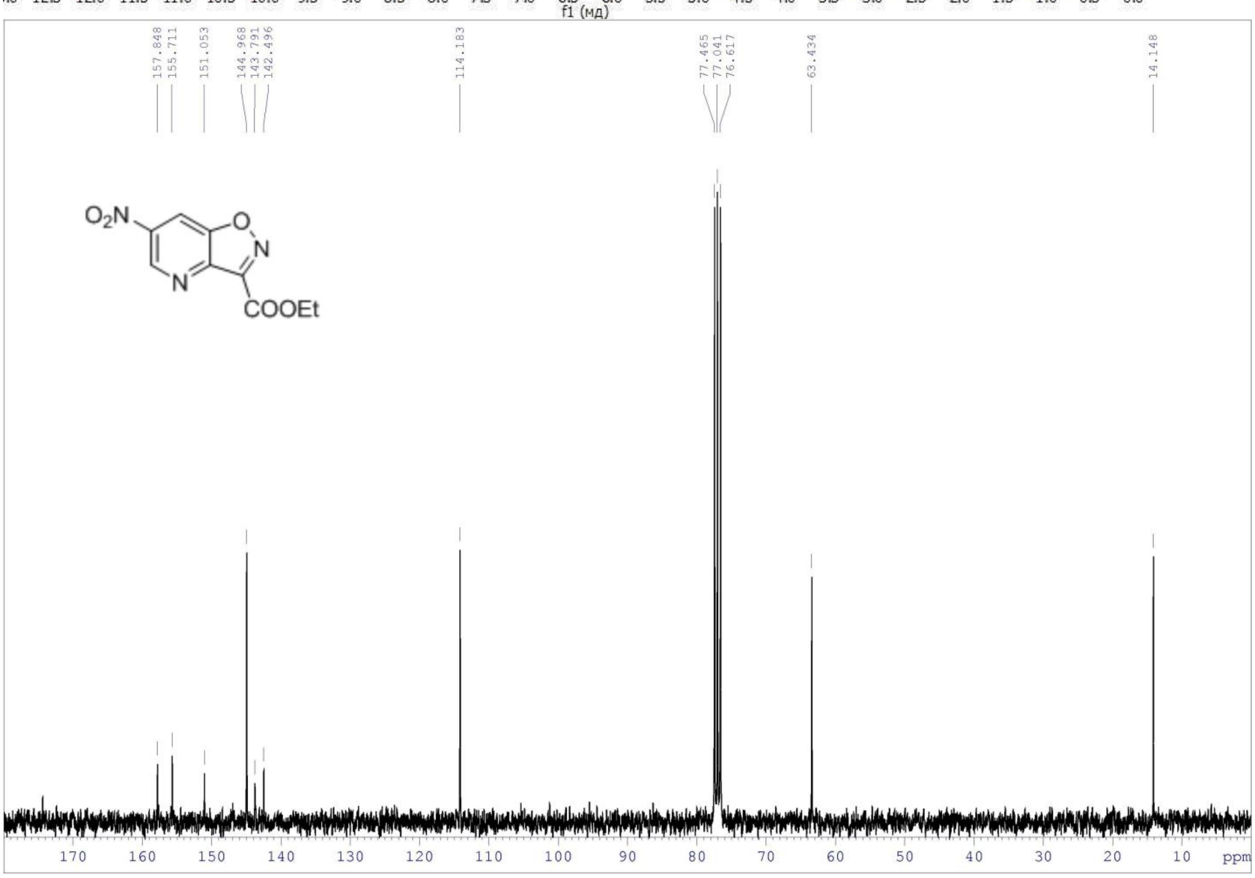

^{1}H and ^{13}C NMR spectra of compound **4b** in $CDCl_3$

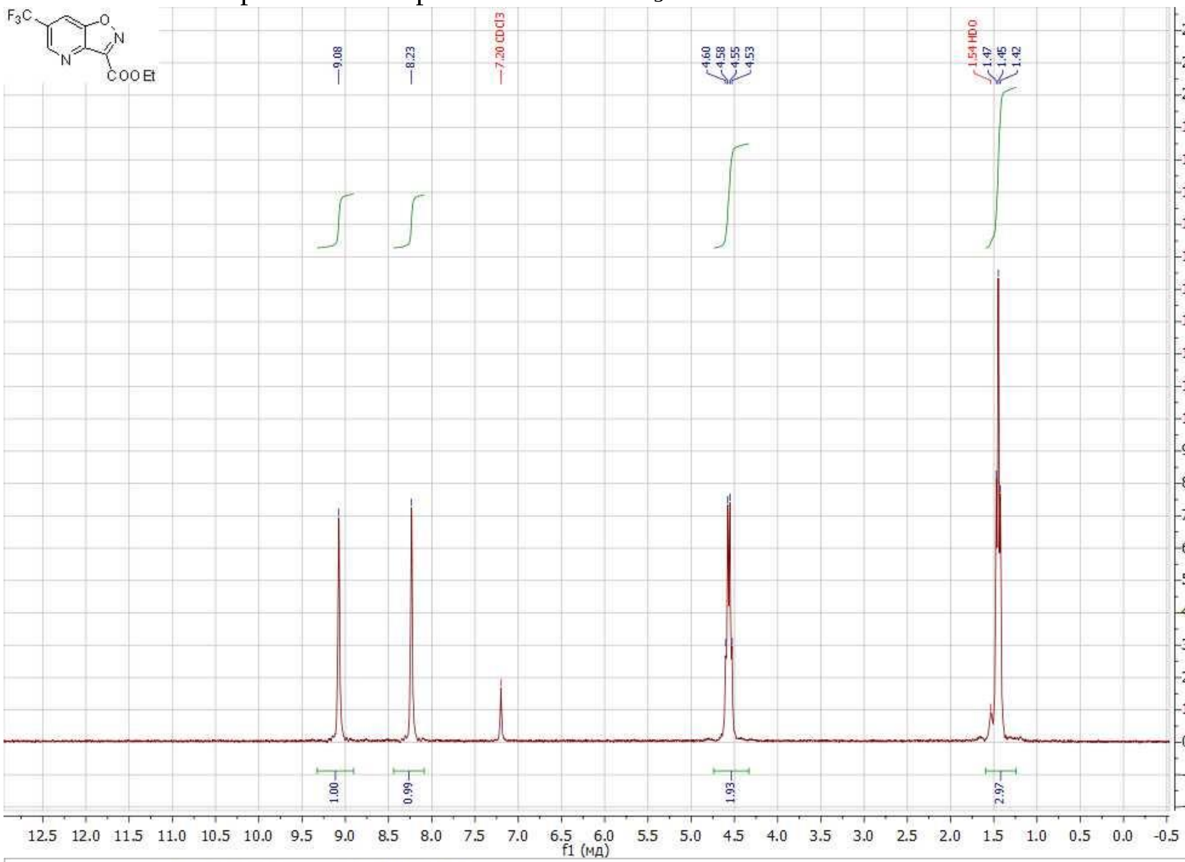

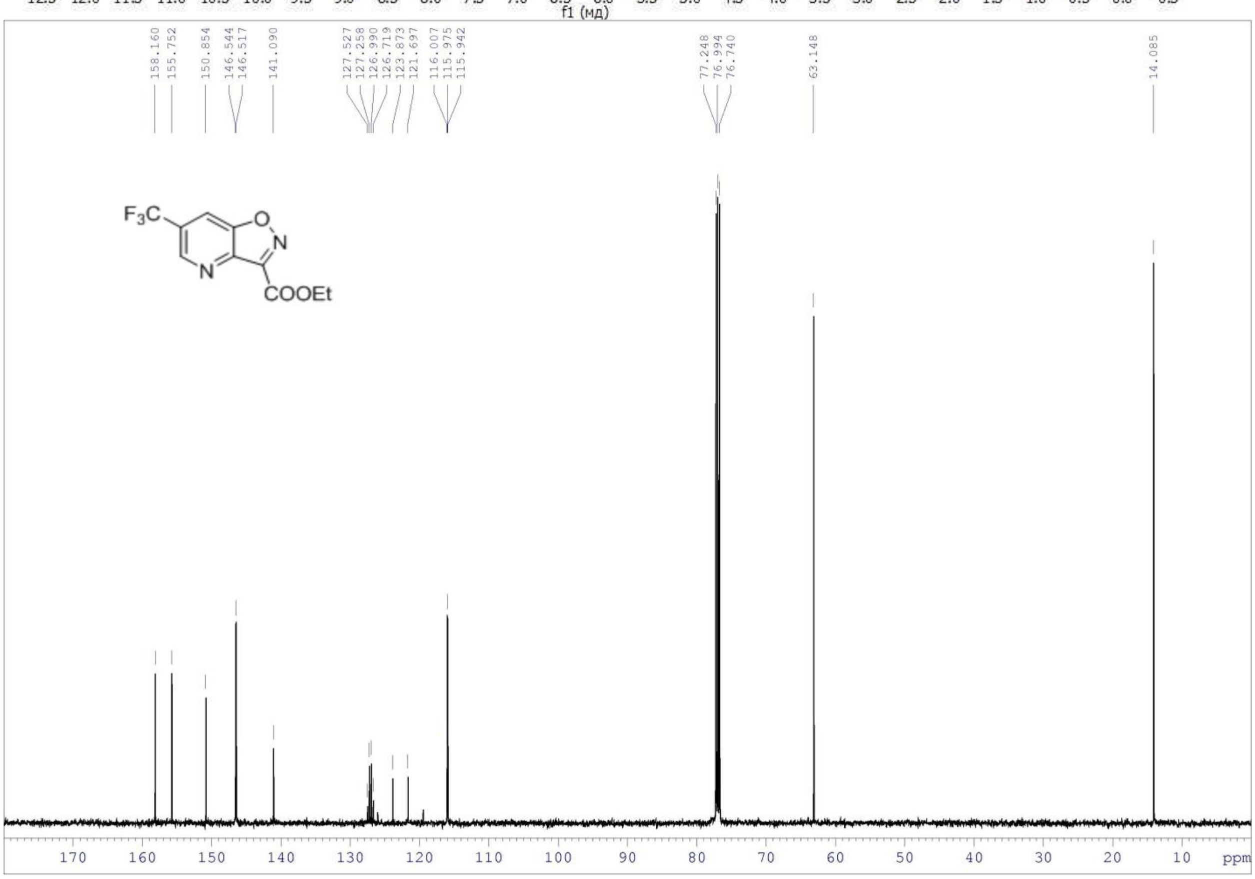

^{1}H and ^{13}C NMR spectra of compound **4c** in $CDCl_3$

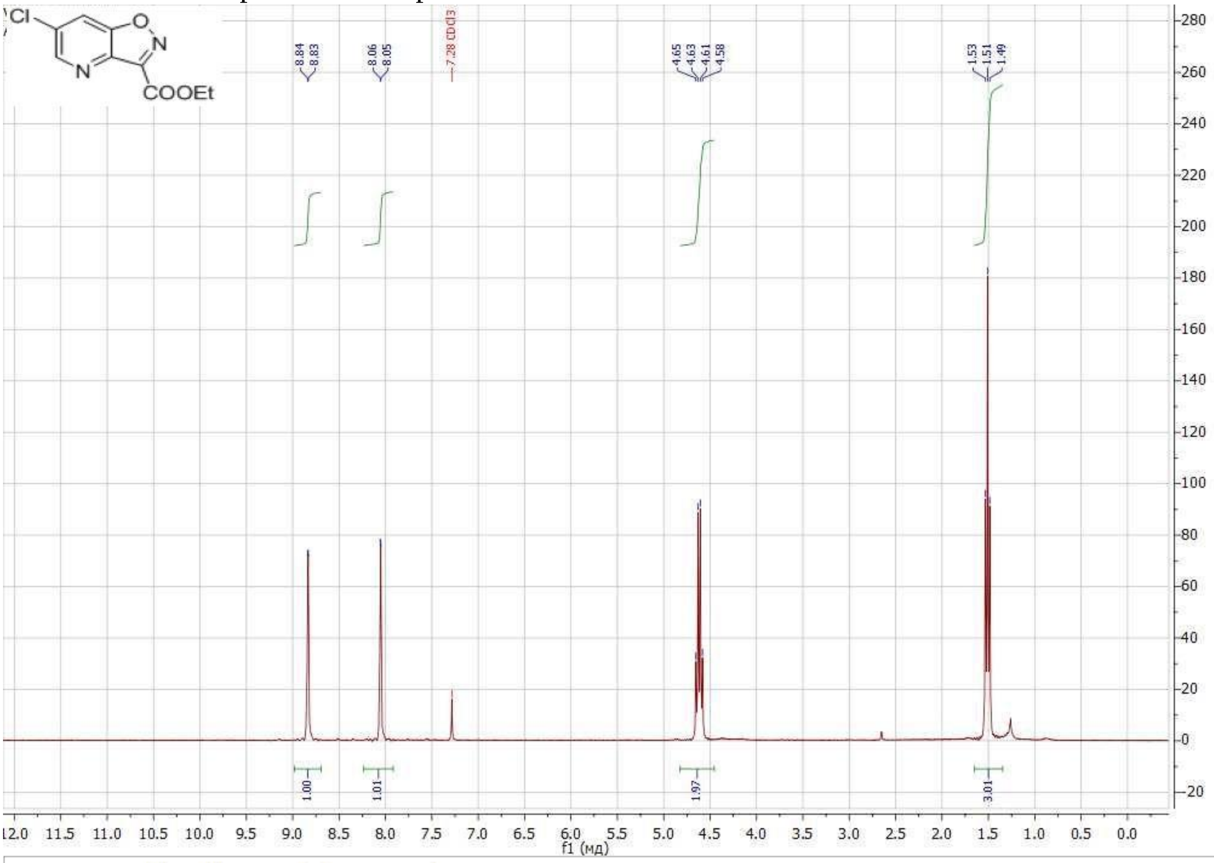

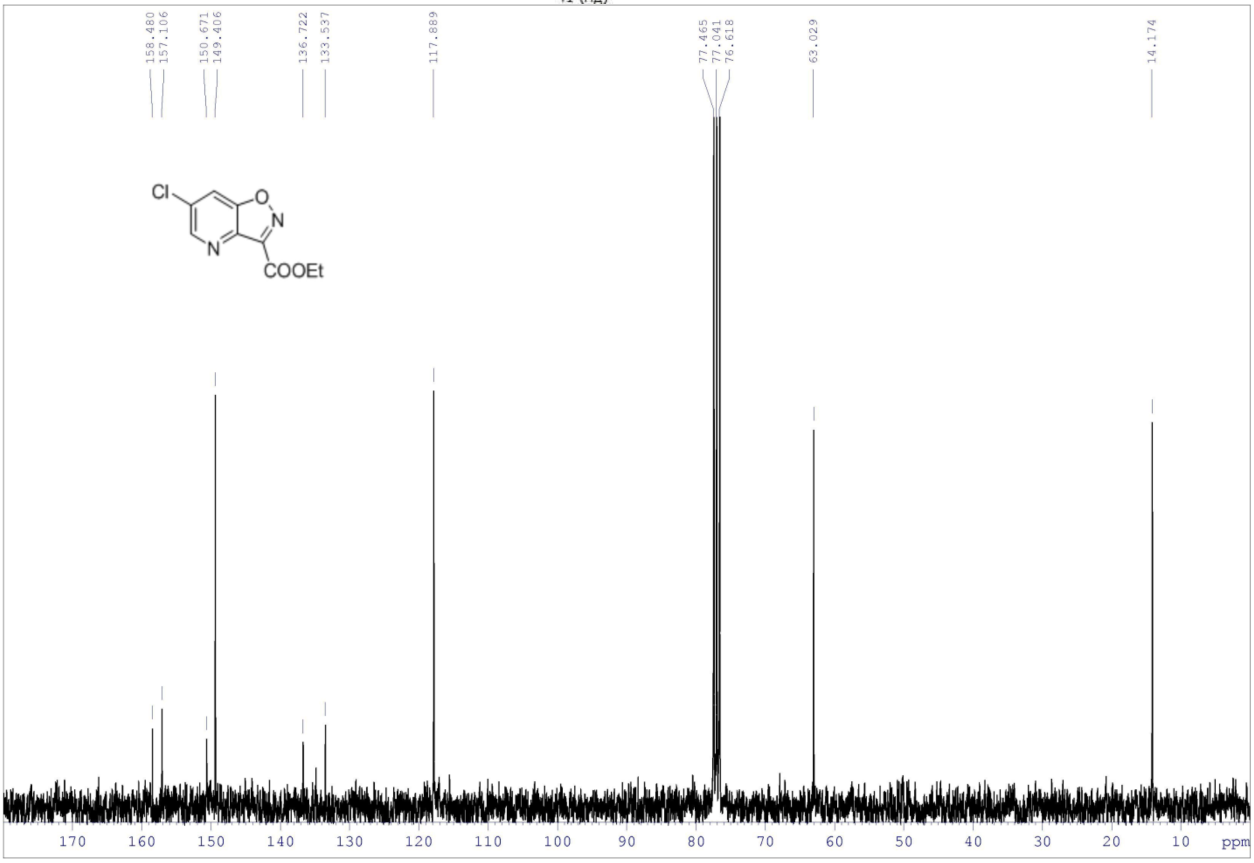

^{1}H and ^{13}C NMR spectra of compound **6a** in $CDCl_3$

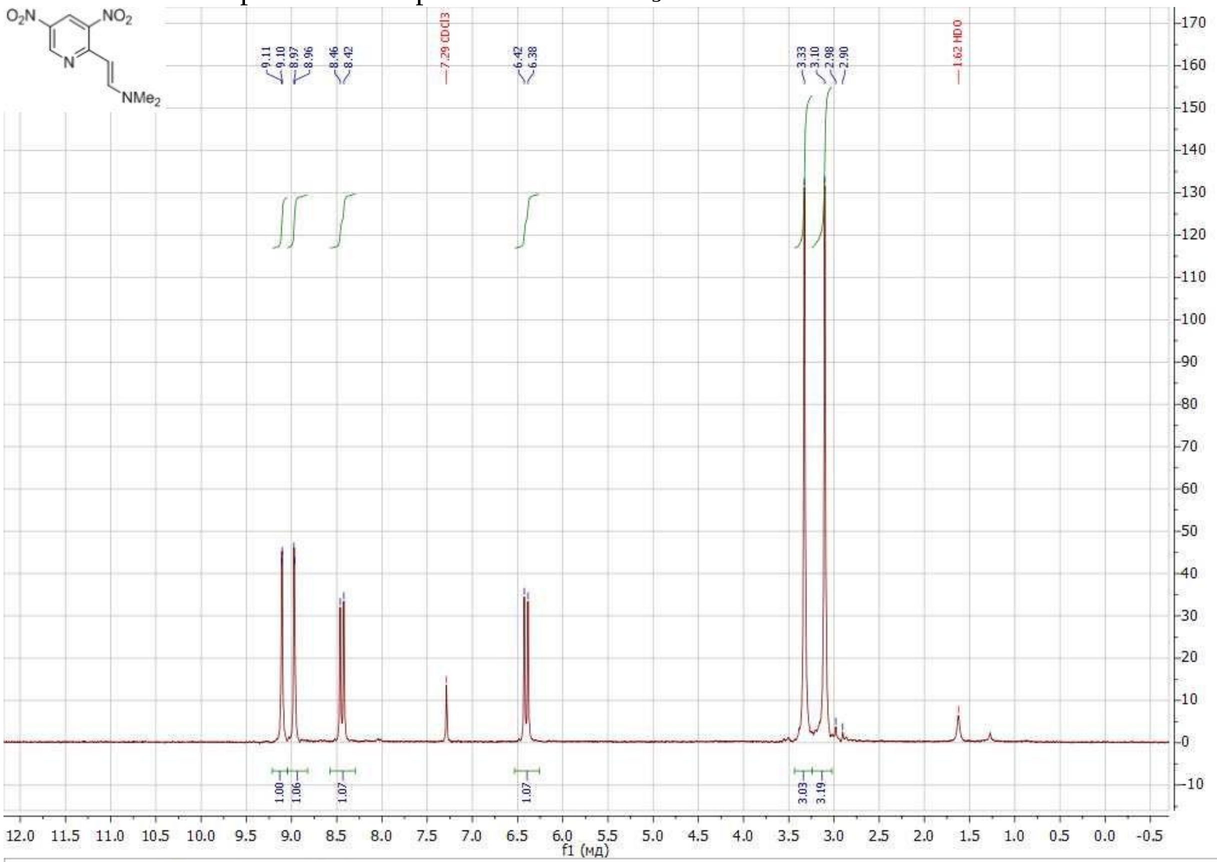

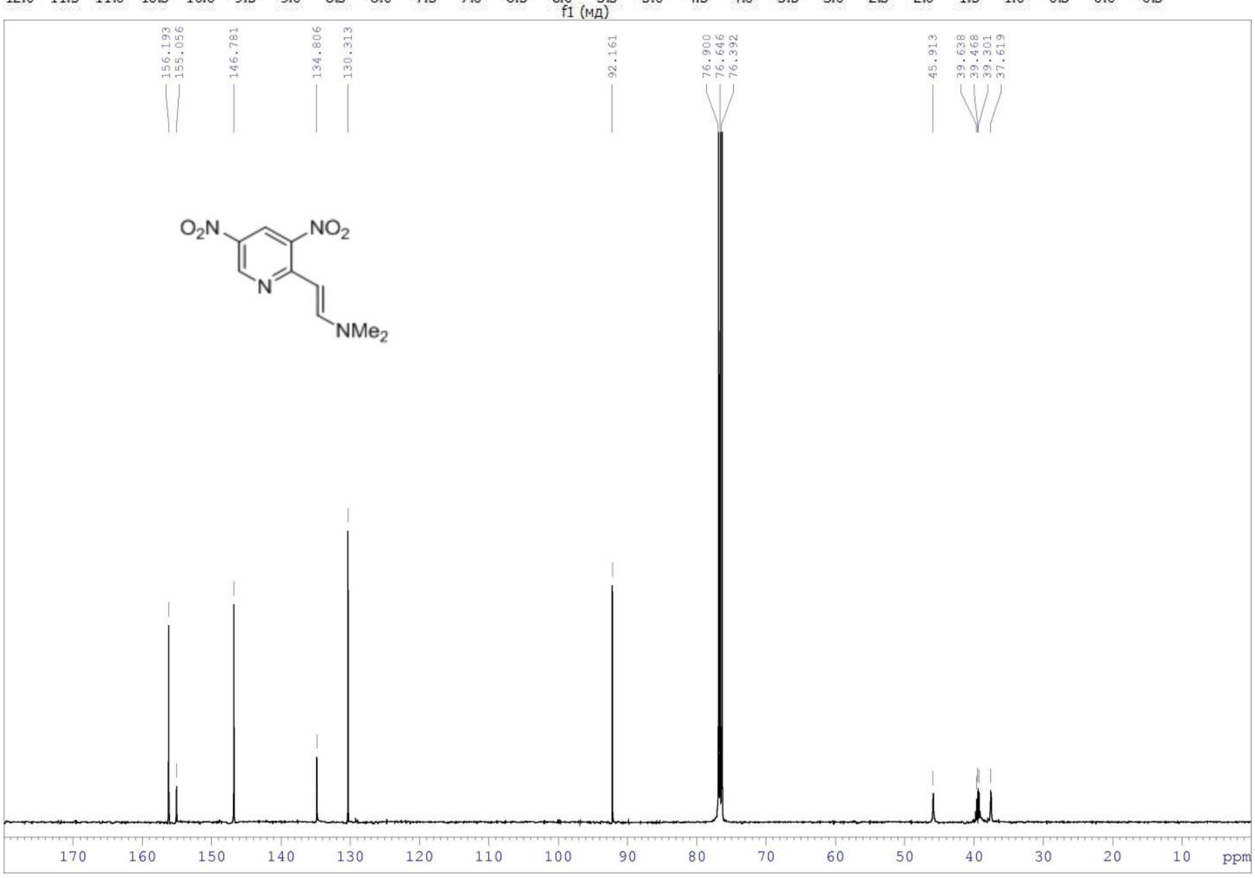

^{1}H and ^{13}C NMR spectra of compound **6b** in $CDCl_3$

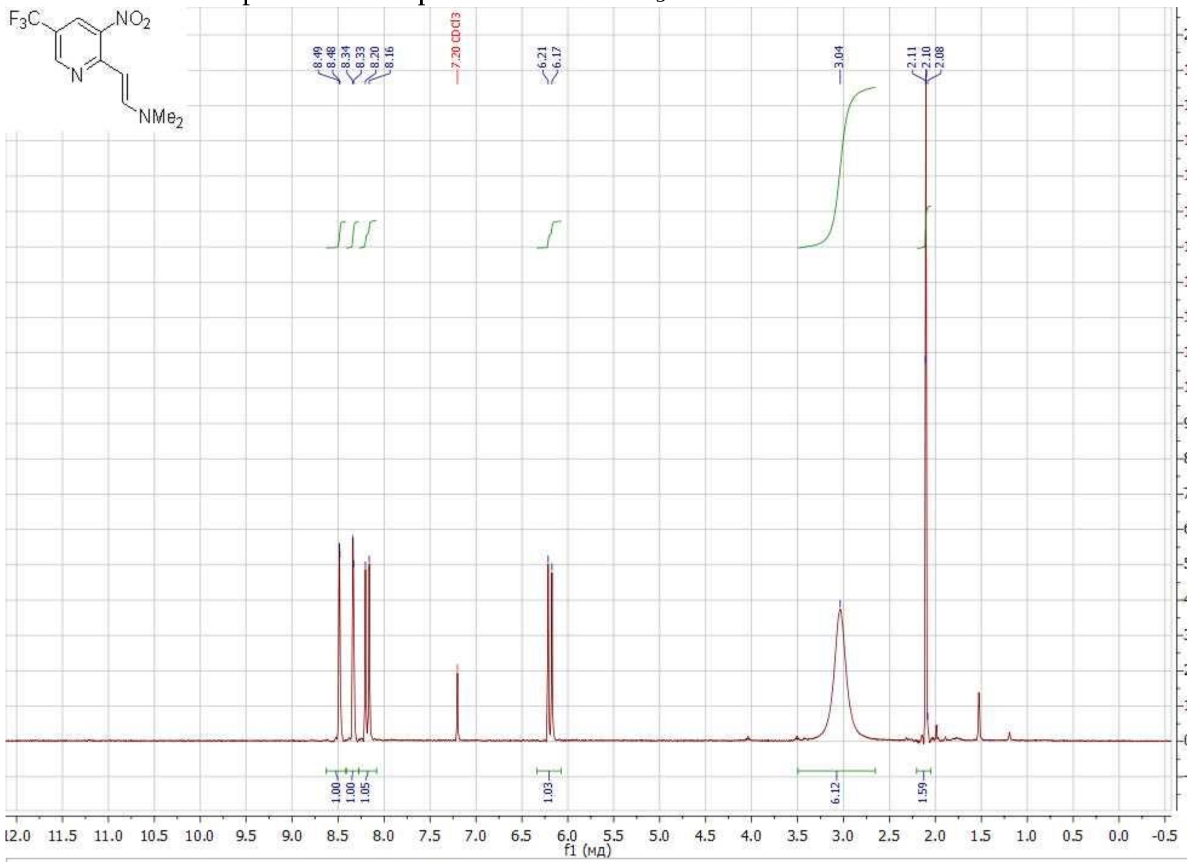

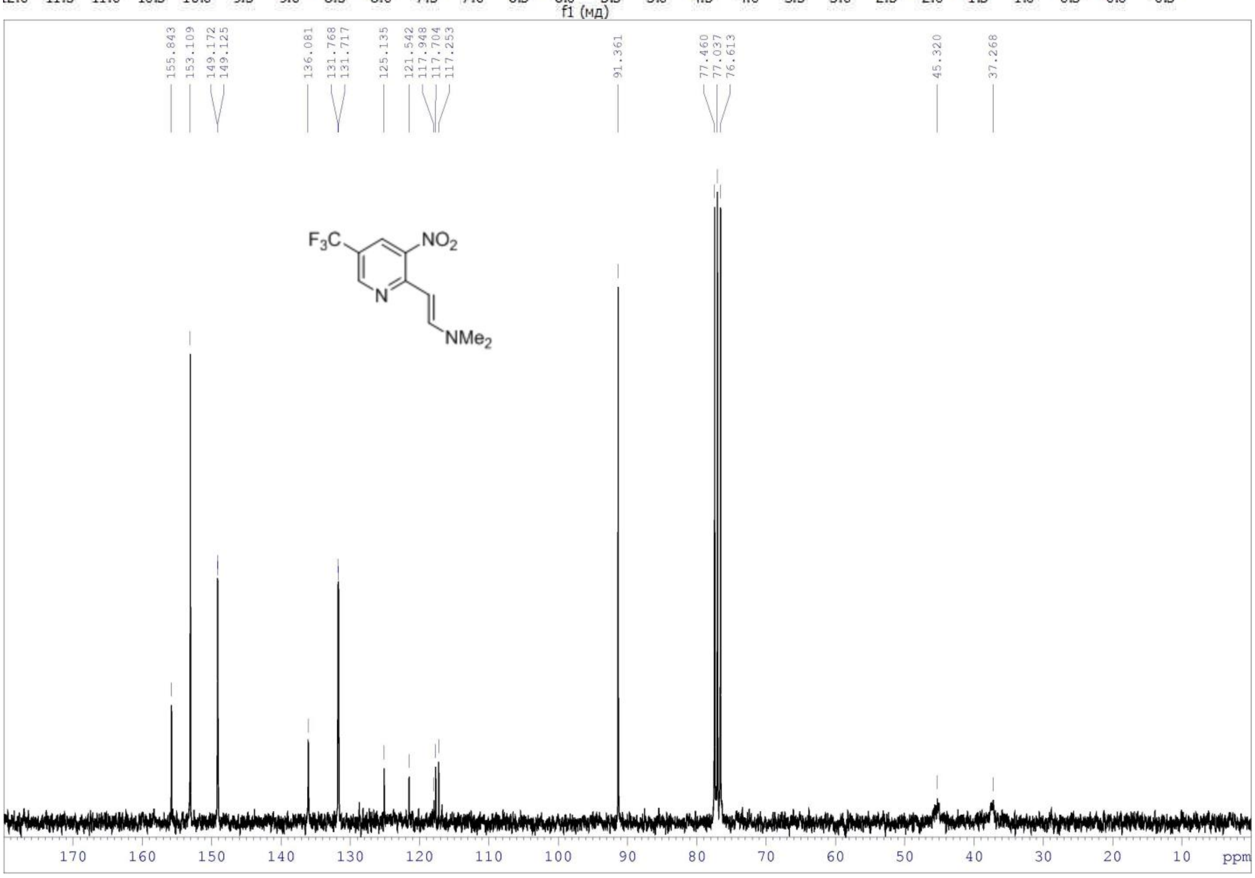

^{1}H and ^{13}C NMR spectra of compound **6c** in $CDCl_3$

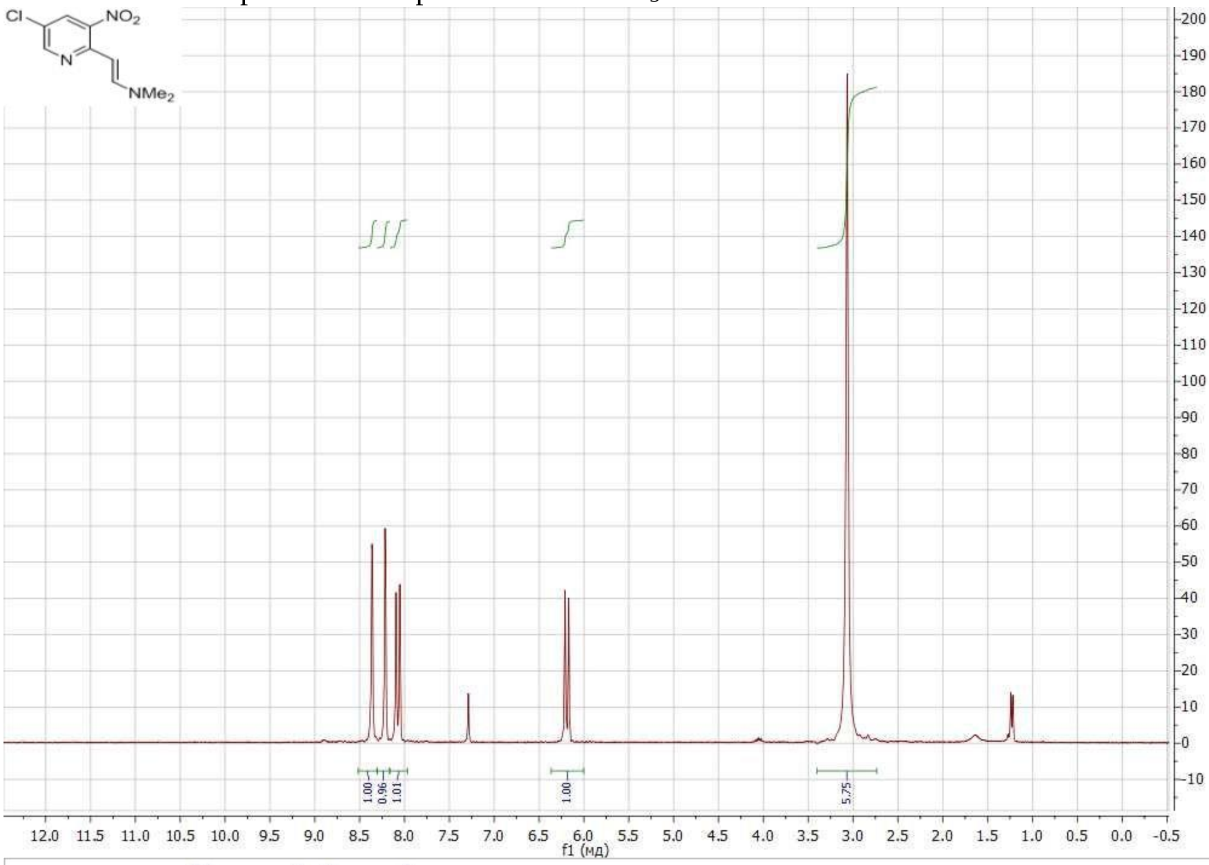

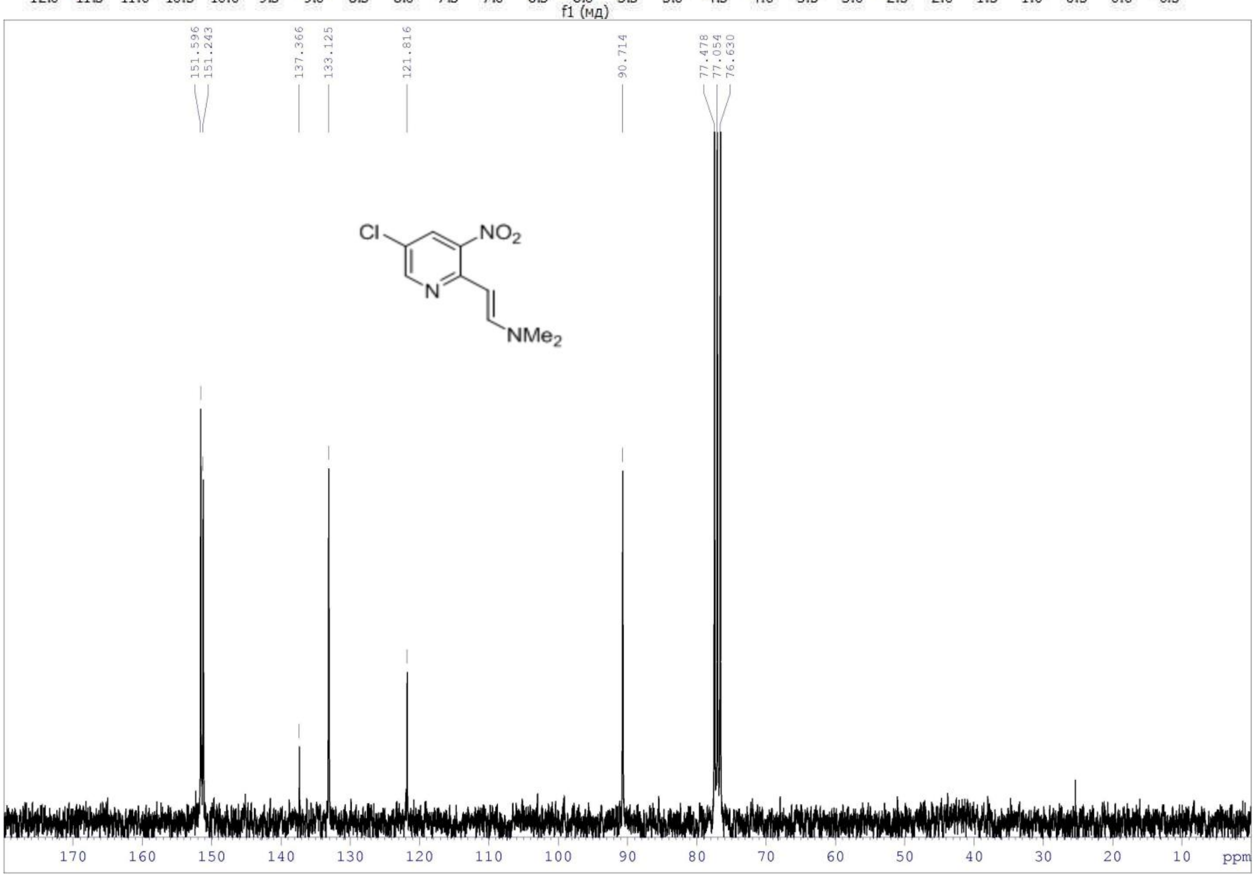

^{1}H and ^{13}C NMR spectra of compound **7a** in DMSO-d_6

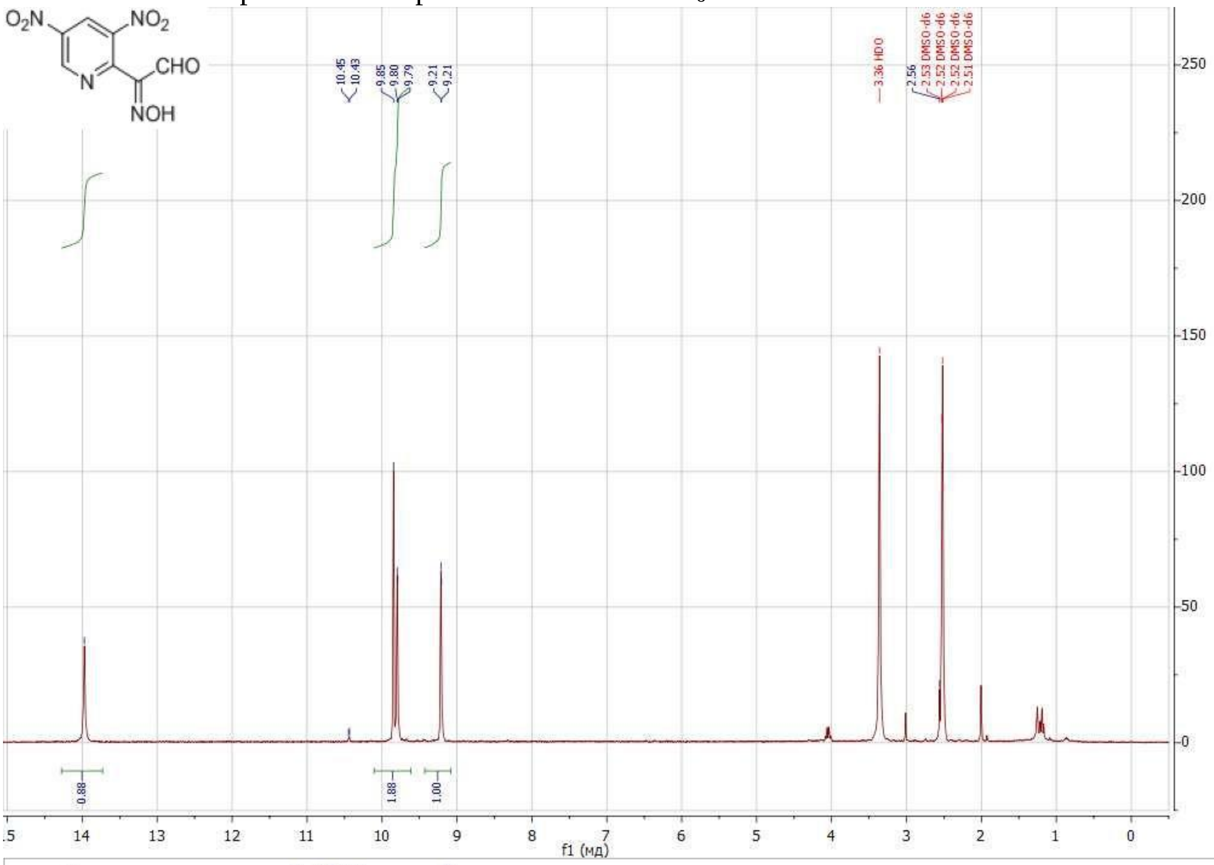

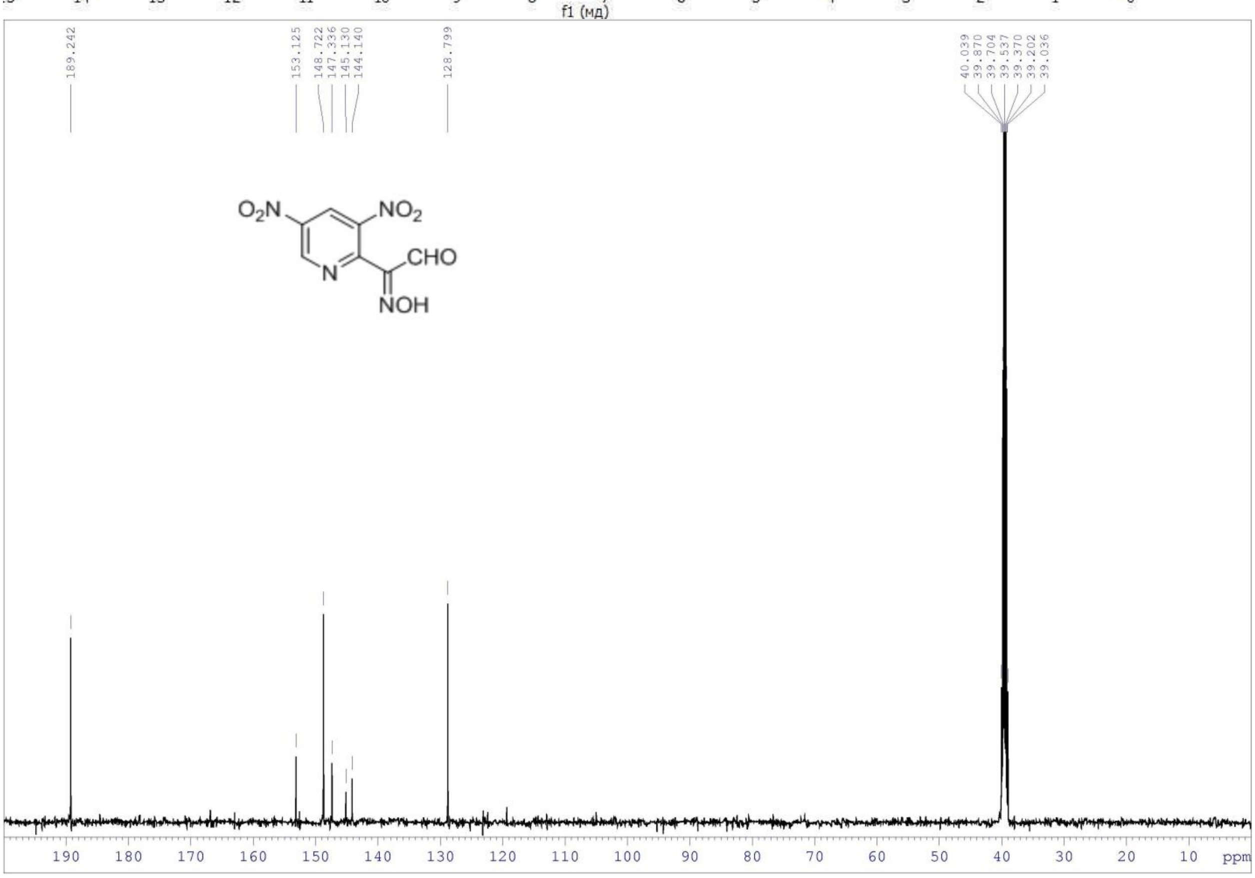

^{1}H and ^{13}C NMR spectra of compound **7b** in $CDCl_3$

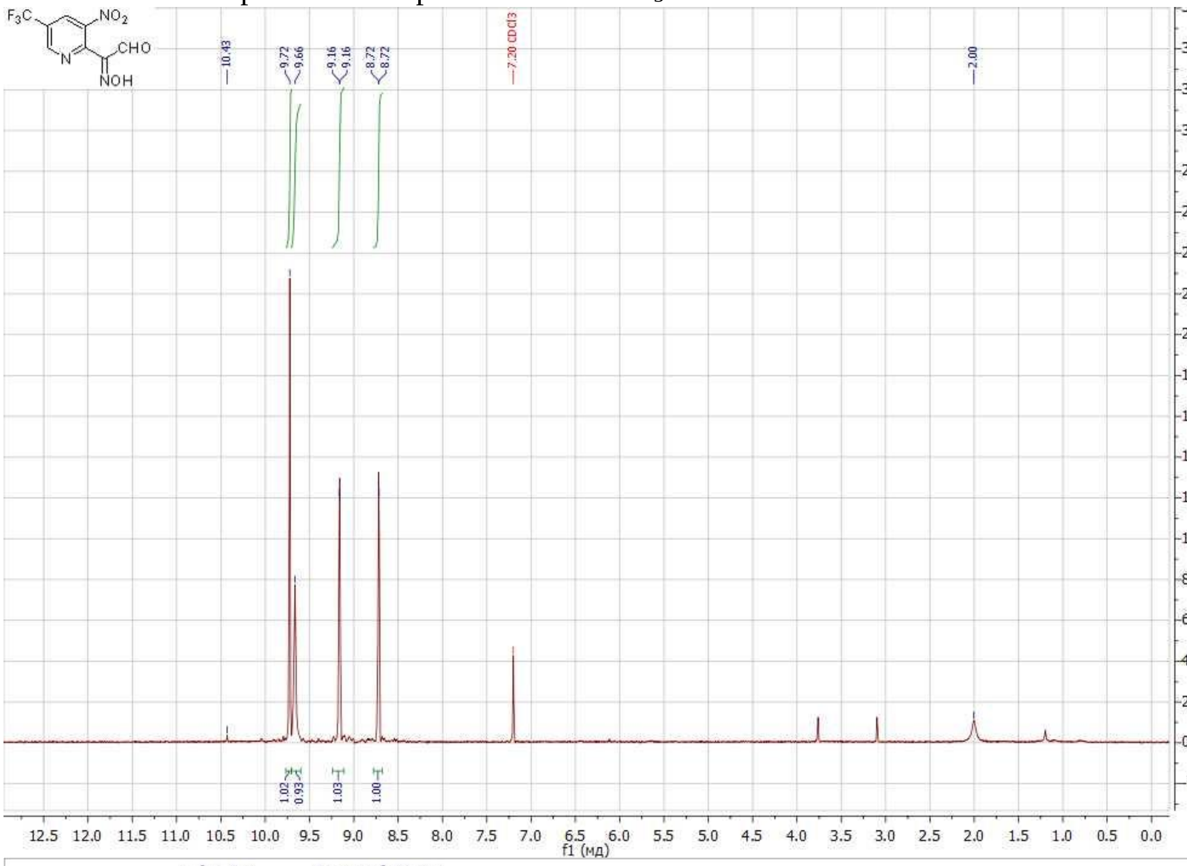

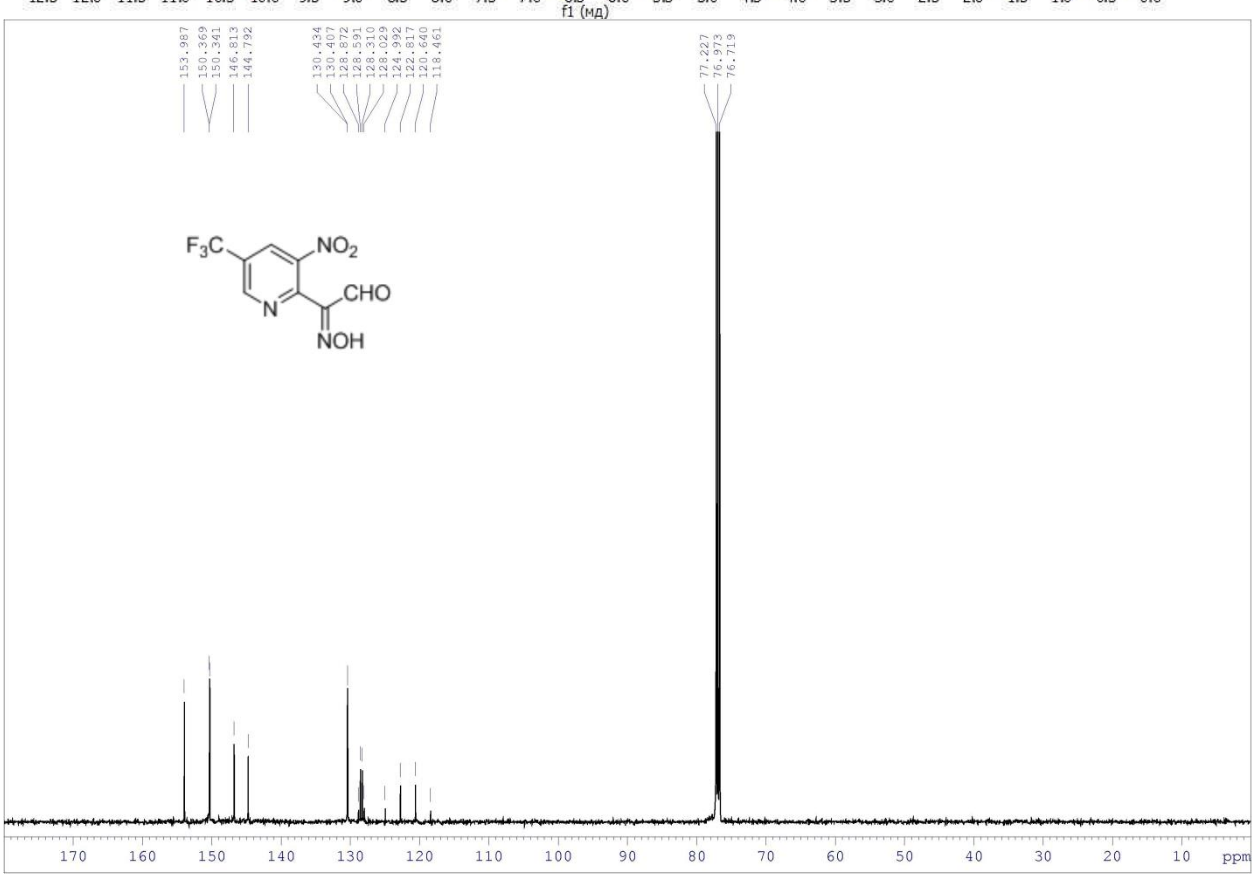

^{1}H and ^{13}C NMR spectra of compound **7c** in DMSO-d_6

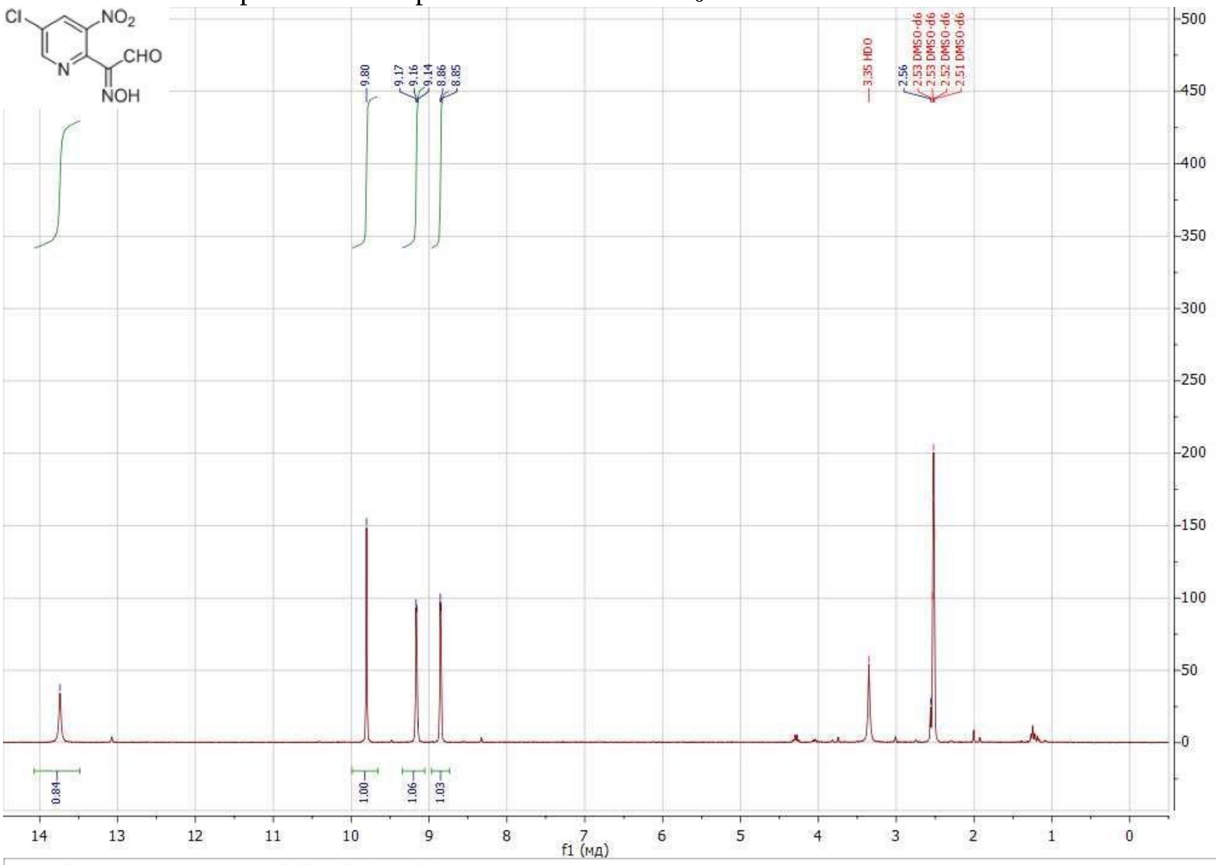

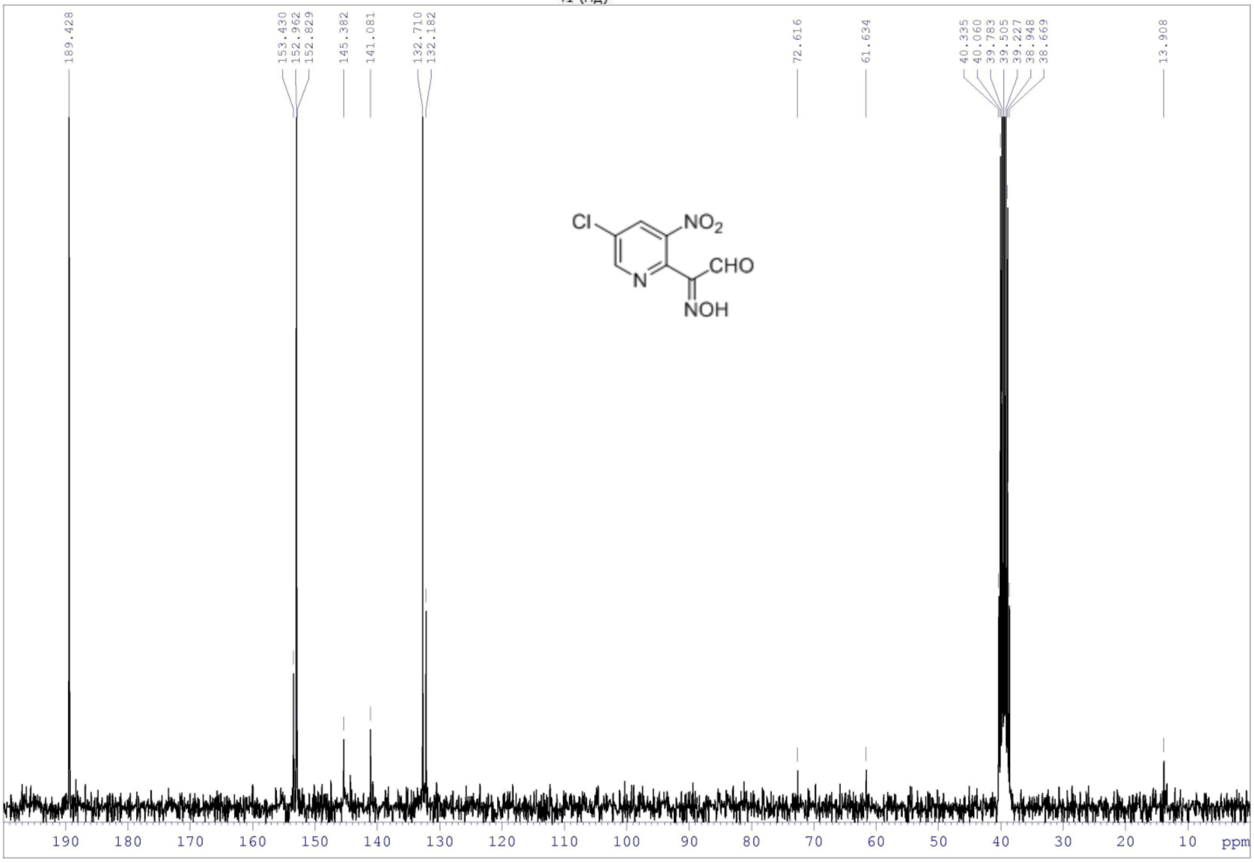

^{1}H and ^{13}C NMR spectra of compound **8b** in DMSO-d_6

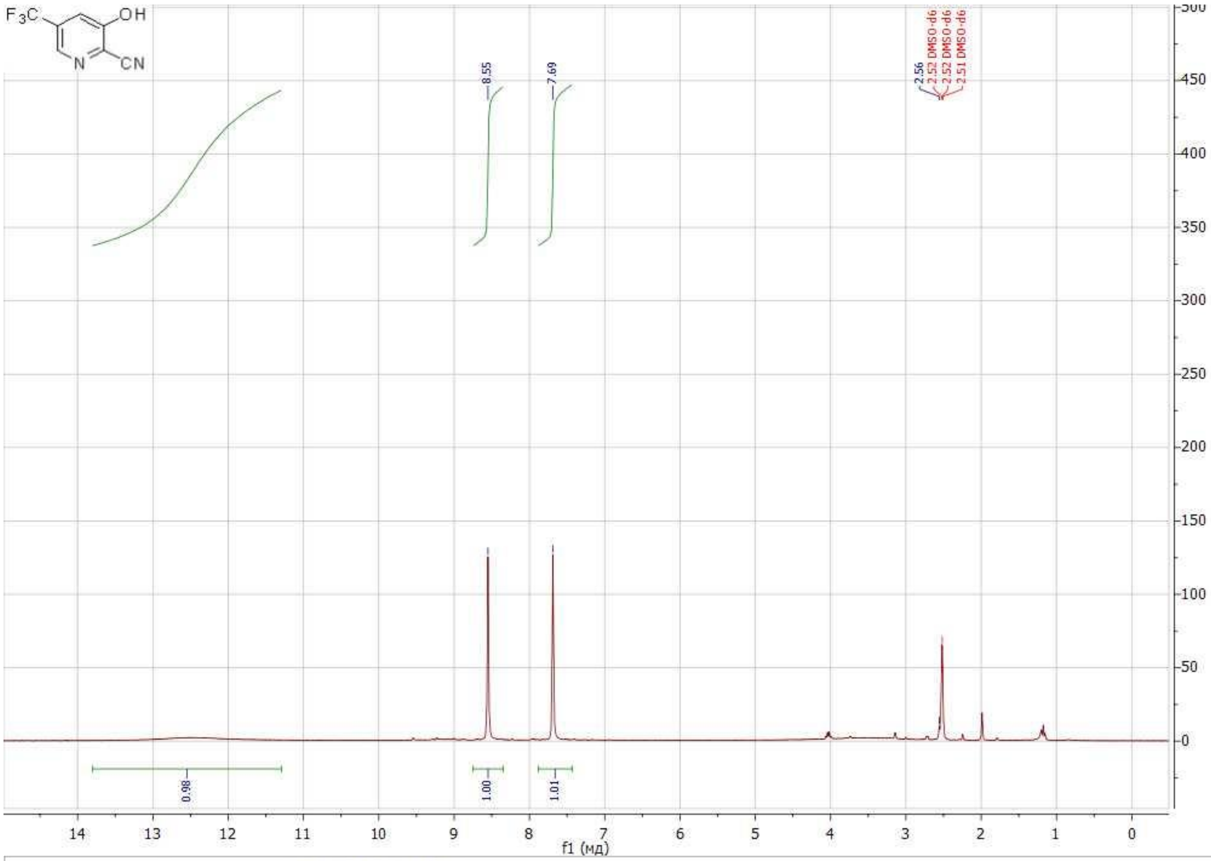

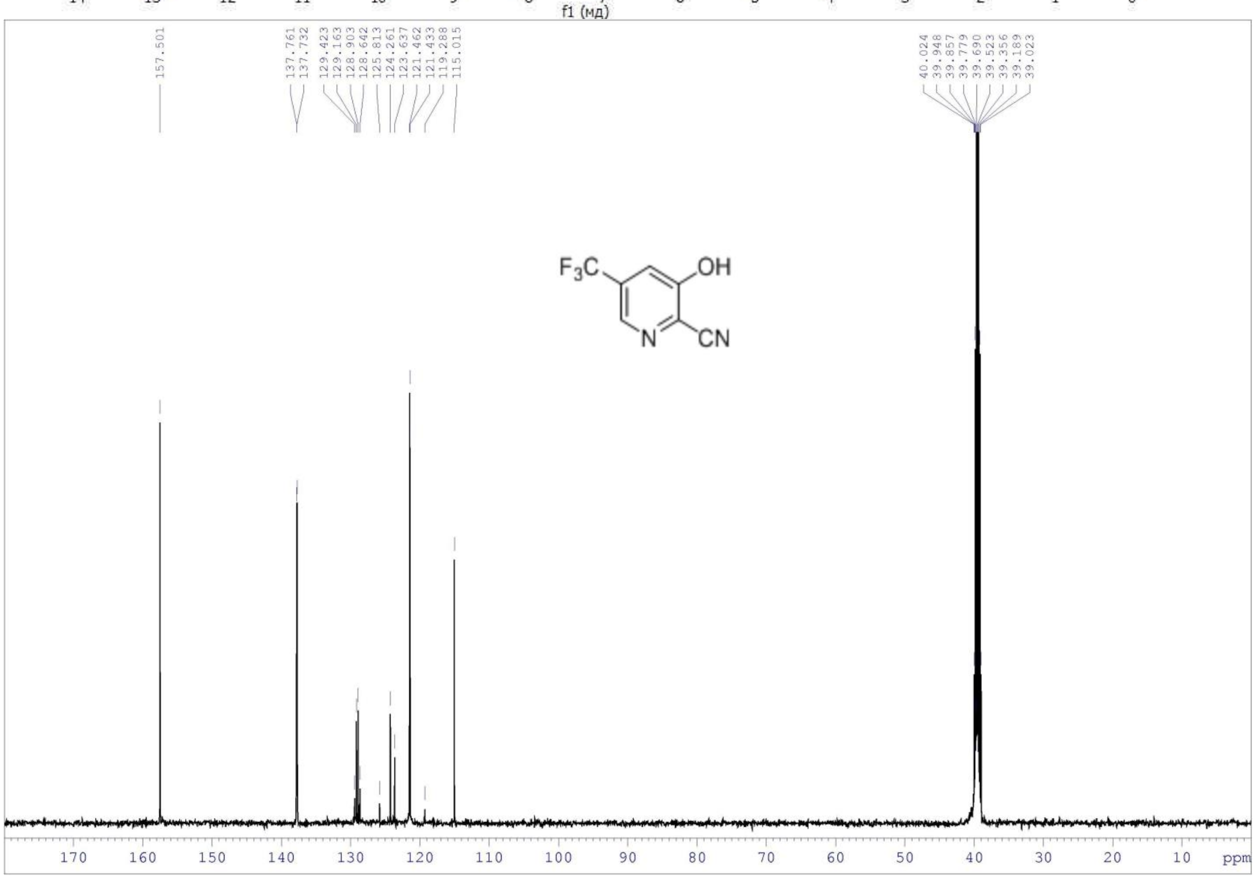

^{1}H and ^{13}C NMR spectra of compound **8c** in DMSO-d_6

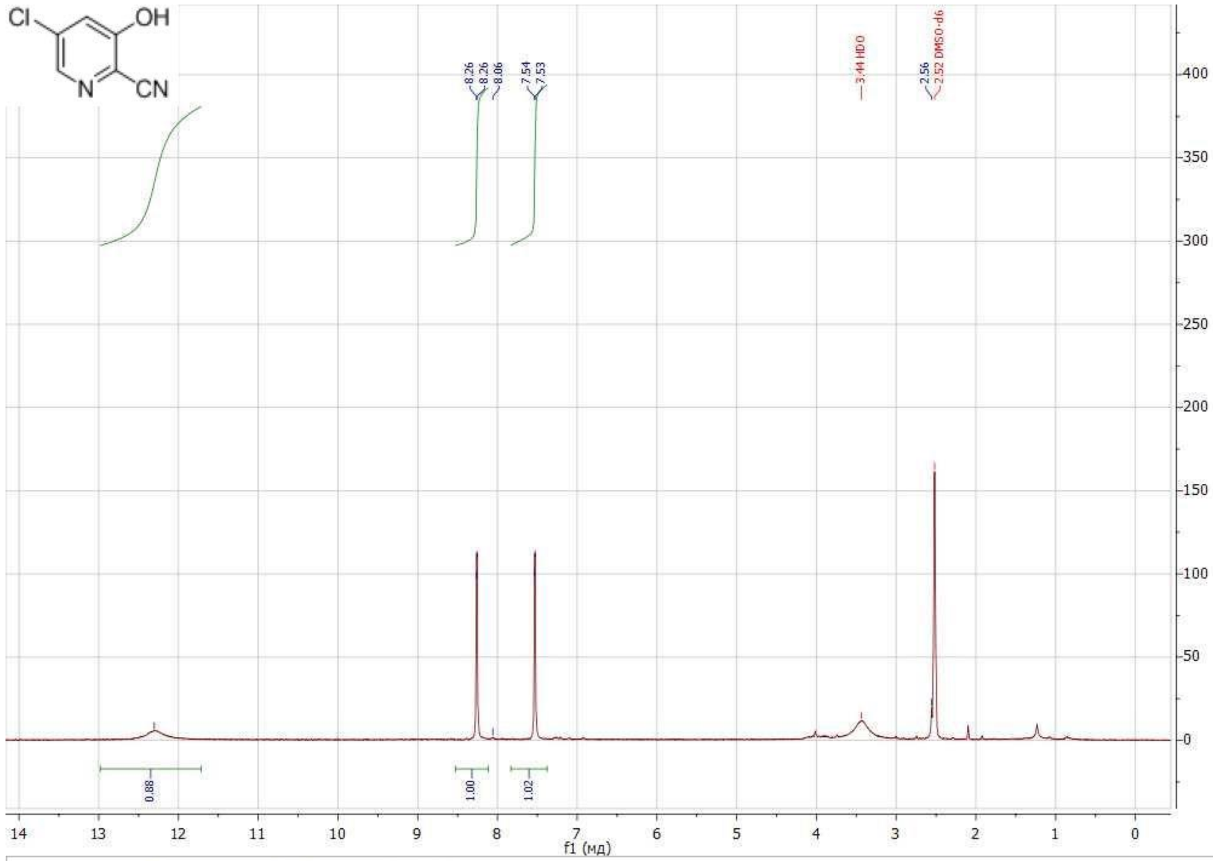

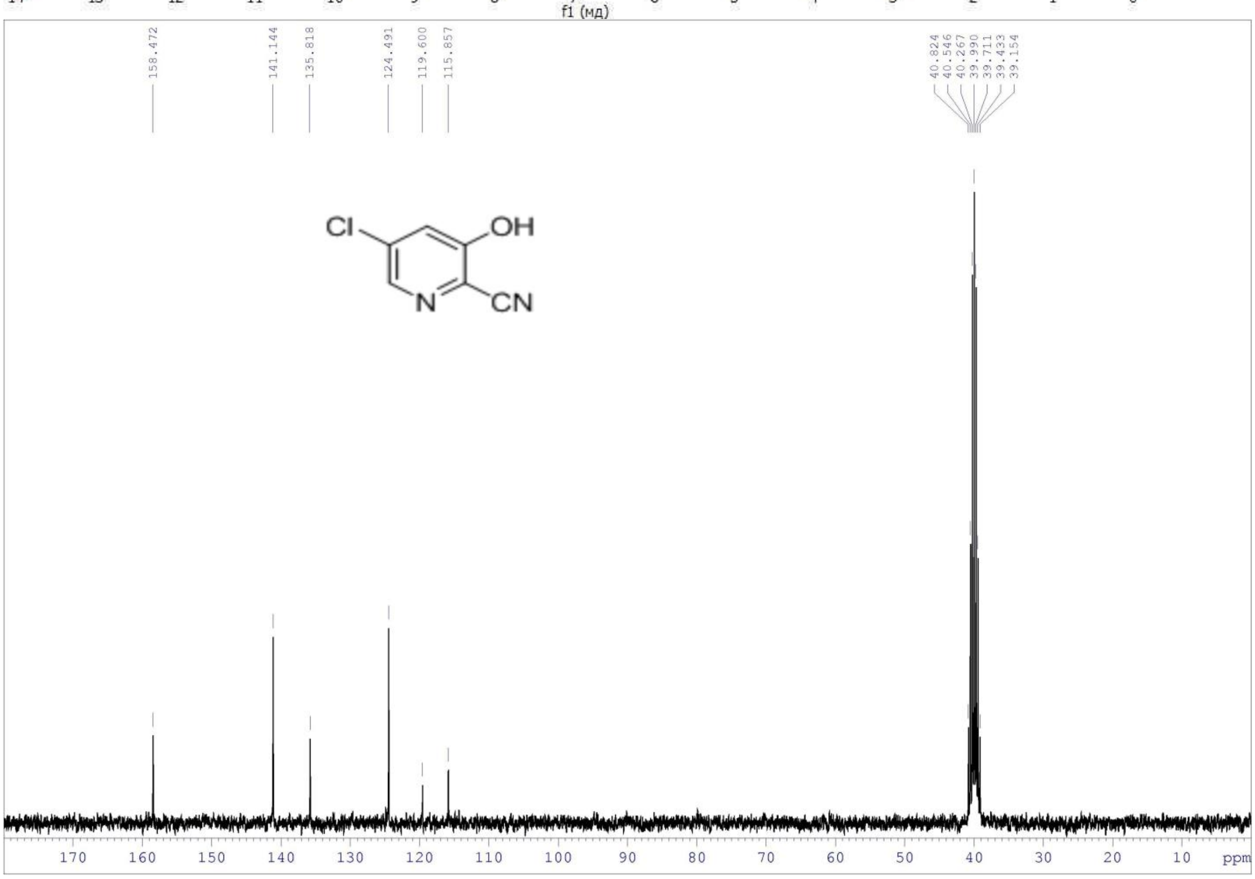

^{1}H and ^{13}C NMR spectra of compound **9a** in DMSO-d_6

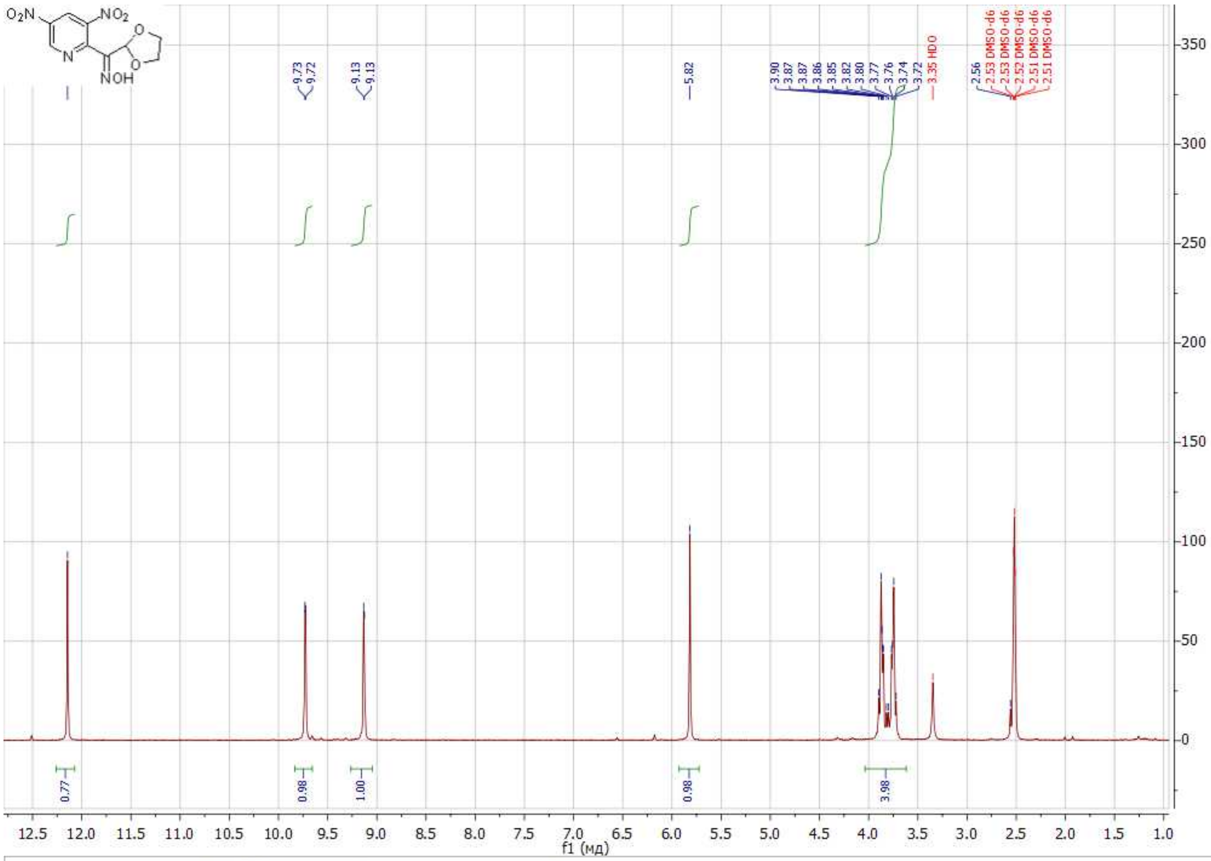

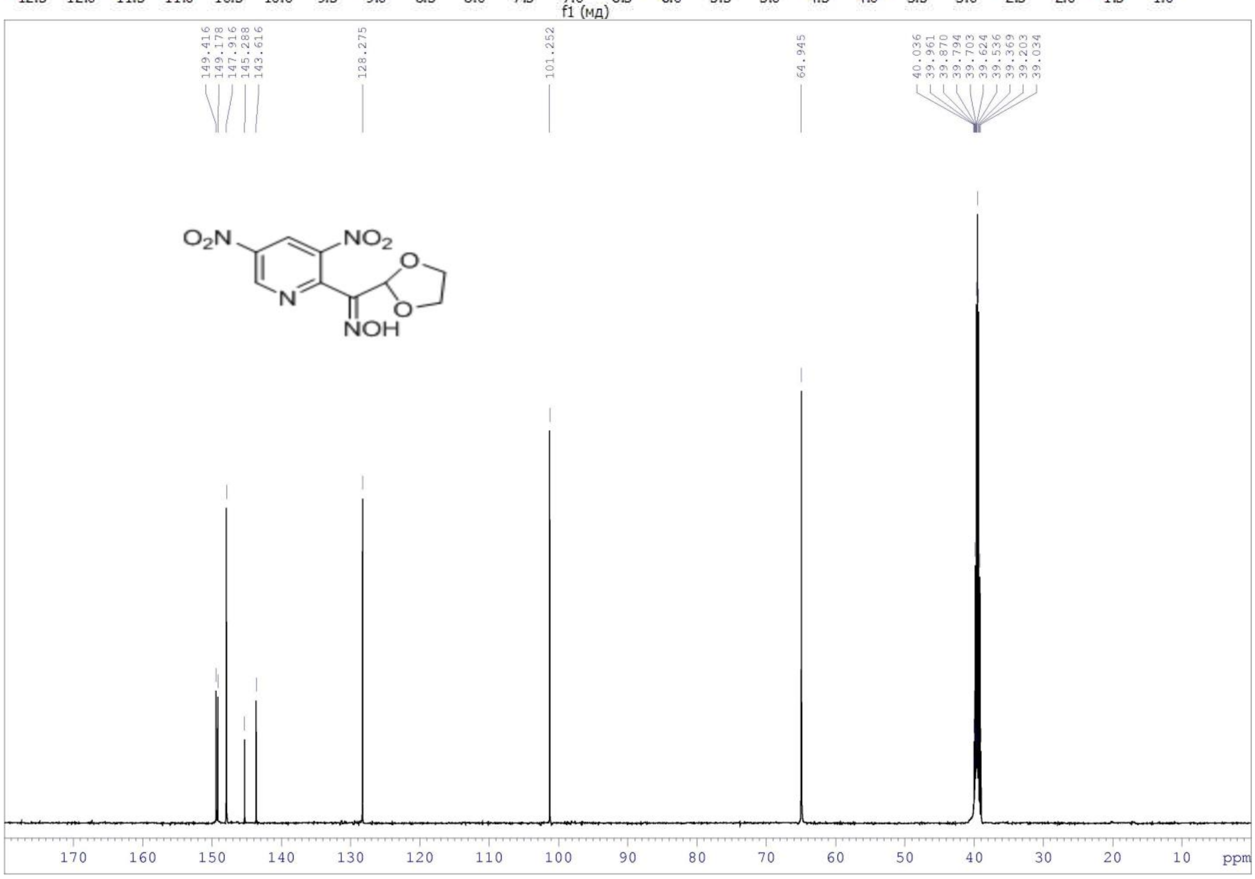

^{1}H and ^{13}C NMR spectra of compound **9b** in $CDCl_6$

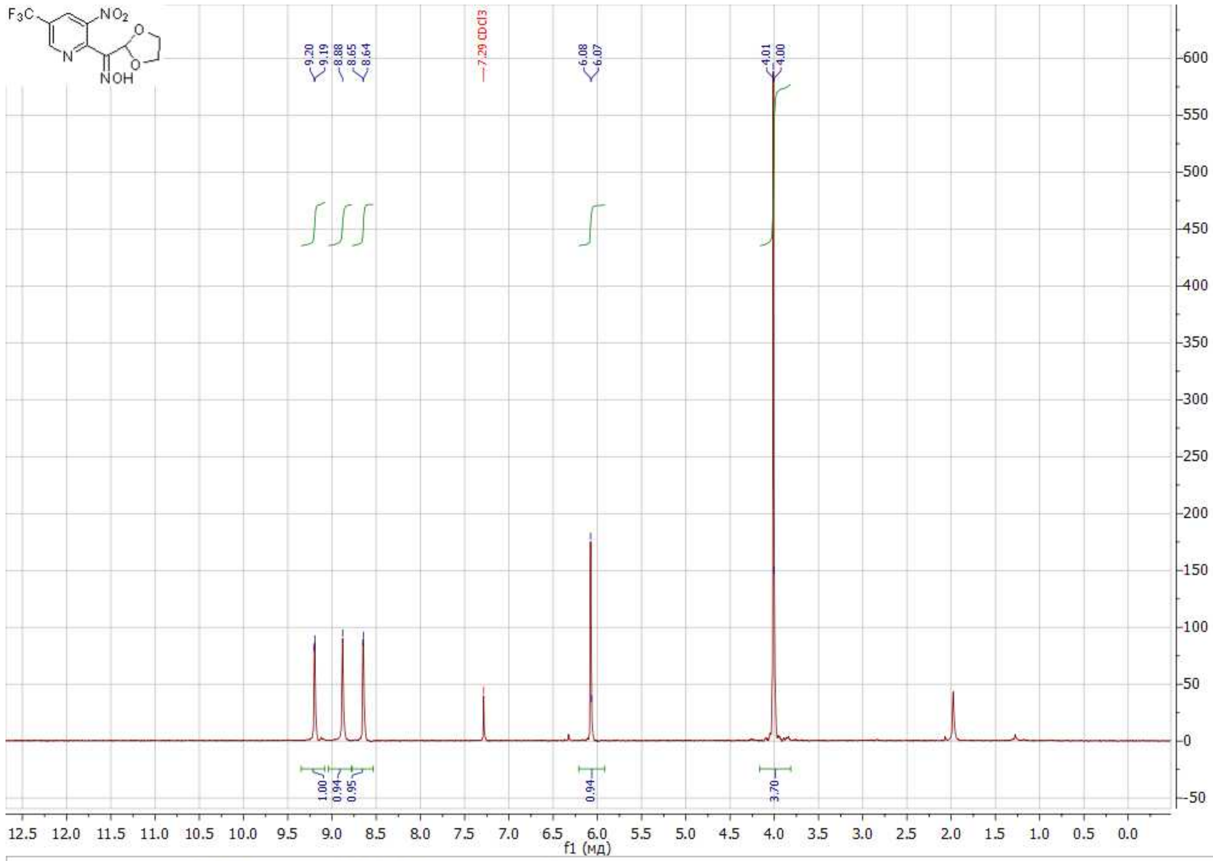

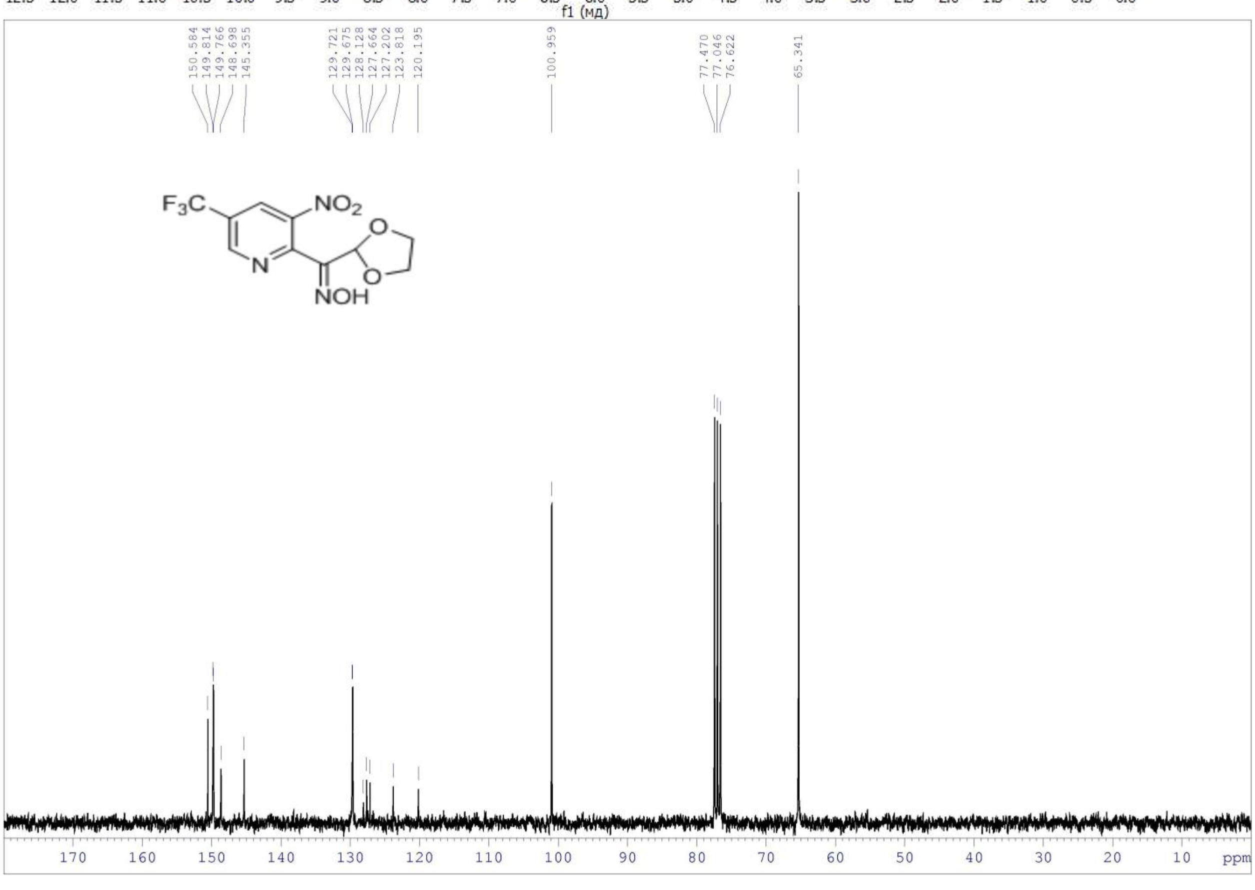

^{1}H and ^{13}C NMR spectra of compound **9c** in DMSO-d_6

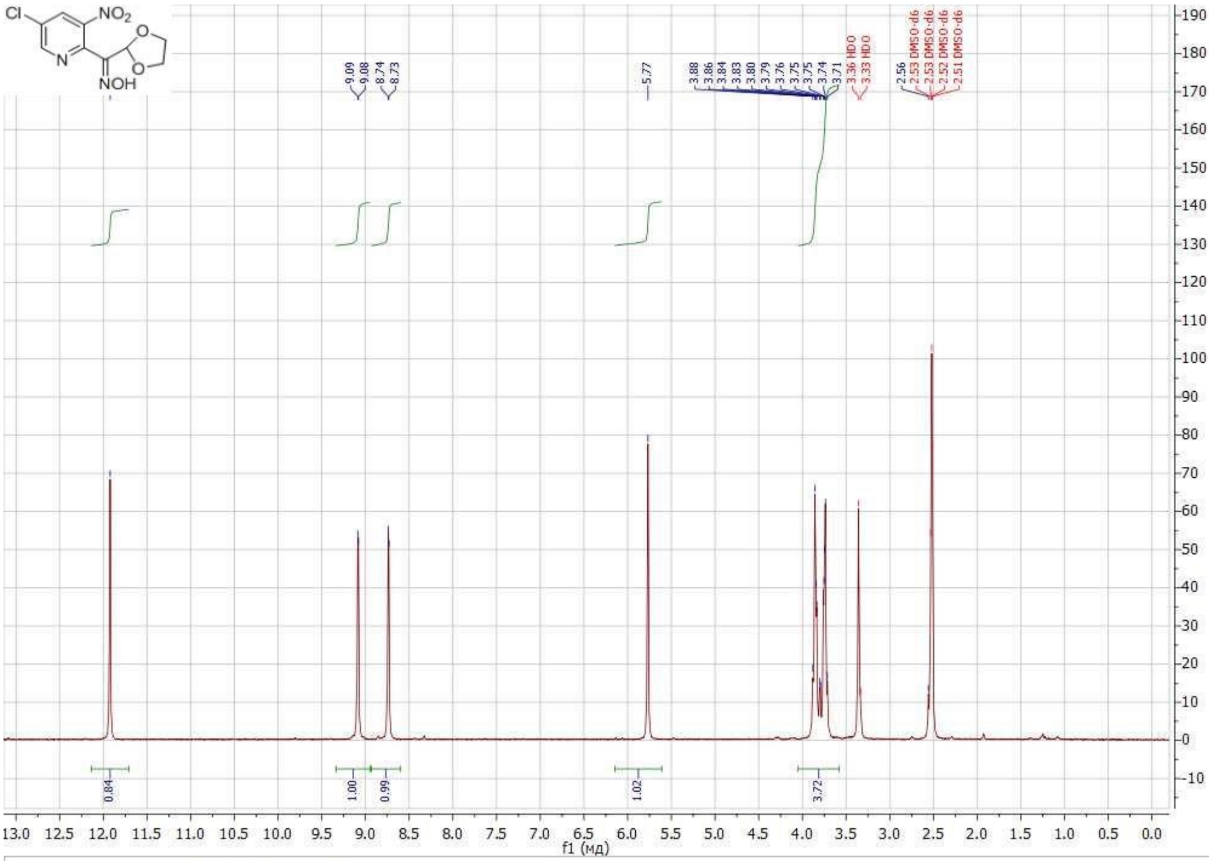

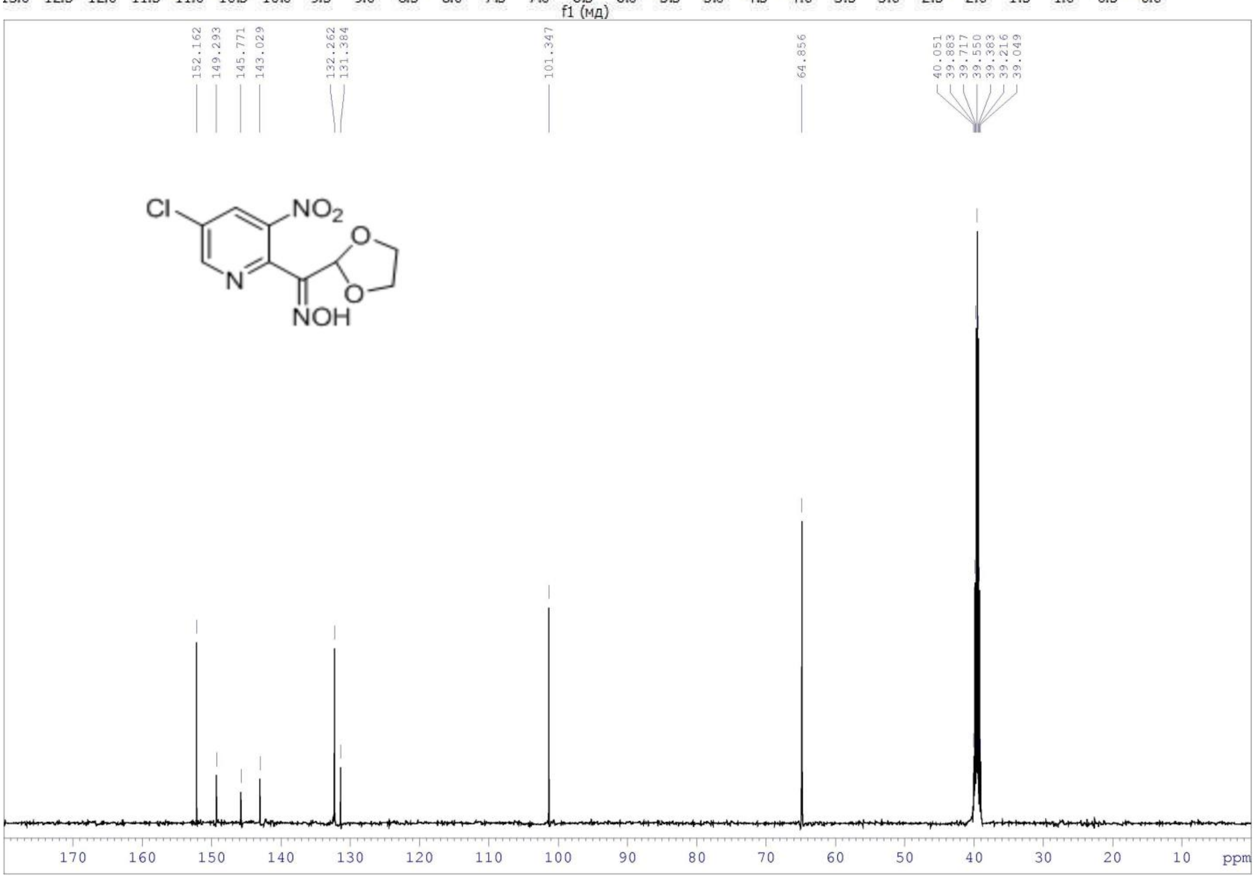

^{1}H and ^{13}C NMR spectra of compound **10a** in DMSO-d_6

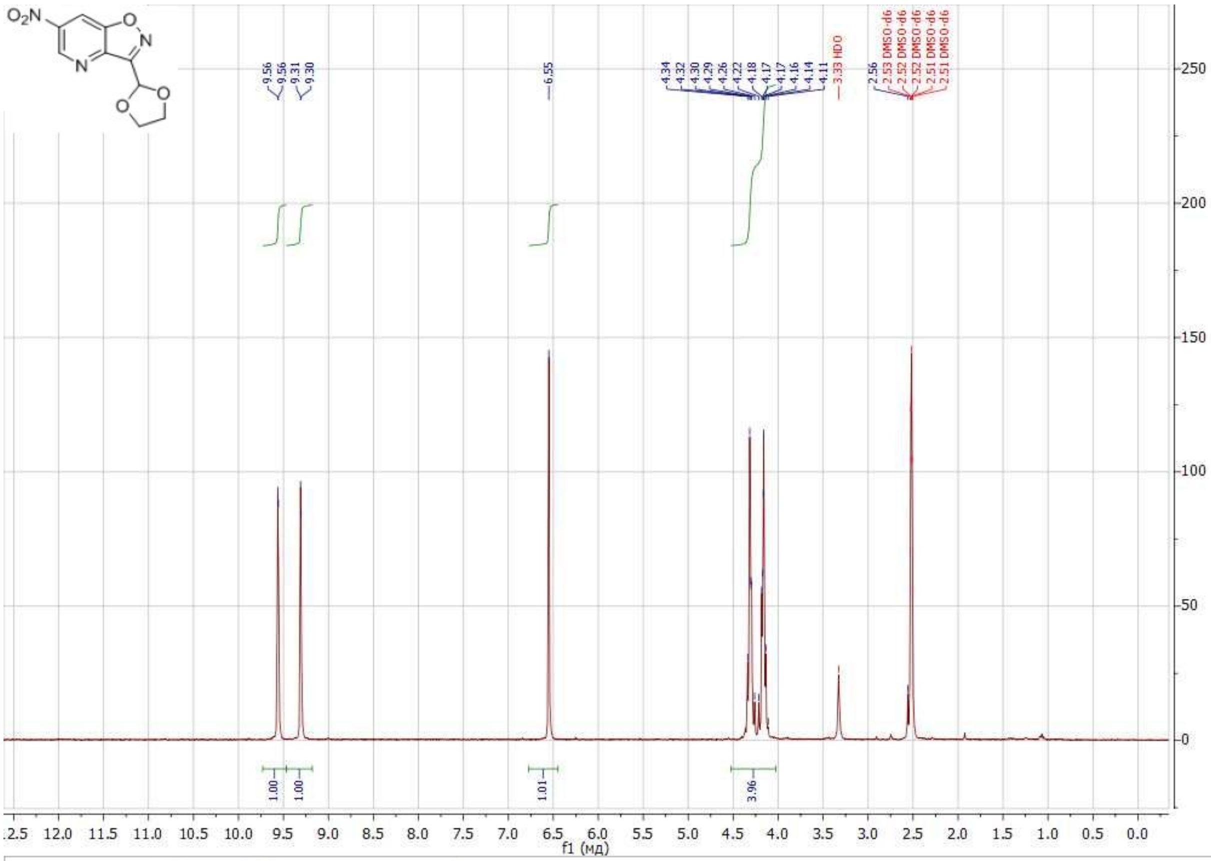

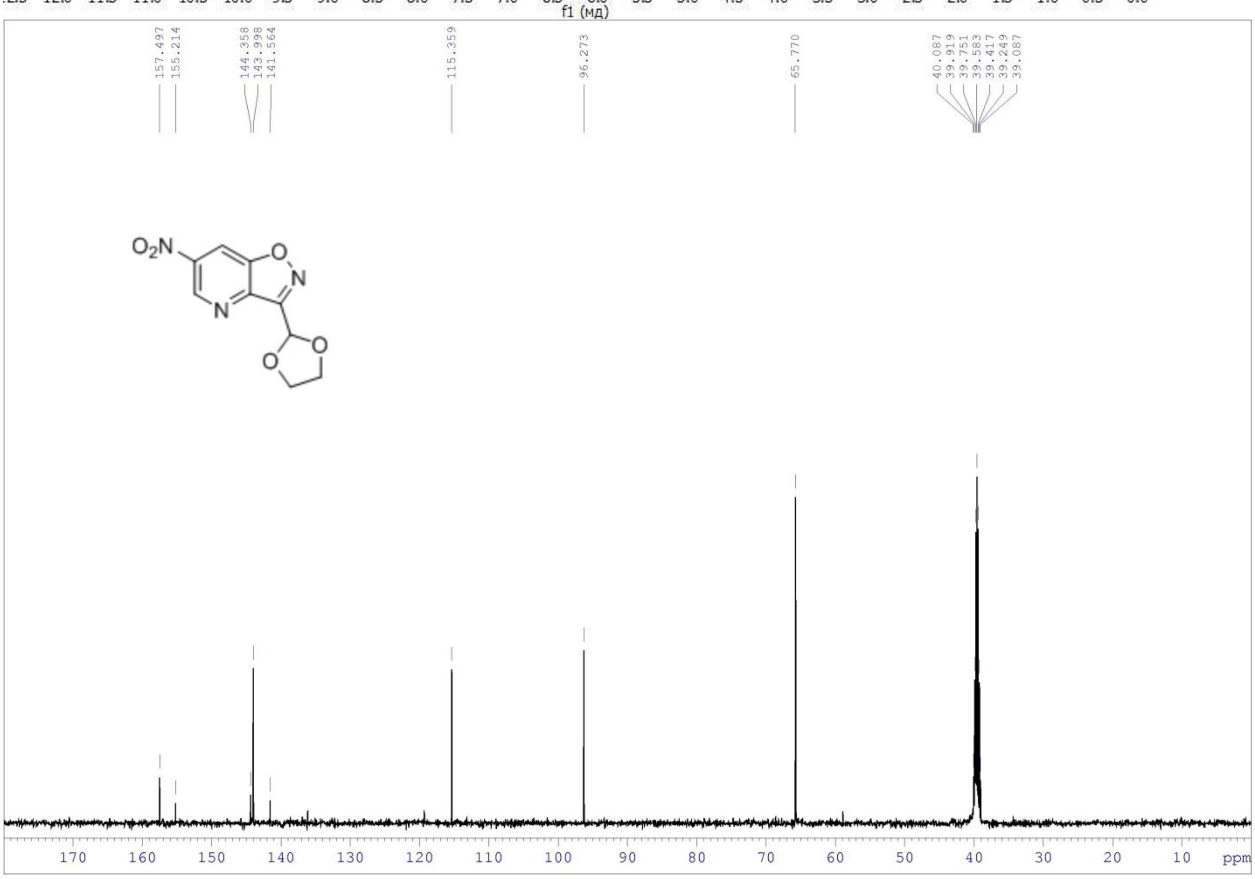

^{1}H and ^{13}C NMR spectra of compound **10b** in $CDCl_3$

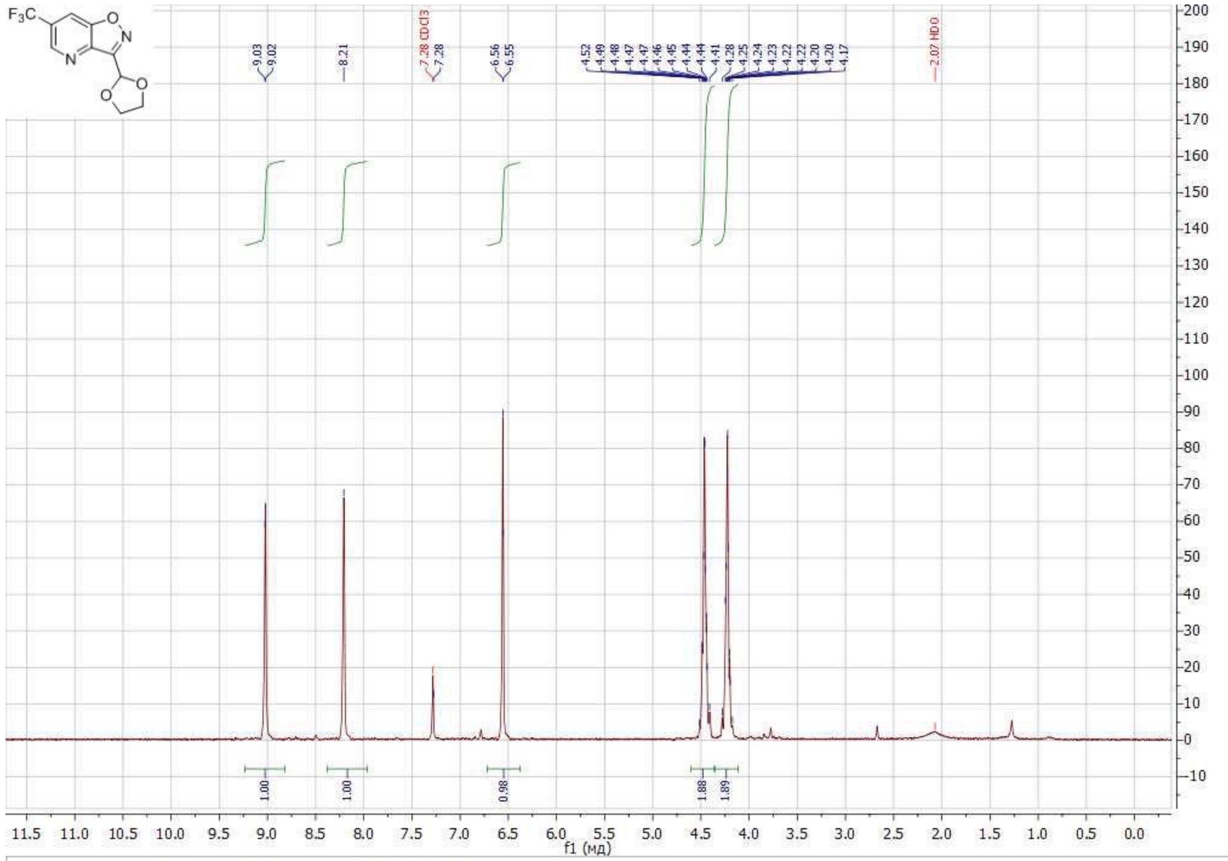

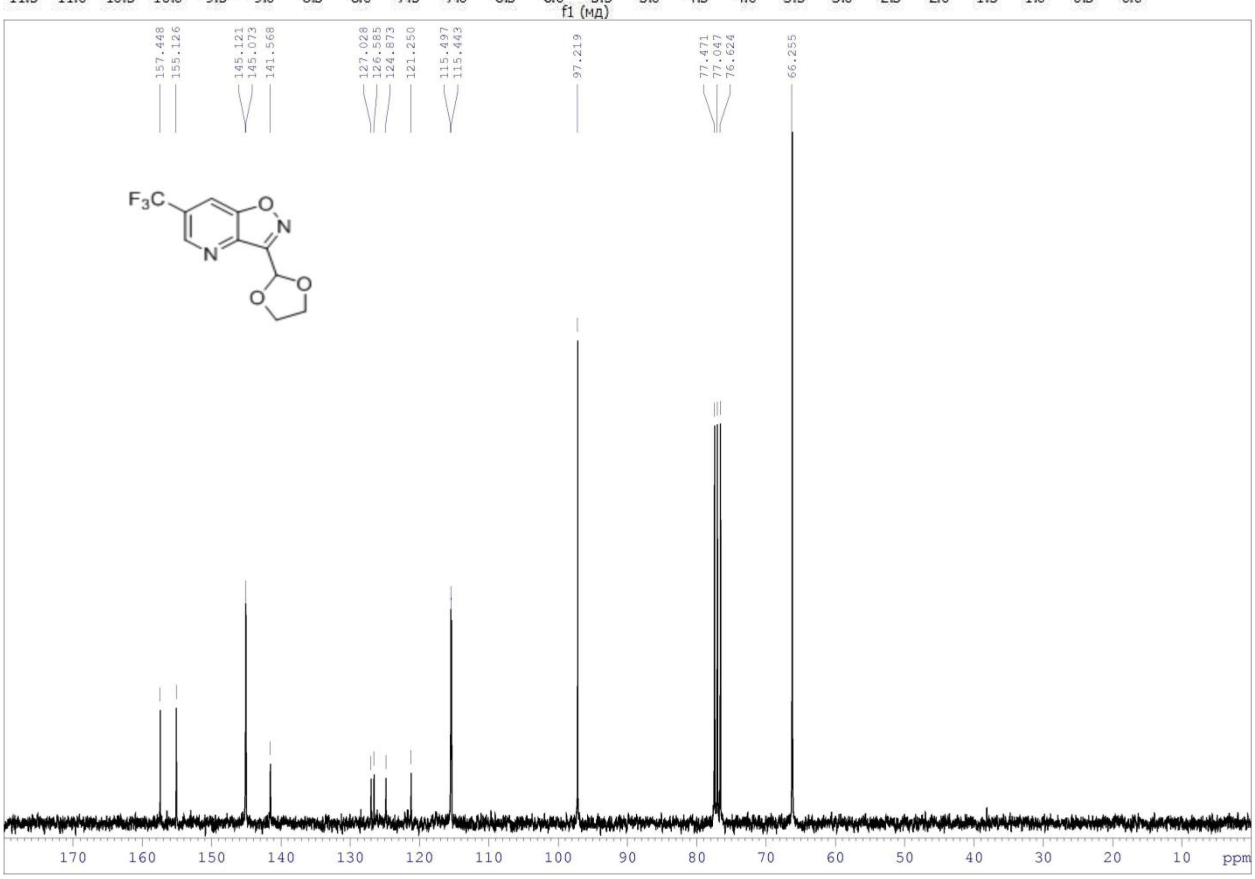

^{1}H and ^{13}C NMR spectra of compound **10c** in $CDCl_3$

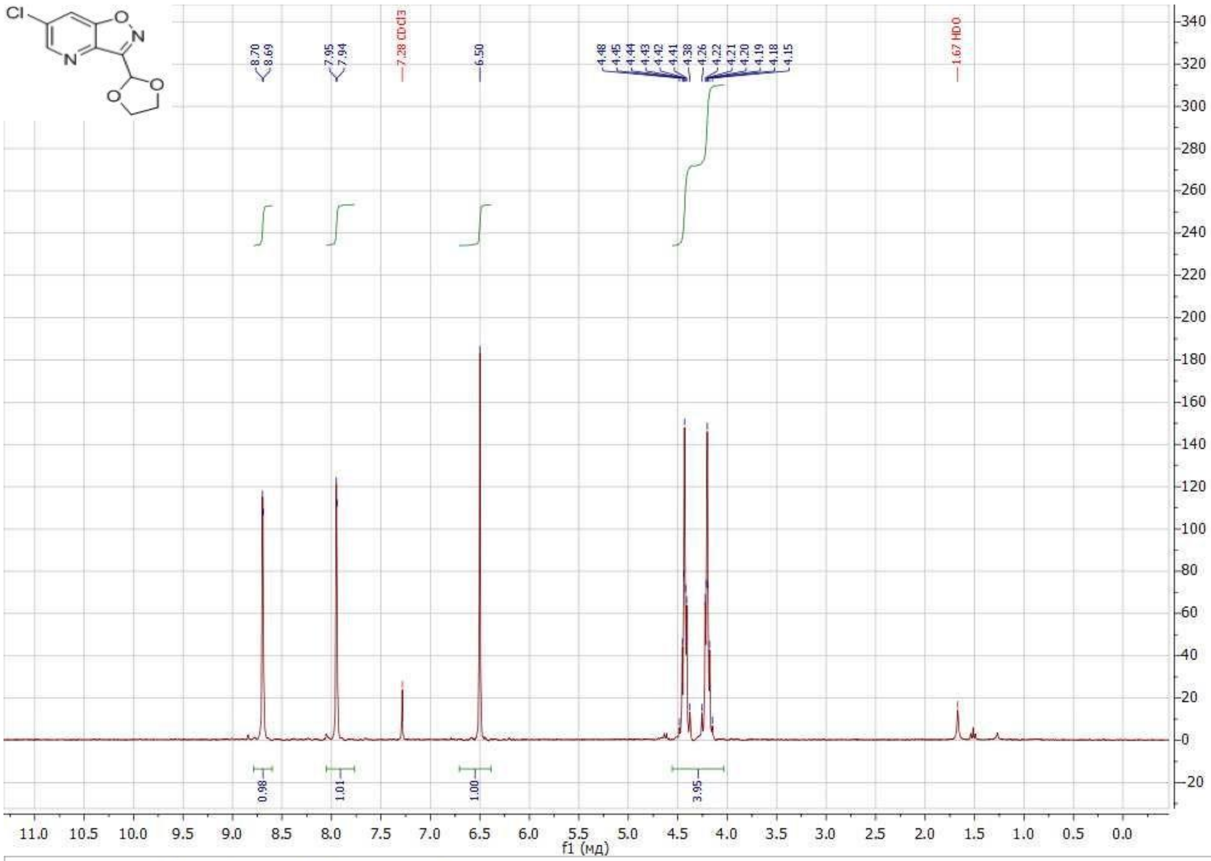

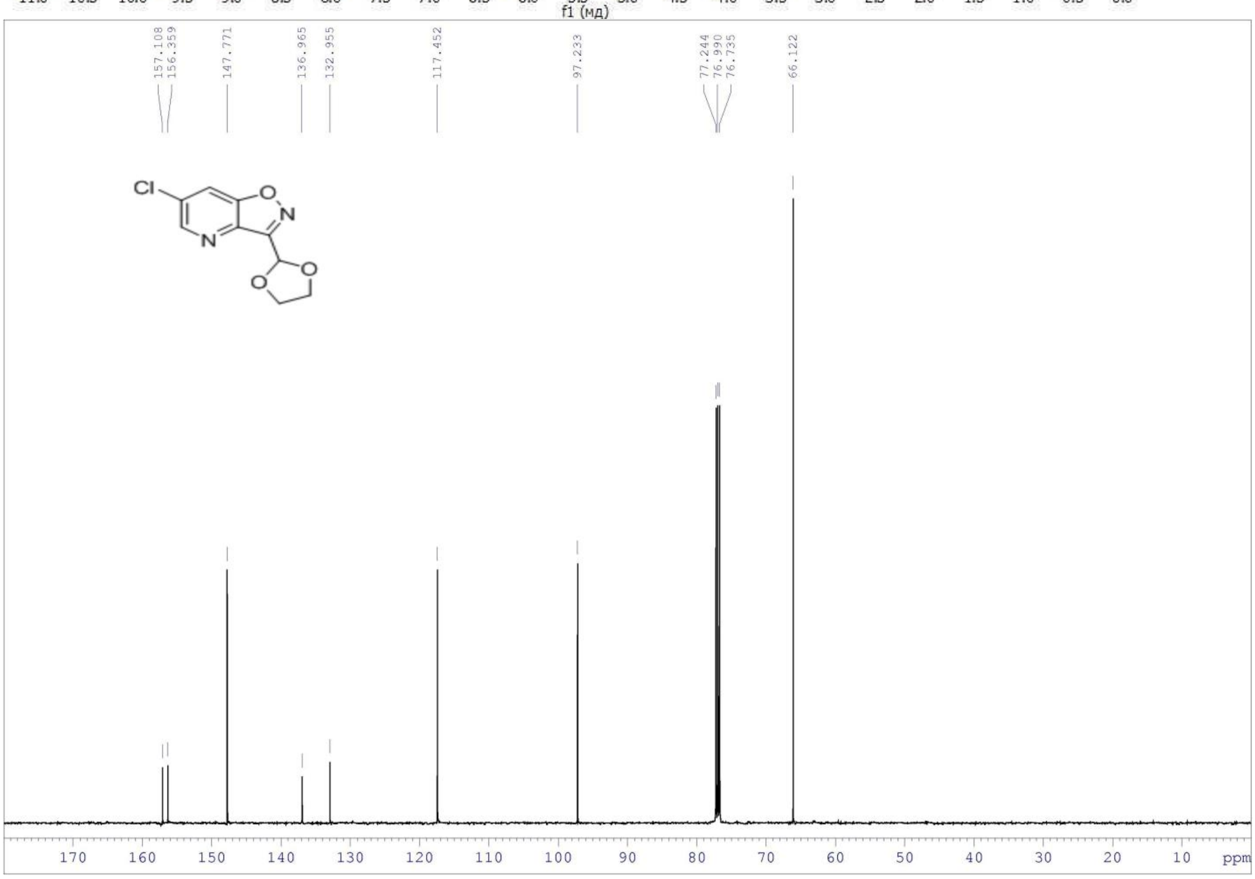

^{1}H NMR spectrum of compounds **12a** and **13a** in DMSO-d_6

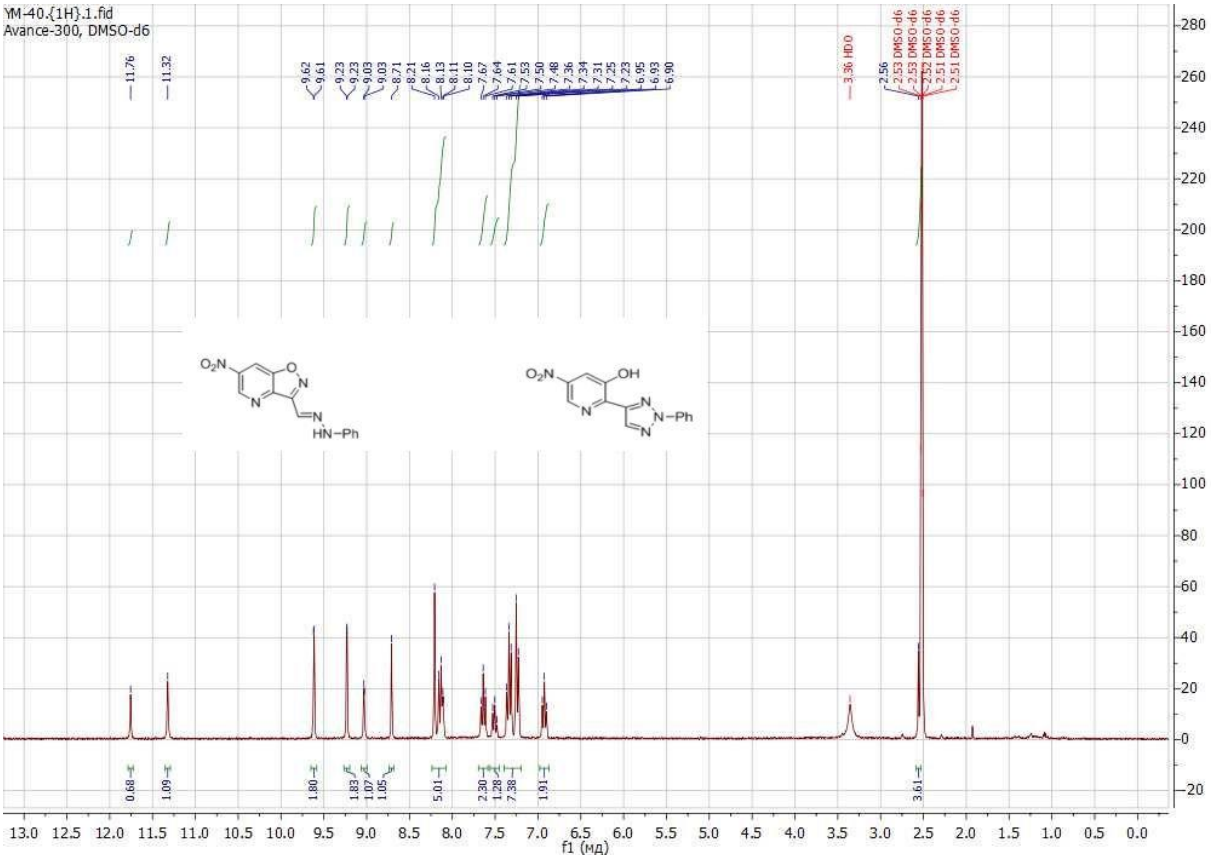

^{1}H NMR spectrum of compounds **12b** in DMSO-d_6

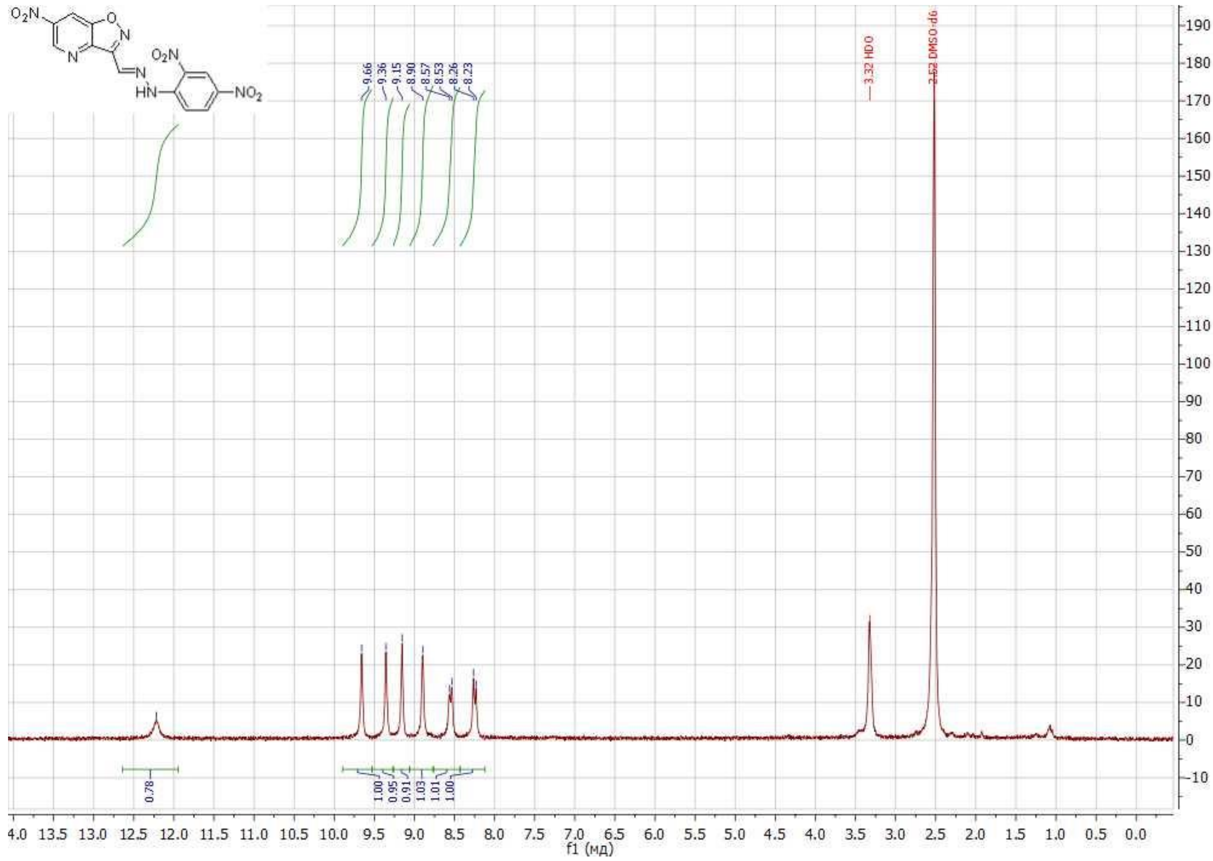

^{1}H and ^{13}C NMR spectra of compound **12c** in DMSO-d_6

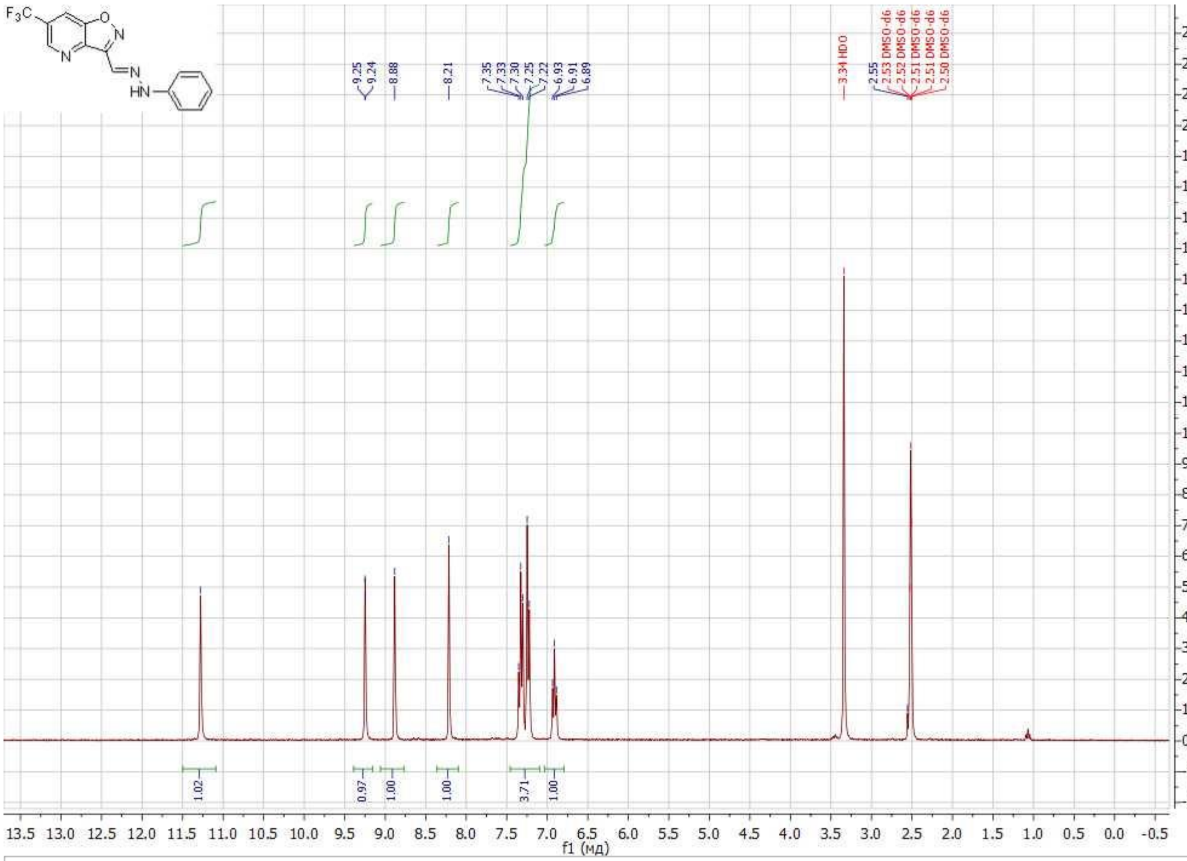

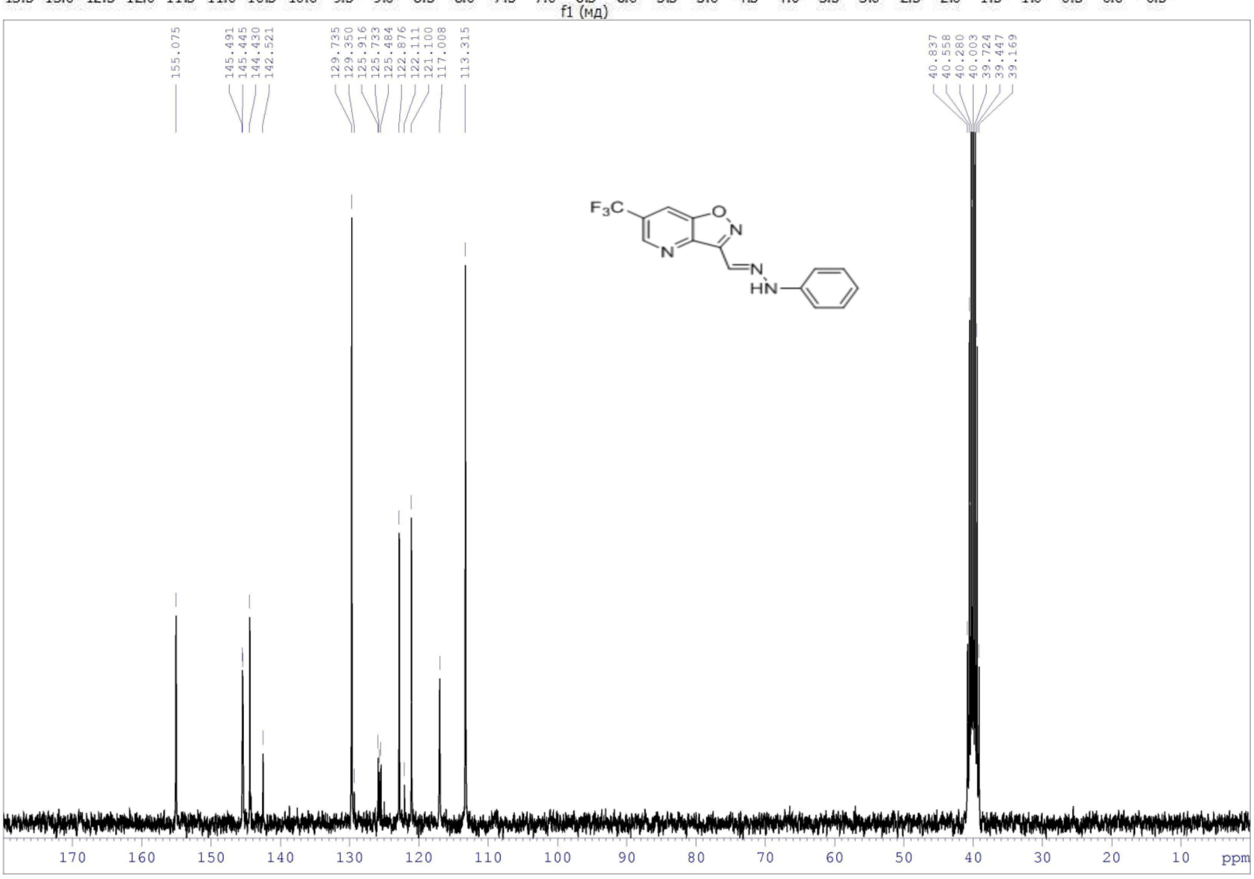

^1H and ^{13}C NMR spectra of compound **12d** in DMSO-d_6

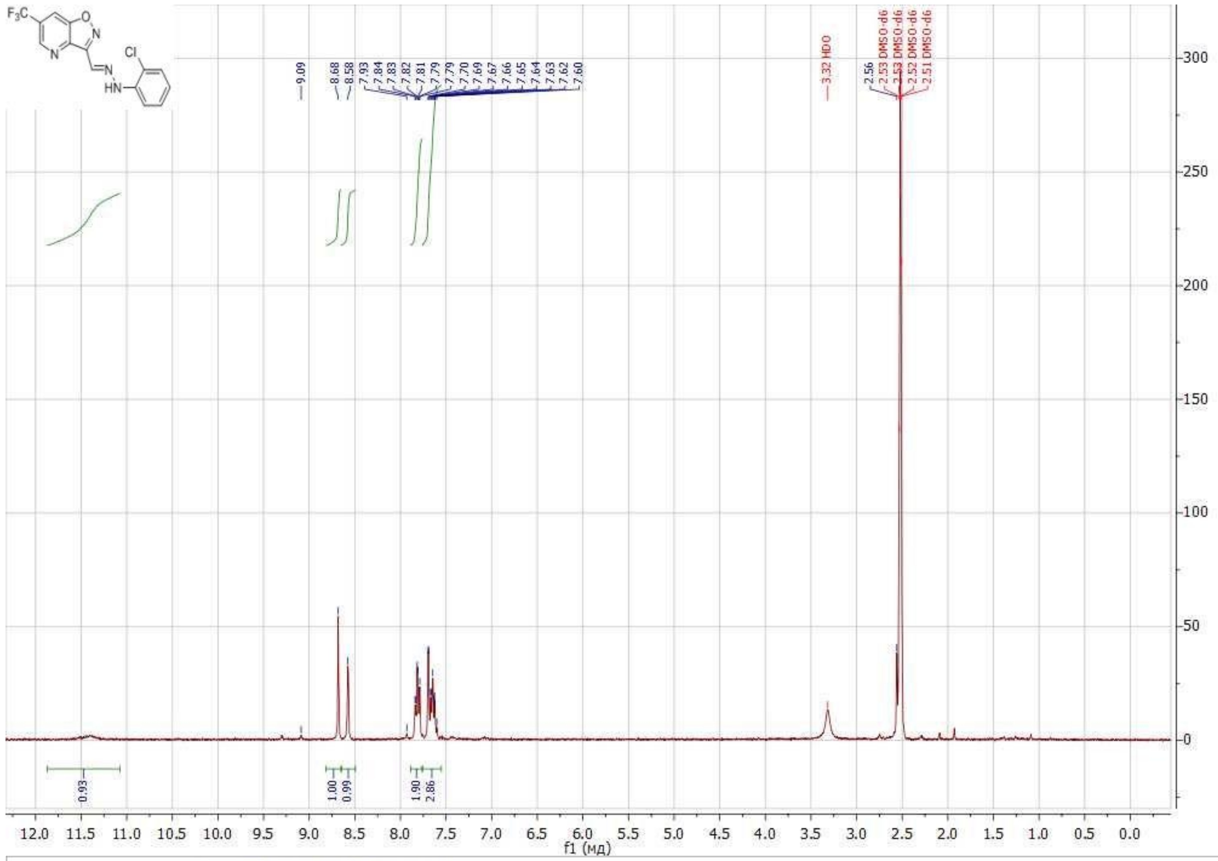

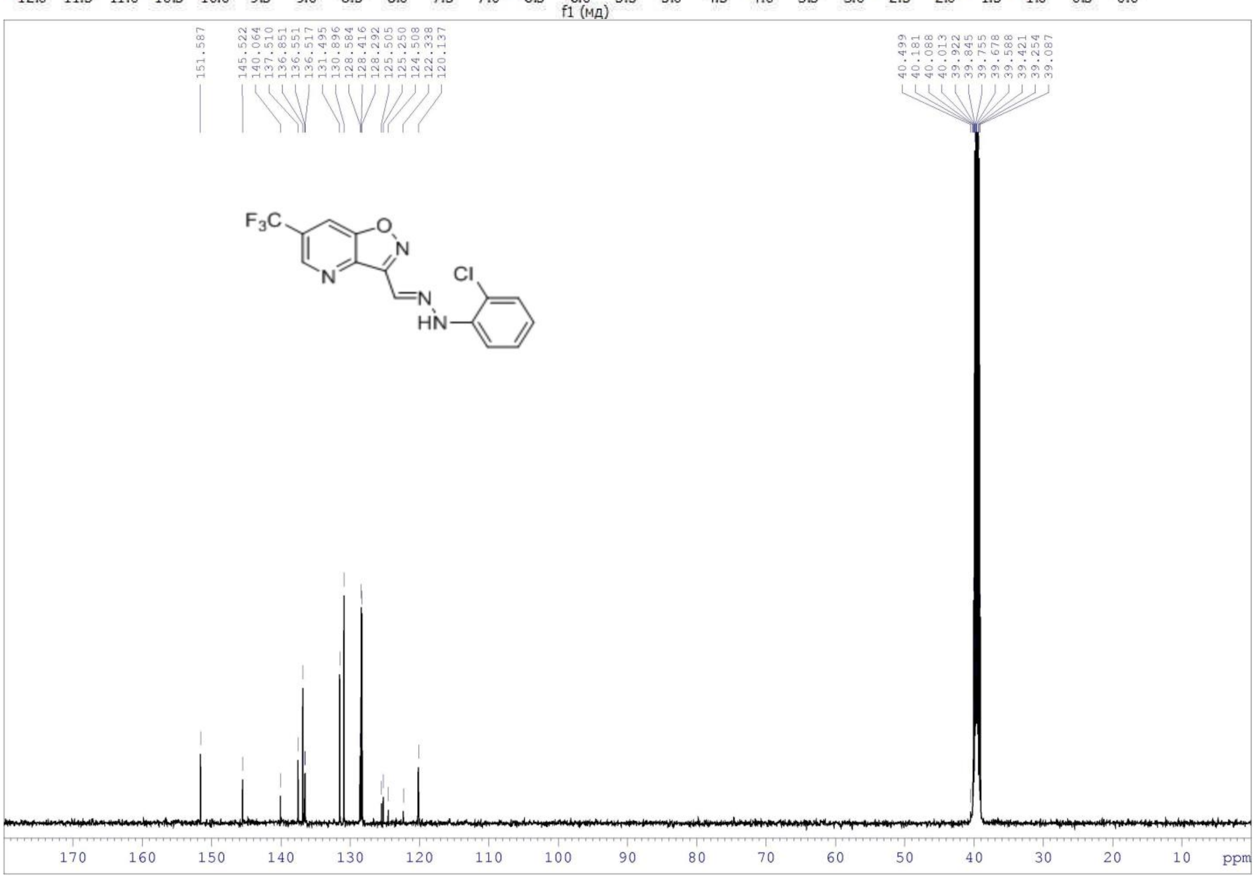

^{1}H NMR spectrum of compound **12e** in DMSO-d_6

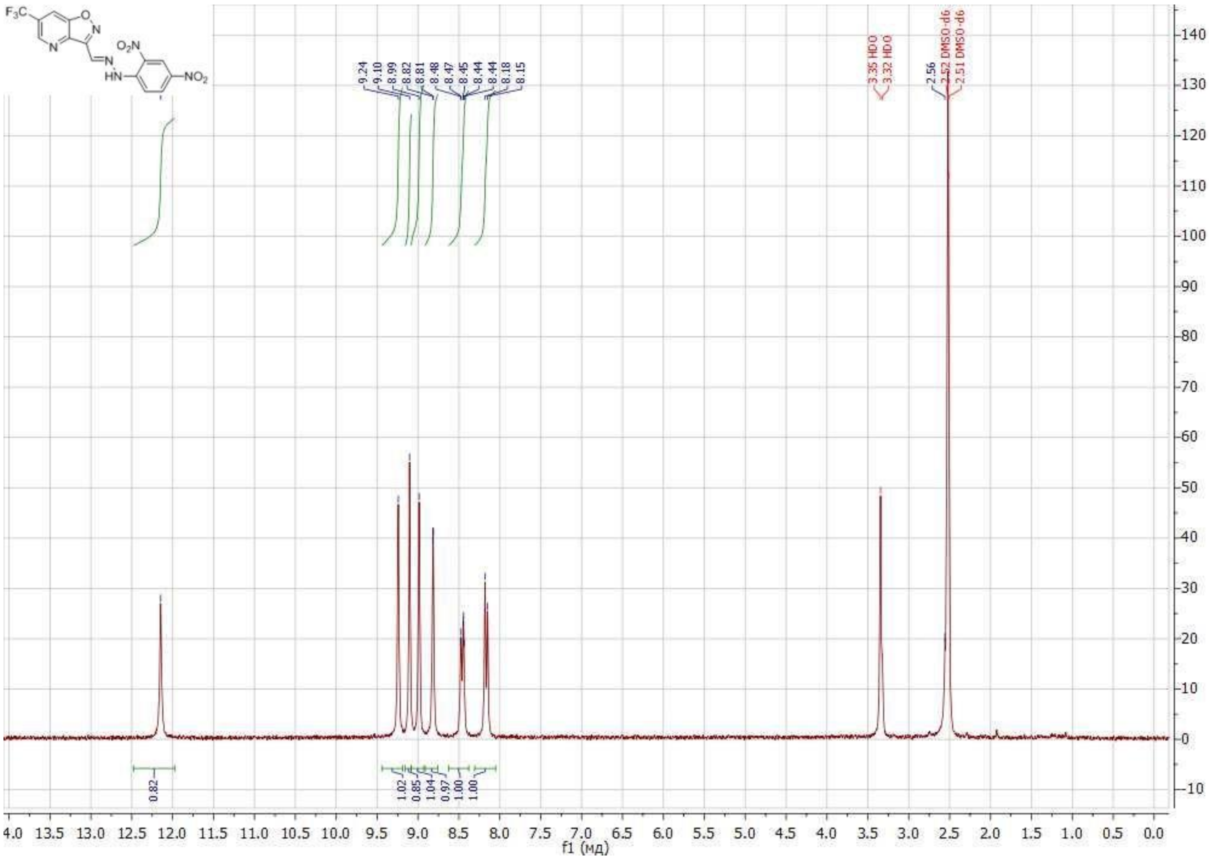

^{1}H and ^{13}C NMR spectra of compound **12f** in DMSO-d_6

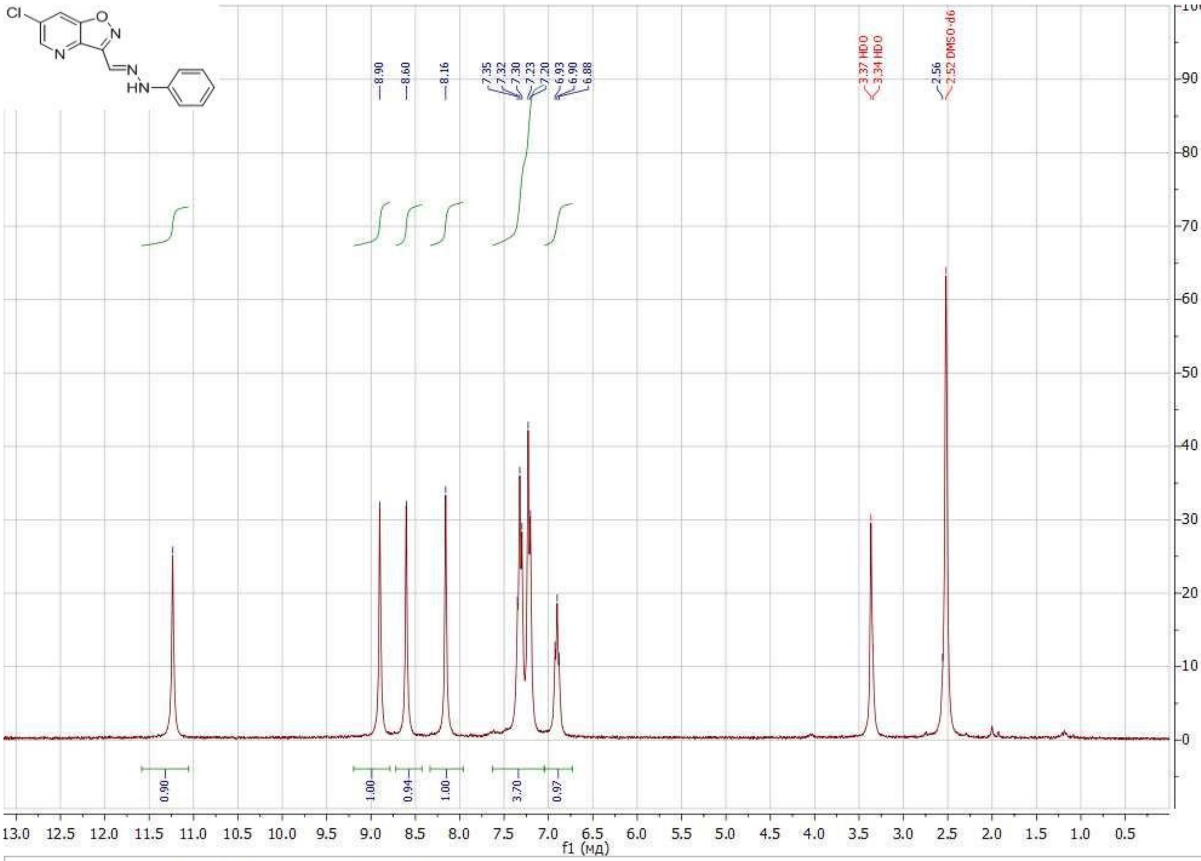

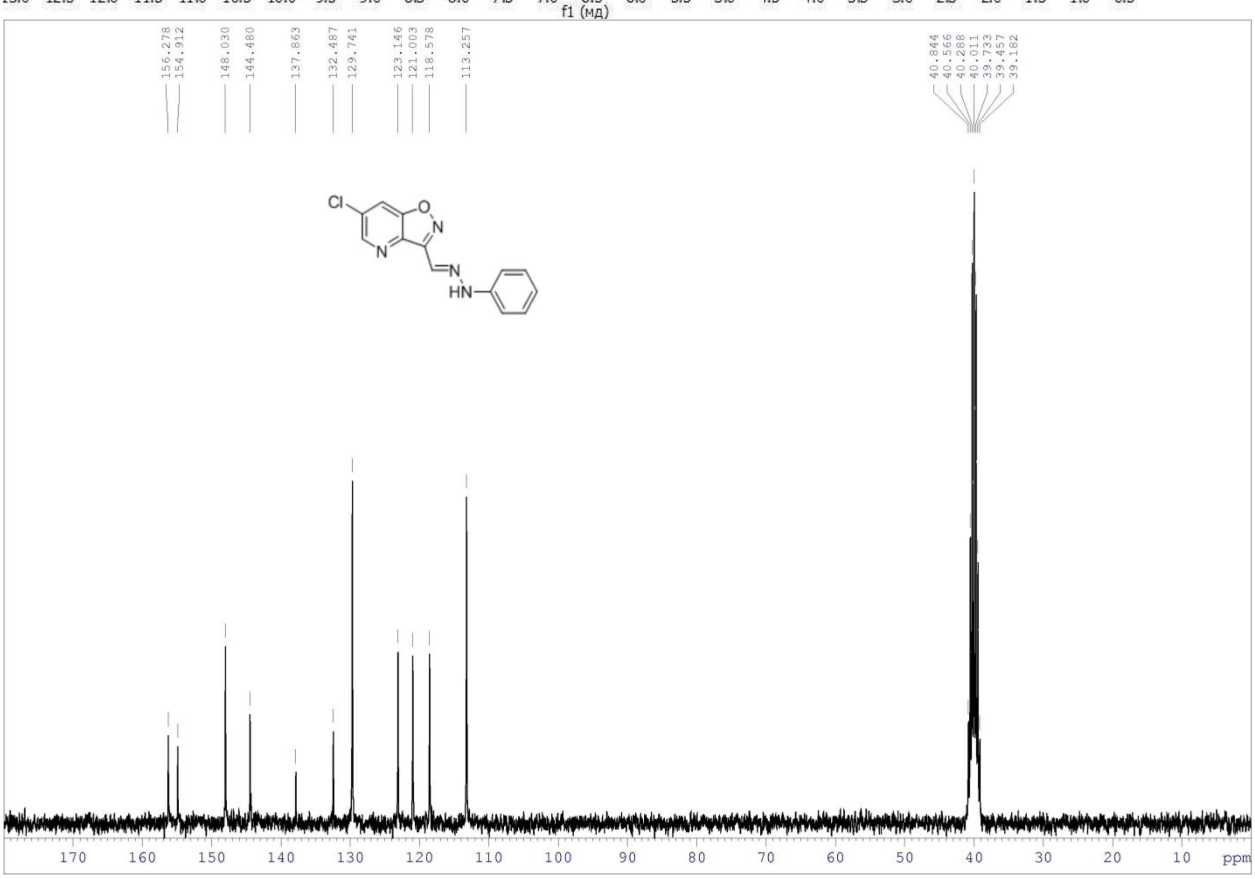

^{1}H and ^{13}C NMR spectra of compound **12g** in DMSO-d_6

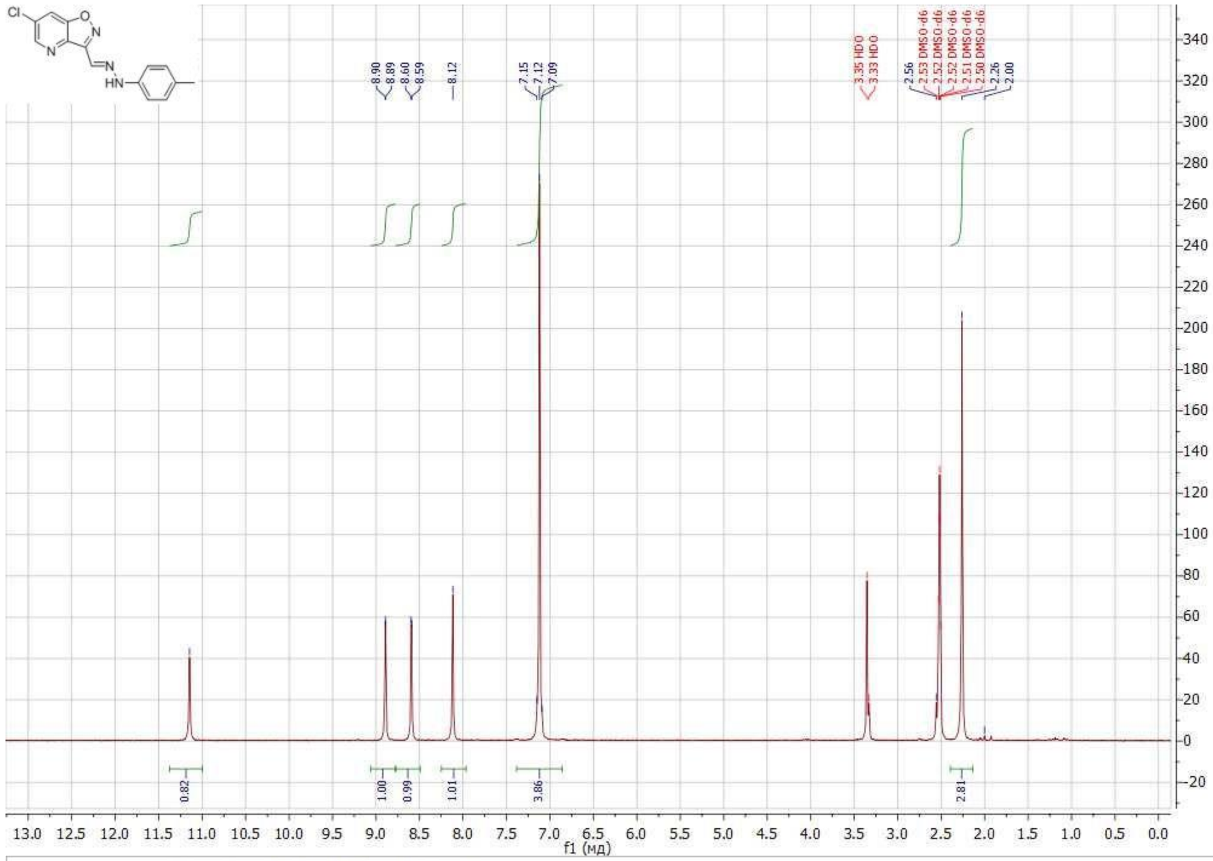

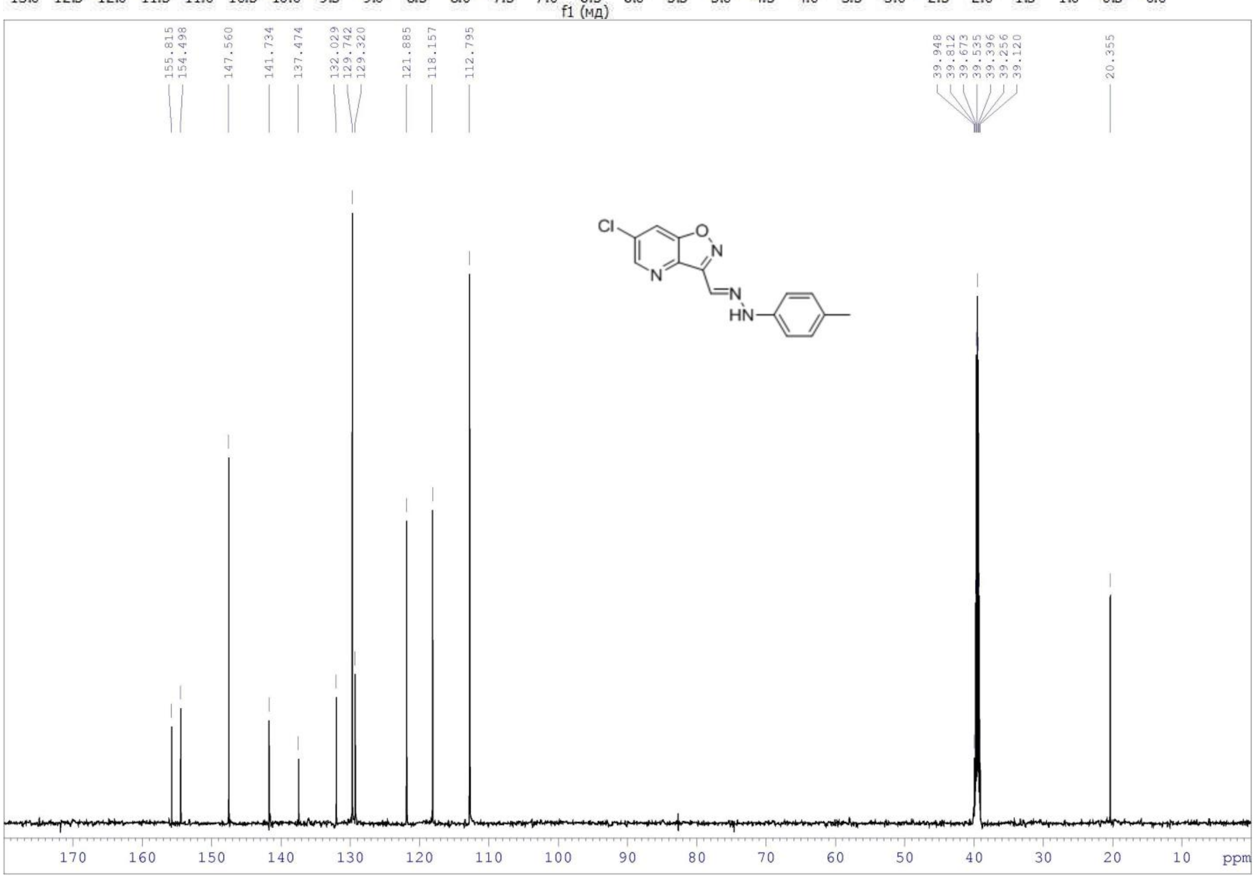

^{1}H NMR spectrum of compound **12h** in DMSO-d_6

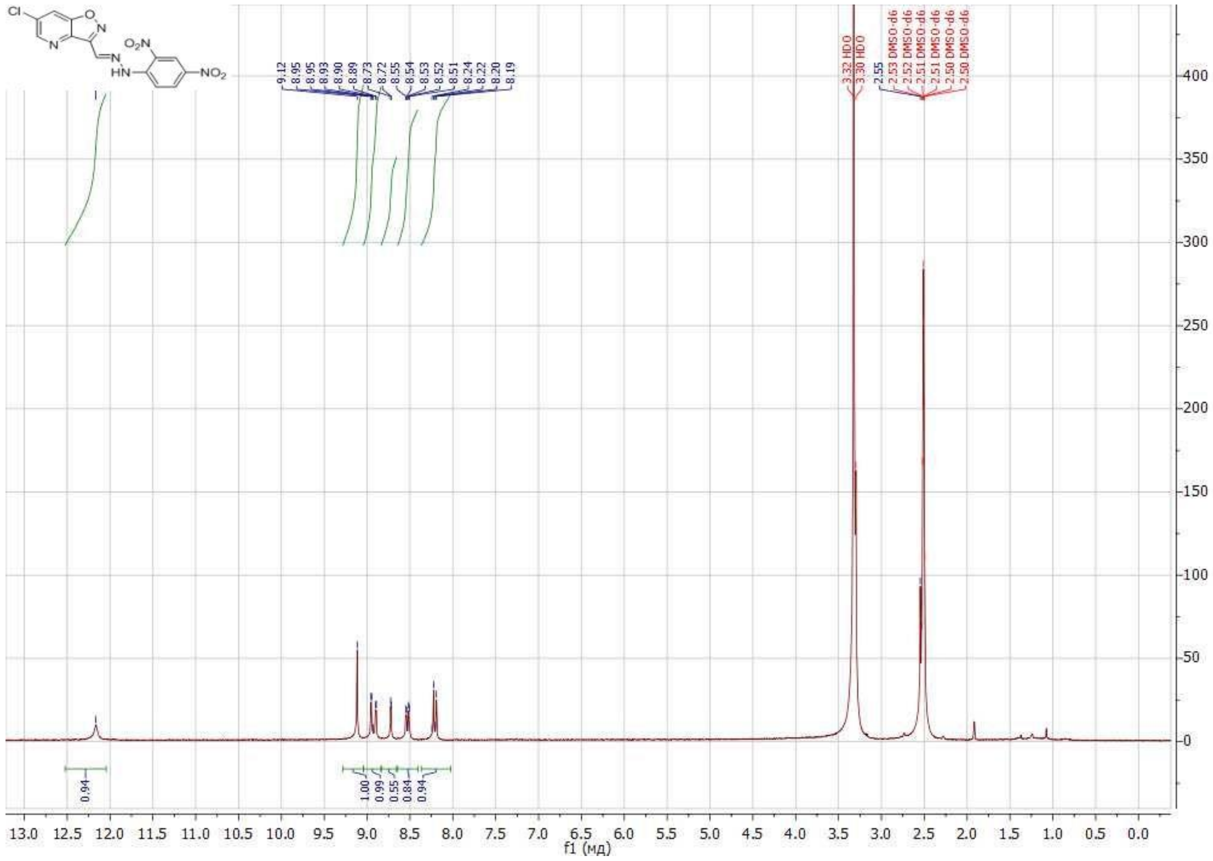

^{1}H and ^{13}C NMR spectra of compound **13a** in DMSO-d_6

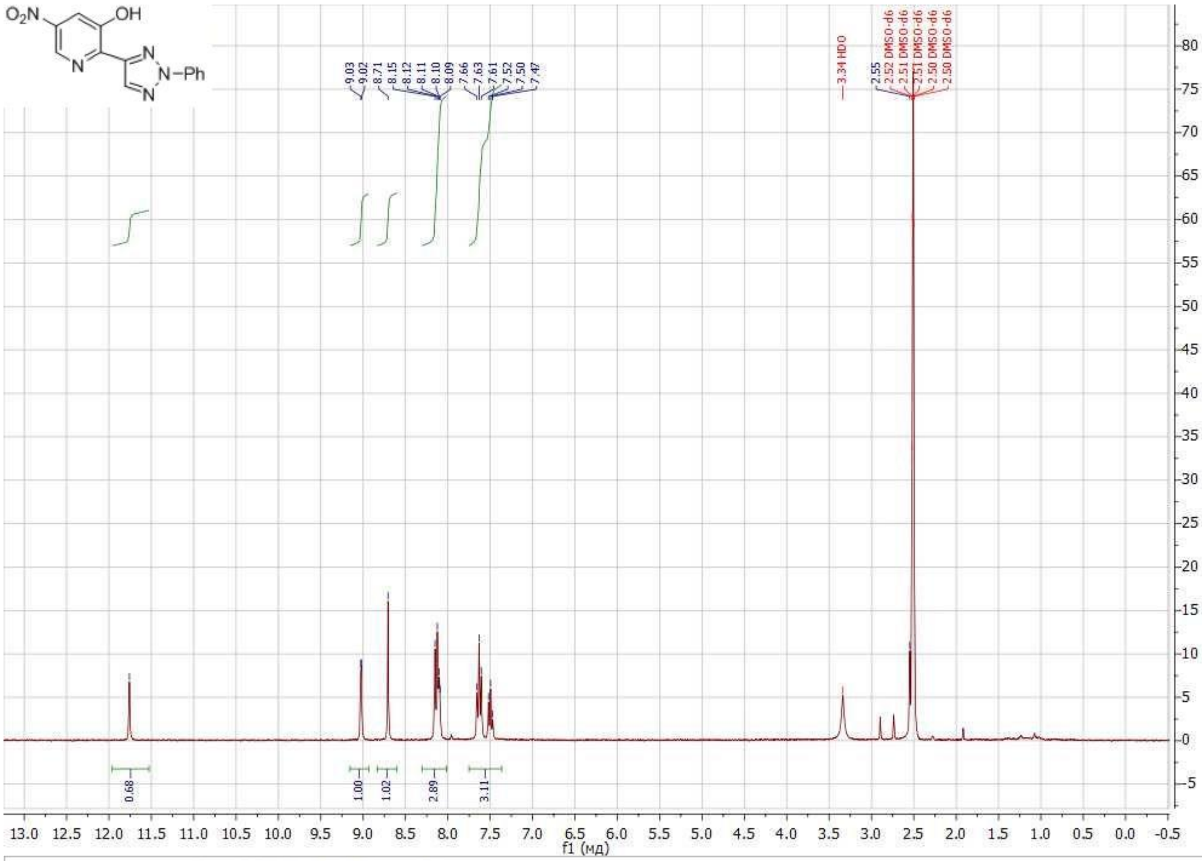

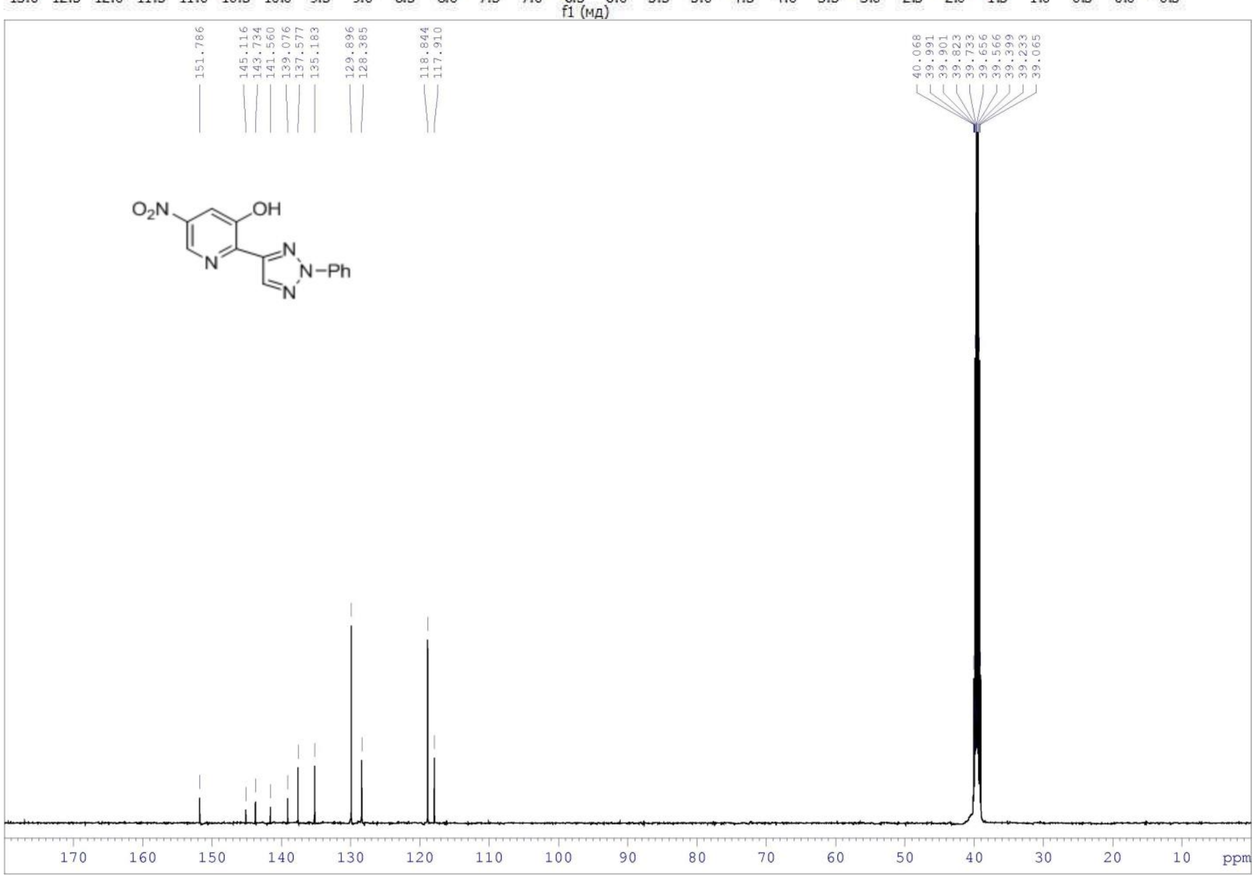

^{1}H and ^{13}C NMR spectra of compound **13c** in $CDCl_3$

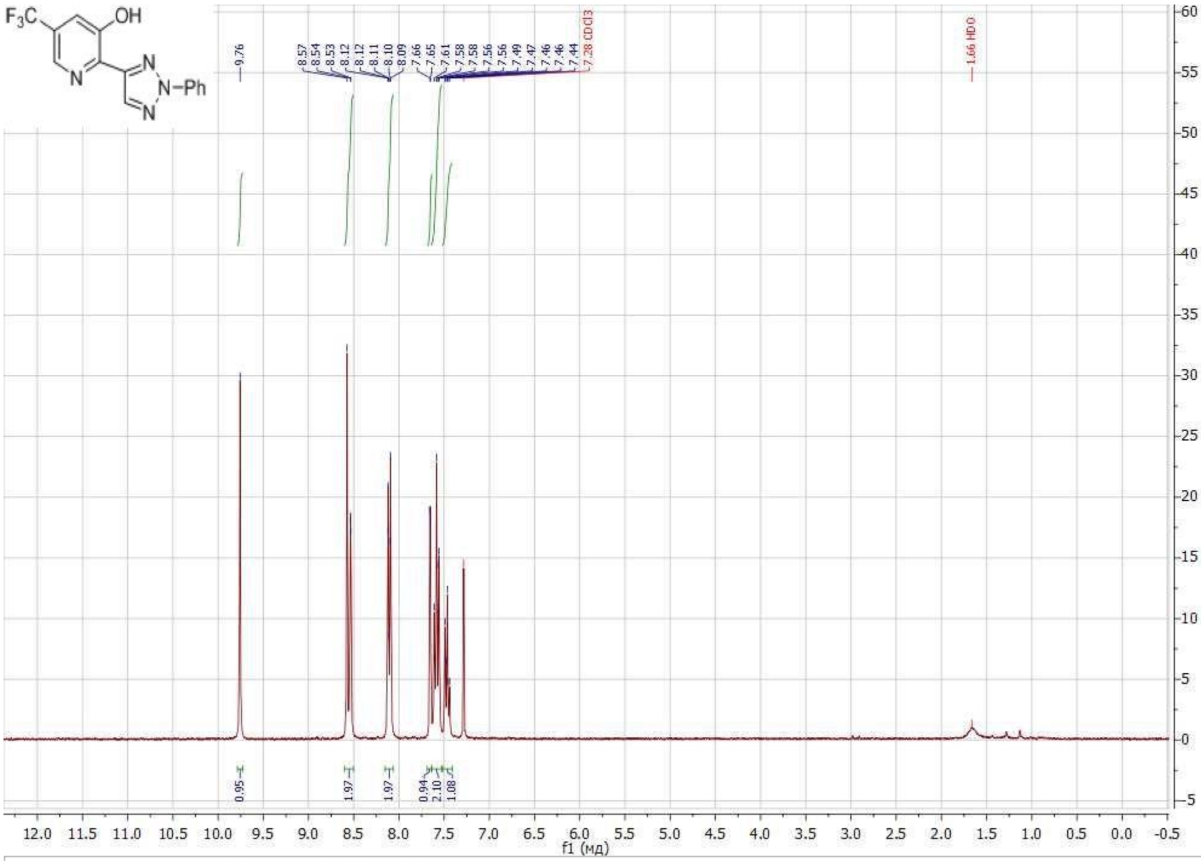

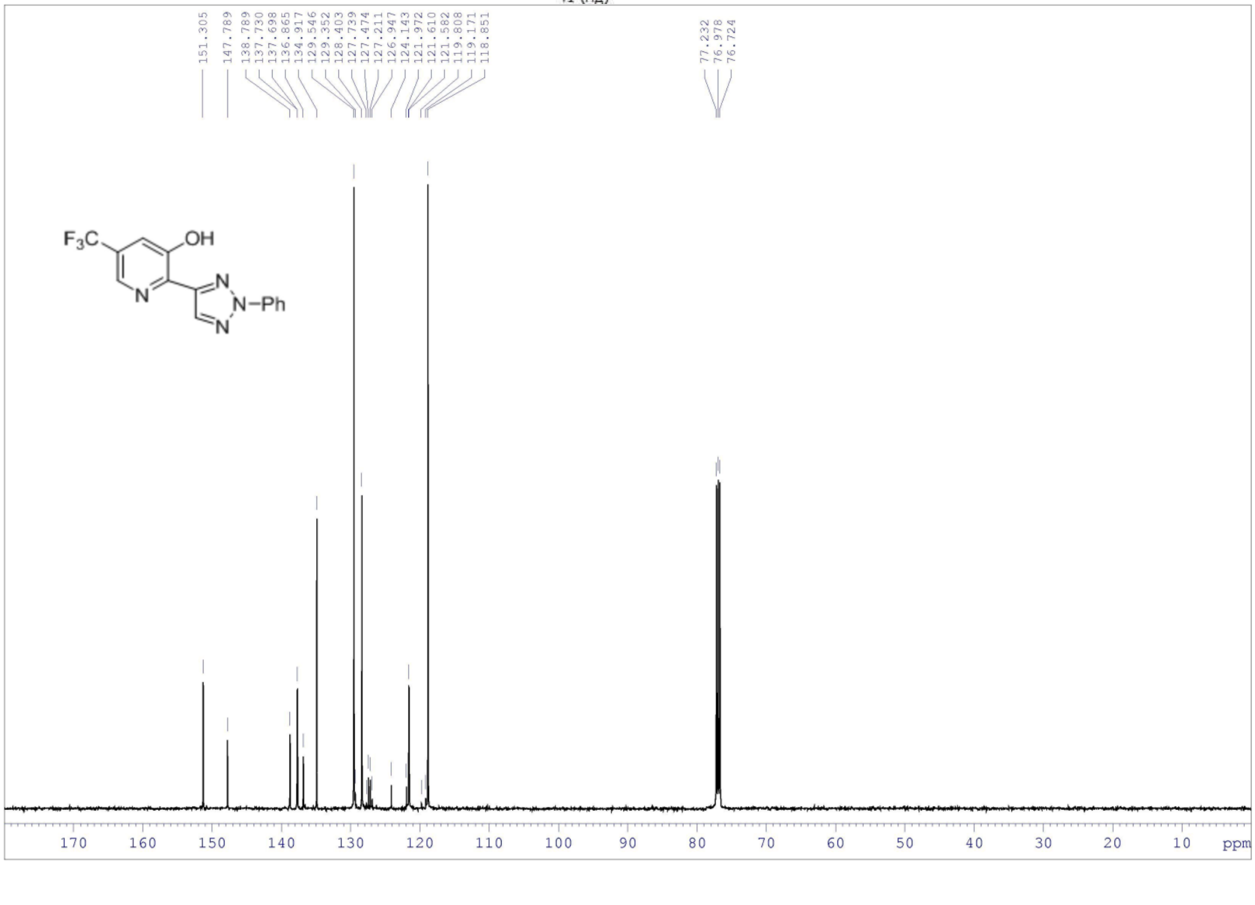

^{1}H and ^{13}C NMR spectra of compound **13d** in $CDCl_3$

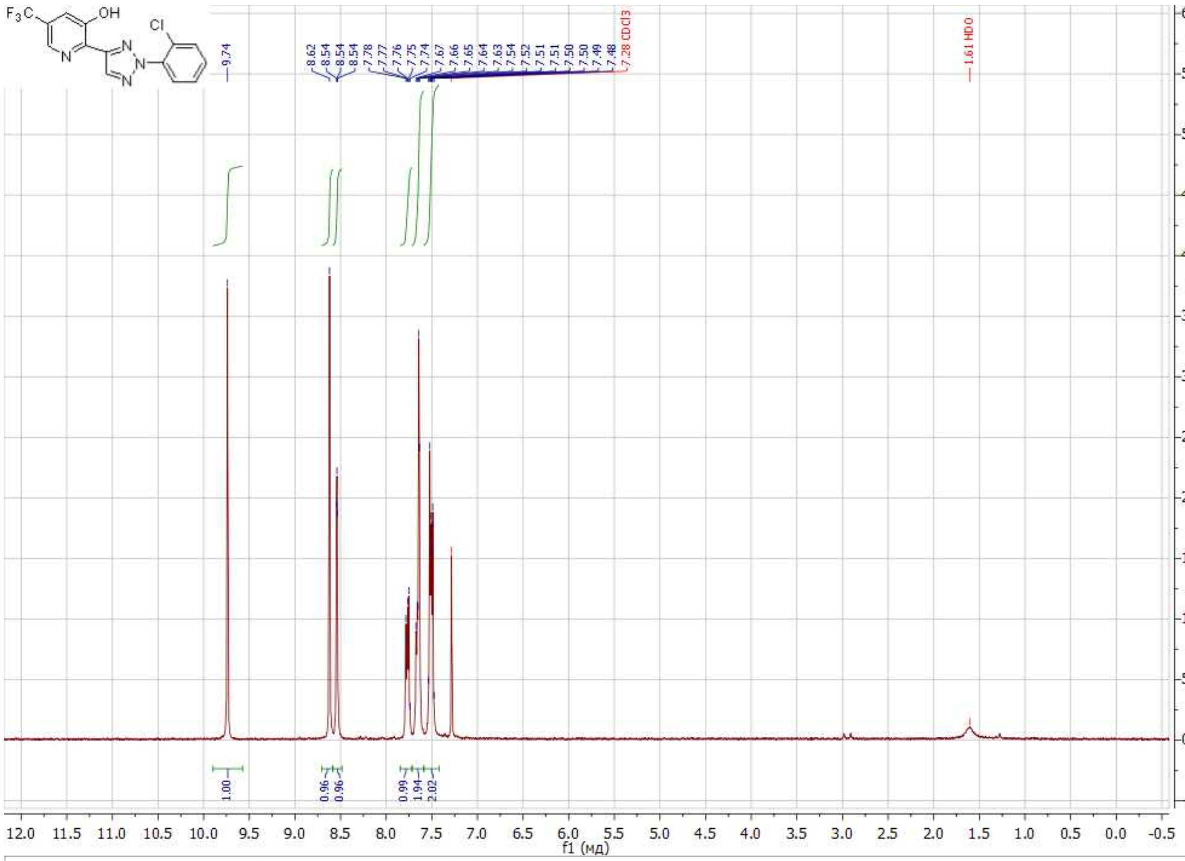

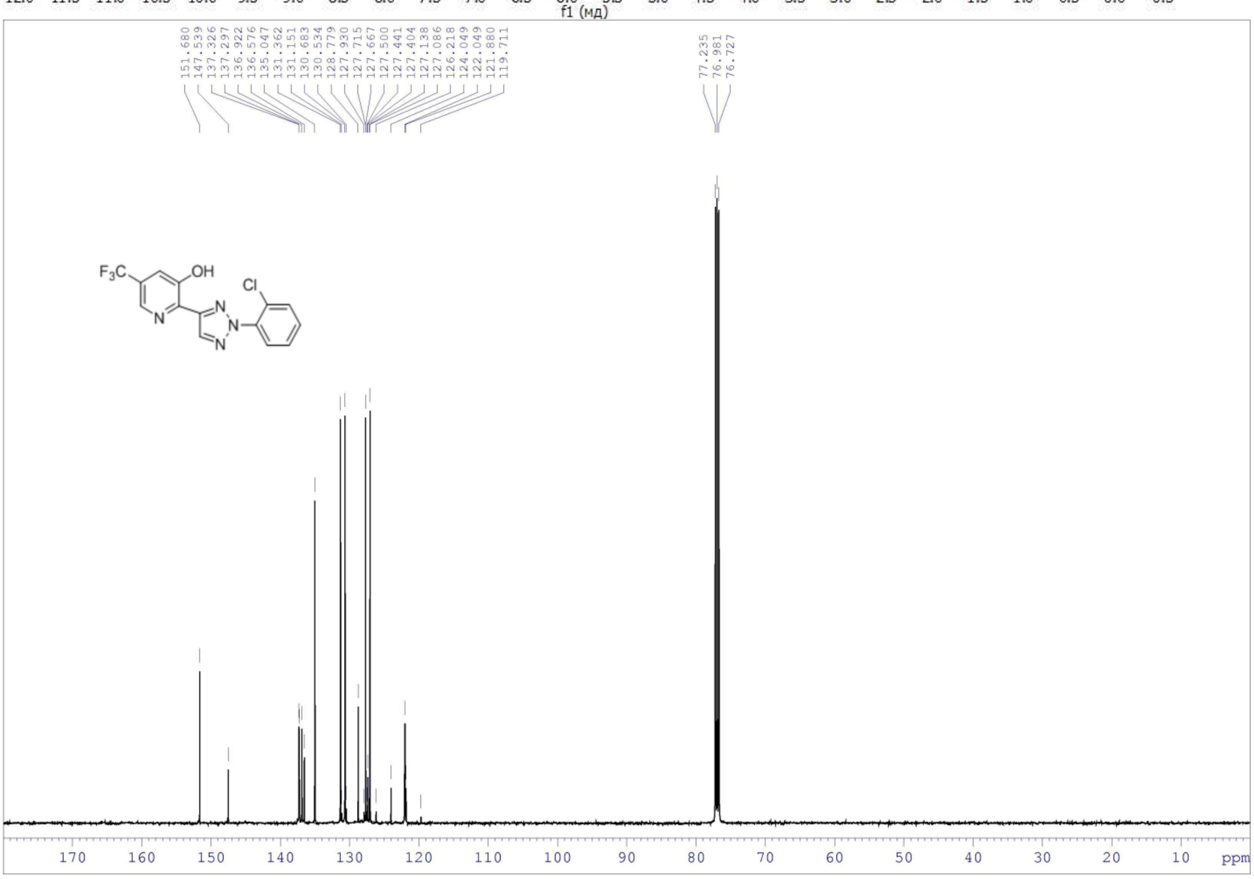

^{1}H and ^{13}C NMR spectra of compound **13f** in DMSO-d_6

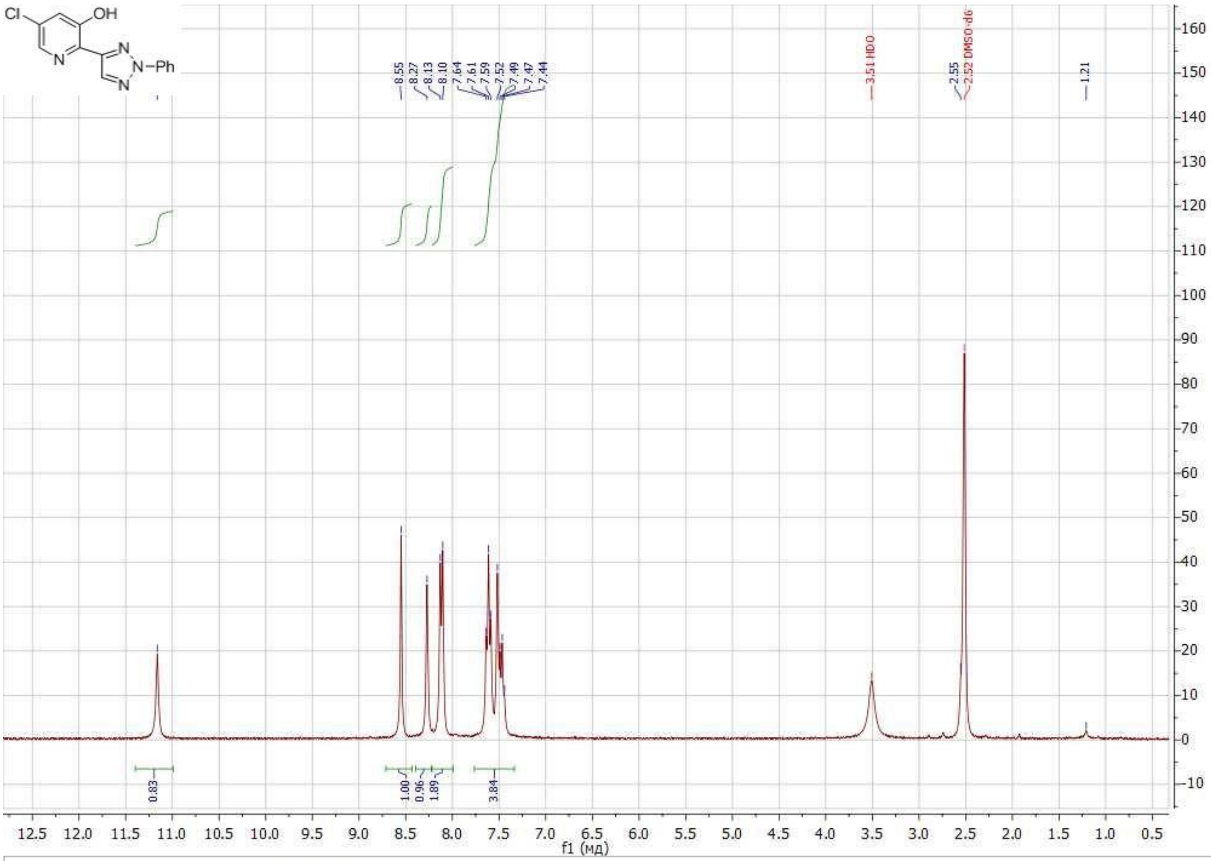

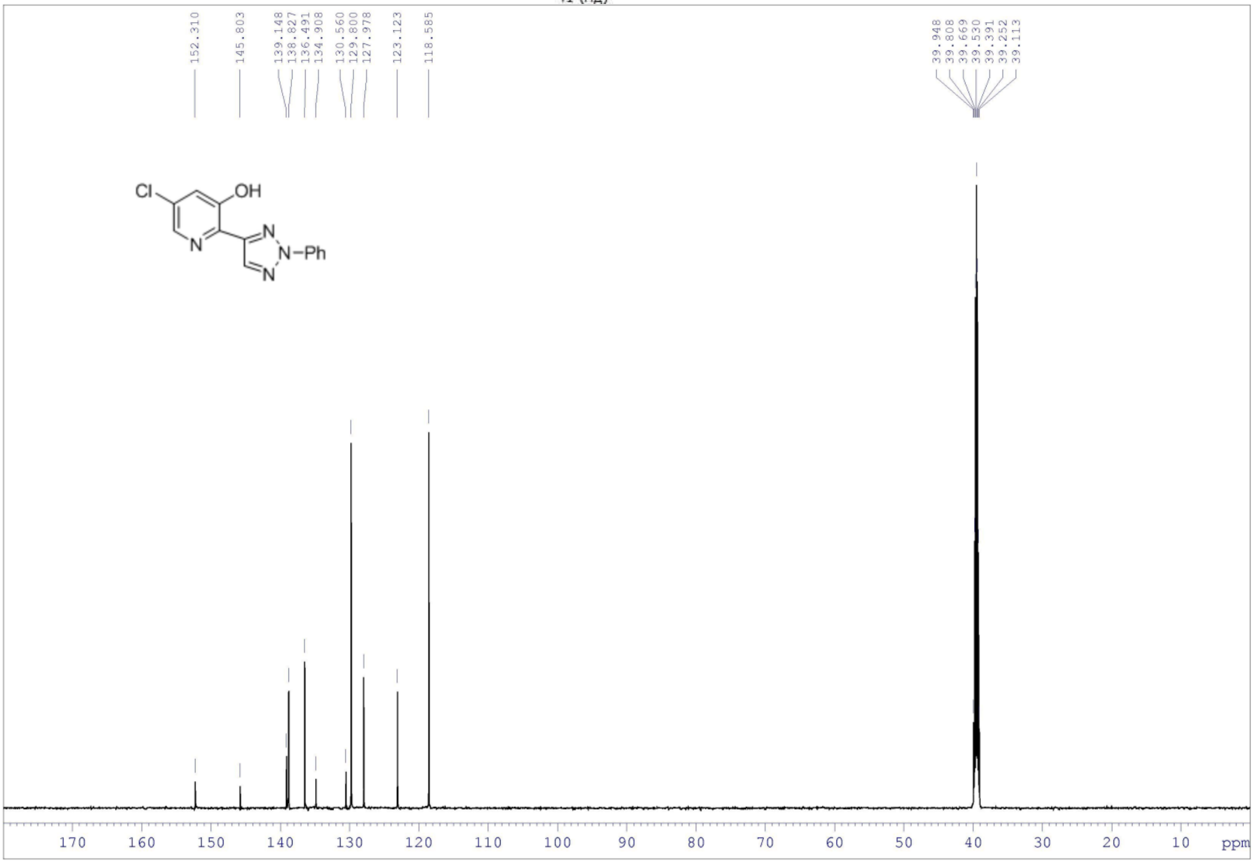

^{1}H and ^{13}C NMR spectra of compound **13g** in DMSO-d_6

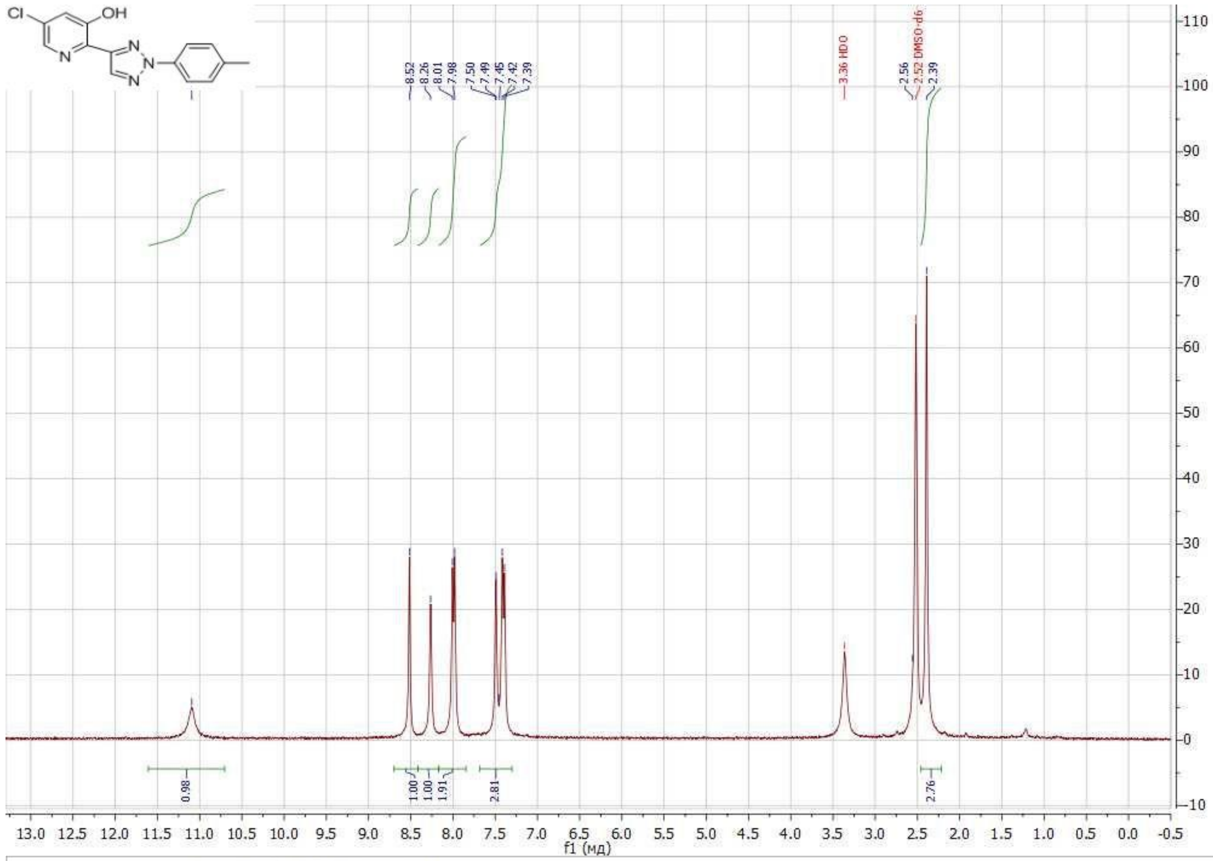

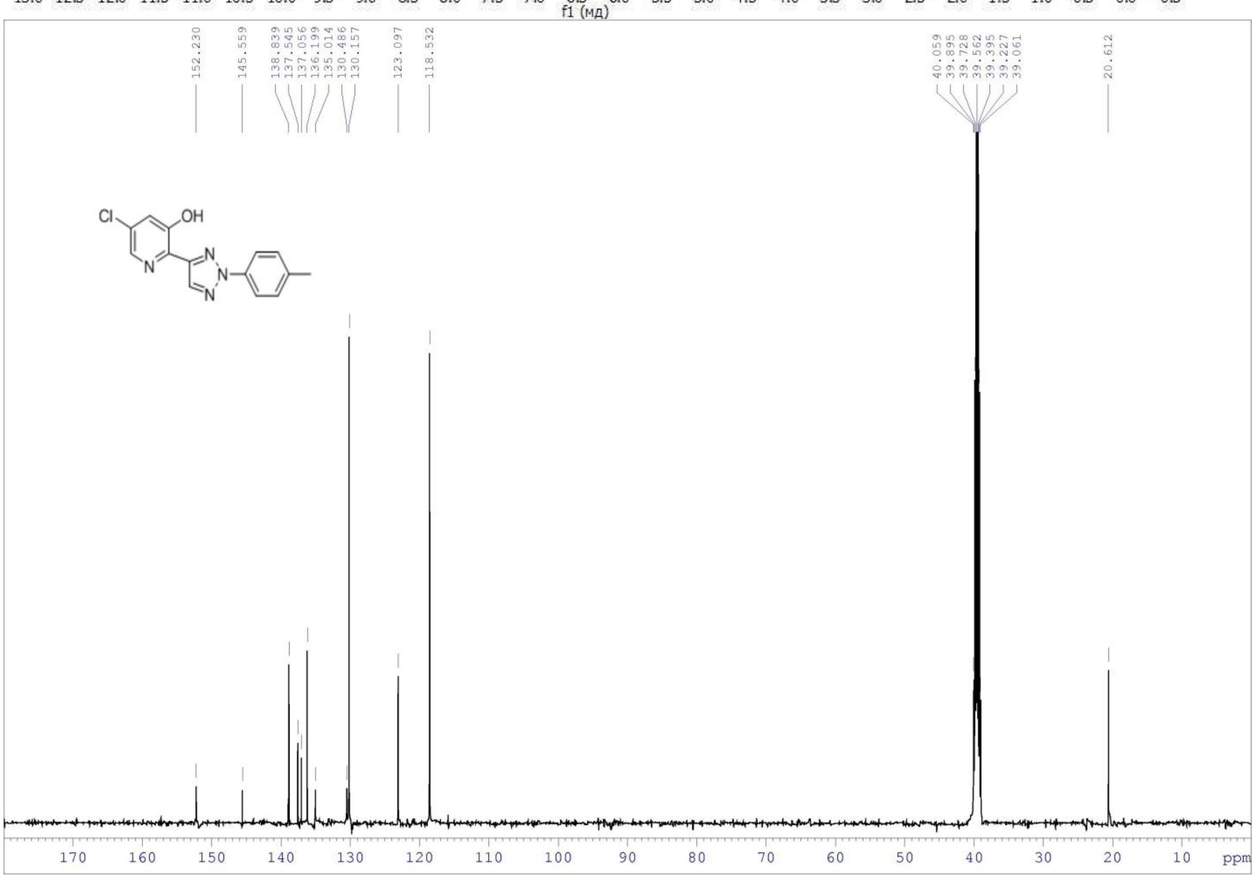

X-ray crystallographic data and refinement details.

X-ray diffraction data for **12c**, **13c**, and **13d** were collected at 100 K on a Rigaku XtaLAB Synergy-S diffractometer equipped with a HyPix6000HE area-detector (kappa geometry, shutterless ω-scan technique), using monochromatized CuK$_\alpha$ (**12c**, **13c**) or MoK$_\alpha$ (**13d**) radiation. The intensity data were integrated and analytically corrected for absorption and decay with the CrysAlisPro program [1]. The structures were solved by direct methods using SHELXT [2] and refined by the full-matrix least-squares minimization method on F^2 using SHELXL-2018 [3] in the OLEX2 program [4]. Positions of all atoms were found from the electron density-difference map. Atoms were refined with individual anisotropic (non-hydrogen atoms) or isotropic (hydrogen atoms) displacement parameters.

Crystal data and structure refinement are provided in Table S1. Bond distances and parameters of intermolecular hydrogen bonds are given in Tables S2–S7. Crystallographic data have been deposited at the Cambridge Crystallographic Data Centre. CCDC deposition numbers are 2333057 (**12c**), 2333058 (**13c**) and 2333059 (**13d**). More detailed crystallographic information can be retrieved free of charge via https://www.ccdc.cam.ac.uk/structures.

References

1. CrysAlisPro. Version 1.171.42. *Rigaku Oxford Diffraction*, **2023**.
2. Sheldrick, G. M. SHELXT - Integrated space-group and crystal-structure determination. *Acta Cryst*. **2015**, *A71*, 3-8. https://doi.org/10.1107/S2053273314026370
3. Sheldrick, G. M. Crystal structure refinement with SHELXL. *Acta Cryst*. **2015**, *C71*, 3-8. https://doi.org/10.1107/S2053229614024218
4. Dolomanov O.V.; Bourhis L.J.; Gildea R.J.; Howard J.A.K.; Puschmann H. OLEX2: a complete structure solution, refinement and analysis program. *J. Appl. Cryst.* **2009**, *42*, 339-341. https://doi.org/10.1107/S0021889808042726

Table S1. Crystal data and structure refinement for **12c**, **13c**, **13d**.

Identification code	**12c**	**13c**	**13d**
Empirical formula	$C_{14}H_9F_3N_4O$	$C_{14}H_9F_3N_4O$	$C_{14}H_8ClF_3N_4O$
Formula weight	306.25	306.25	340.69
Temperature, K	100.0(3)	100.0(2)	99.9(4)
Wavelength, Å	1.54184	1.54184	0.71073
Crystal system	Monoclinic	Monoclinic	Monoclinic
Space group	$P2_1/c$	$P2_1/n$	$P2_1/c$
Unit cell dimensions			
a, Å	8.44077(3)	5.22661(4)	15.49140(10)
b, Å	29.51699(11)	8.51464(7)	12.52173(8)
c, Å	10.45827(4)	29.0602(2)	7.12479(6)
β, °	93.4019(3)	90.8101(6)	103.2219(7)
Volume, $Å^3$	2601.045(17)	1293.128(17)	1345.424(17)
Z	8	4	4
Calculated density, $g \cdot cm^{-3}$	1.564	1.573	1.682
Absorption coefficient, mm^{-1}	1.155	1.161	0.330
F(000)	1248	624	688
Crystal size, mm	0.73 × 0.18 × 0.15	0.48 × 0.11 × 0.09	0.46 × 0.35 × 0.25
θ range for data collection, °	2.994-76.934	3.042-76.973	2.114-35.462
Index ranges	$-10 \leq h \leq 10$,	$-5 \leq h \leq 6$,	$-24 \leq h \leq 25$,
	$-37 \leq k \leq 37$,	$-10 \leq k \leq 10$,	$-20 \leq k \leq 20$,
	$-13 \leq l \leq 13$	$-36 \leq l \leq 35$	$-10 \leq l \leq 11$
Reflections			
Collected	76609	15742	128154
Independent [R_{int}]	5466 [0.0430]	2742 [0.0264]	5908 [0.0285]
Observed for $I>2\sigma(I)$	5391	2647	5768
Completeness to θ_{full} / θ_{max}	99.8 %	100.0 %	100.0 %
Max. / min. transmission	0.858 / 0.600	0.908 / 0.748	0.932 / 0.875
Data / restraints / parameters	5466 / 161 / 502	2742 / 88 / 254	5908 / 133 / 264
Goodness-of-fit on F^2	1.047	1.042	1.050
R1 / wR2 indices for $I>2\sigma(I)$	0.0332 / 0.0870	0.0339 / 0.0868	0.0274 / 0.0819
R1 / wR2 indices for all data	0.0335 / 0.0873	0.0348 / 0.0877	0.0279 / 0.0823
Extinction coefficient	0.00100(10)	0.0018(2)	-
$\Delta\rho(r)_{max}$ / $\Delta\rho(r)_{max}$, $\bar{e} \cdot Å^{-3}$	0.366 / -0.195	0.268 / -0.198	0.464 / -0.262
CCDC number	2333057	2333058	2333059

The structure of **12c**

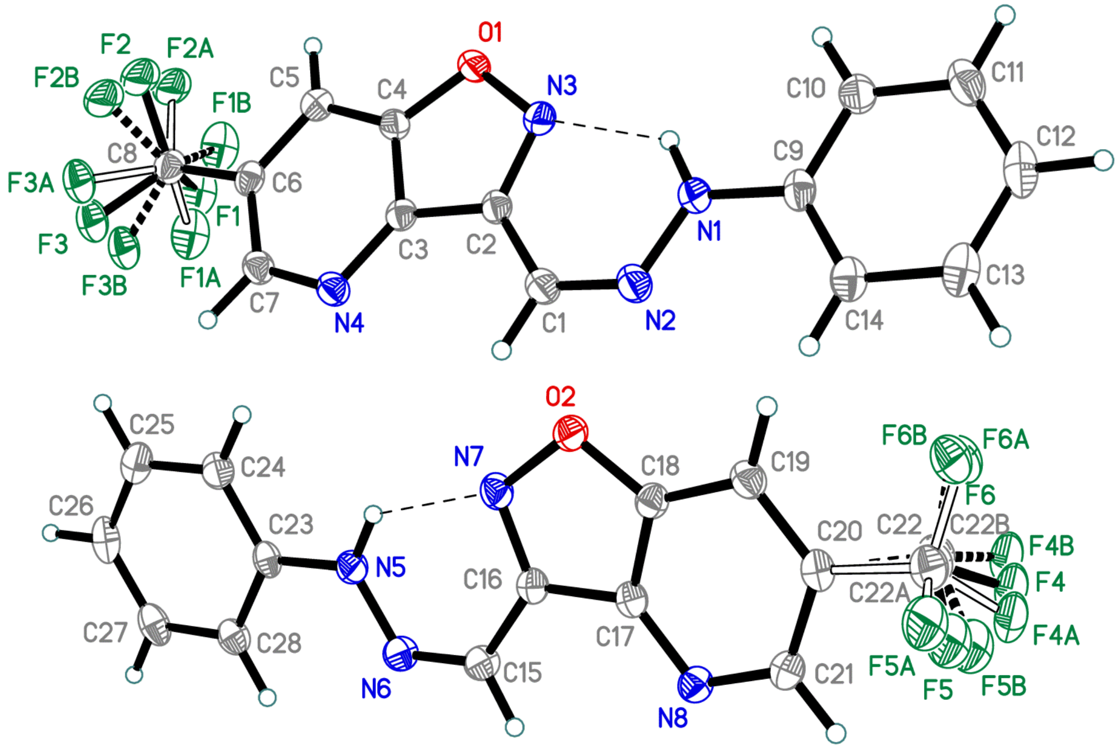

Figure S1: Two crystallographically non-equivalent molecules of **12c**. Fluorine atoms of the first molecule are disordered over three positions F1, F2, F3 / F1A, F2A, F3A / F1B, F2B, F3B with the disorder ratio of 0.980(2) : 0.0093(17) : 0.0111(16). The CF_3 group of the second molecule is also disordered over three positions C22, F4, F5, F6 / C22A, F4A, F5A, F6A / C22B, F4B, F5B, F6B with the disorder ratio of 0.566(3) : 0.247(3) : 0.187(3). Anisotropic displacement parameters are set to a 50% probability level.

Table S2: Bond lengths in **12c**, Å.

O1-N3	1.4222(12)	C8-F3B	1.341(3)	C19-C20	1.3838(16)
O1-C4	1.3530(13)	C9-C10	1.3968(16)	C20-C21	1.4075(16)
N1-H1N	0.908(16)	C9-C14	1.3932(16)	C20-C22	1.4972(19)
N1-N2	1.3408(13)	C10-H10	0.964(16)	C20-C22A	1.496(3)
N1-C9	1.4001(13)	C10-C11	1.3875(16)	C20-C22B	1.496(3)
N2-C1	1.2963(15)	C11-H11	0.969(16)	C21-H21	0.976(15)
N3-C2	1.3136(14)	C11-C12	1.3890(19)	C22-F4	1.349(2)
N4-C3	1.3415(14)	C12-H12	0.984(16)	C22-F5	1.349(2)
N4-C7	1.3294(14)	C12-C13	1.3870(19)	C22-F6	1.346(2)
C1-H1	0.967(15)	C13-H13	0.982(16)	C22A-F4A	1.347(2)
C1-C2	1.4500(15)	C13-C14	1.3928(16)	C22A-F5A	1.347(3)
C2-C3	1.4456(14)	C14-H14	0.932(16)	C22A-F6A	1.346(2)
C3-C4	1.3822(15)	O2-N7	1.4337(11)	C22B-F4B	1.347(3)
C4-C5	1.3836(15)	O2-C18	1.3534(13)	C22B-F5B	1.347(3)
C5-H5	0.954(15)	N5-H5N	0.897(17)	C22B-F6B	1.346(3)
C5-C6	1.3847(16)	N5-N6	1.3371(13)	C23-C24	1.3945(16)
C6-C7	1.4086(15)	N5-C23	1.3980(13)	C23-C28	1.3957(15)
C6-C8	1.5001(15)	N6-C15	1.2953(14)	C24-H24	0.961(16)
C7-H7	0.995(14)	N7-C16	1.3149(14)	C24-C25	1.3852(16)
C8-F1	1.3423(12)	N8-C17	1.3429(14)	C25-H25	0.959(16)
C8-F2	1.3408(12)	N8-C21	1.3272(14)	C25-C26	1.3885(18)
C8-F3	1.3402(12)	C15-H15	0.956(15)	C26-H26	0.998(16)
C8-F1A	1.341(3)	C15-C16	1.4494(15)	C26-C27	1.3883(19)
C8-F2A	1.341(3)	C16-C17	1.4419(14)	C27-H27	0.938(16)
C8-F3A	1.341(3)	C17-C18	1.3813(15)	C27-C28	1.3869(16)
C8-F1B	1.341(3)	C18-C19	1.3878(15)	C28-H28	0.973(15)
C8-F2B	1.341(3)	C19-H19	0.973(15)		

Table S3: Hydrogen bond parameters for **12c**, Å and °.

D-H...A	d(D-H)	d(H...A)	d(D...A)	<(DHA)
N1-H1N...N3	0.908(16)	2.005(16)	2.7095(13)	133.2(13)
N5-H5N...N7	0.897(17)	2.041(16)	2.7373(13)	133.5(14)

The structure of **13c**

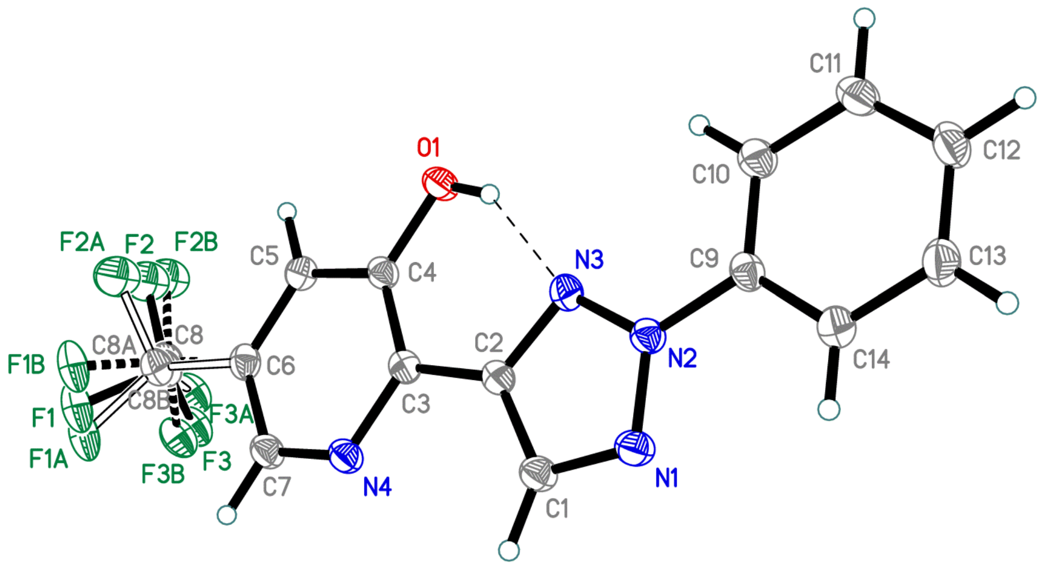

Figure S2: Two crystallographically non-equivalent molecules of **13c**. The CF_3 group is disordered over three positions C8, F1, F2, F3 / C8A, F1A, F2A, F3A / C8B, F1B, F2B, F3B with the disorder ratio of 0.722(3) : 0.142(3) : 0.136(3). Anisotropic displacement parameters are set to a 50% probability level.

Table S4: Bond lengths in **13c**, Å.

N1-N2	1.3415(13)	C5-C6	1.3826(16)	C8B-F2B	1.342(3)
N1-C1	1.3280(15)	C6-C7	1.3934(17)	C8B-F3B	1.343(3)
N2-N3	1.3348(13)	C6-C8	1.4999(17)	C9-C10	1.3885(16)
N2-C9	1.4292(14)	C6-C8A	1.498(3)	C9-C14	1.3913(16)
N3-C2	1.3414(14)	C6-C8B	1.498(3)	C10-H10	0.959(16)
C1-H1	0.970(15)	C7-H7	0.977(15)	C10-C11	1.3913(16)
C1-C2	1.4012(16)	C7-N4	1.3281(15)	C11-H11	0.963(17)
C2-C3	1.4628(15)	C8-F1	1.3455(19)	C11-C12	1.3868(18)
C3-C4	1.4013(16)	C8-F2	1.3401(19)	C12-H12	0.960(16)
C3-N4	1.3496(14)	C8-F3	1.3496(17)	C12-C13	1.3864(18)
C4-O1	1.3557(13)	C8A-F1A	1.342(3)	C13-H13	0.946(17)
C4-C5	1.3936(15)	C8A-F2A	1.340(3)	C13-C14	1.3907(16)
O1-H1A	0.847(17)	C8A-F3A	1.342(3)	C14-H14	0.952(16)
C5-H5	0.988(15)	C8B-F1B	1.342(3)		

Table S5: Hydrogen bond parameters for **13c**, Å and °.

D-H...A	d(D-H)	d(H...A)	d(D...A)	<(DHA)
O1-H1A...N3	0.847(17)	1.944(17)	2.7096(13)	149.8(14)

The structure of **13d**

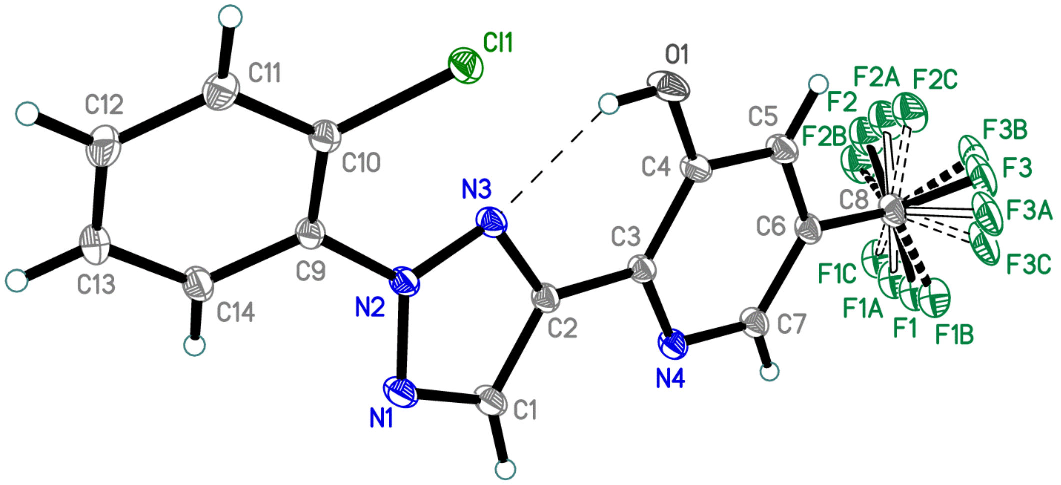

Figure S3: Two crystallographically non-equivalent molecules of **13d**. The CF_3 group is disordered over four positions F1, F2, F3 / F1A, F2A, F3A / F1B, F2B, F3B / F1C, F2C, F3C with the disorder ratio of 0.367(2) : 0.296(2) : 0.258(3) : 0.079(2). Anisotropic displacement parameters are set to a 50% probability level.

Table S6: Bond lengths in **13d**, Å.

Cl1-C10	1.7272(7)	C5-H5	0.930(13)	C8-F3B	1.342(2)
N1-N2	1.3413(8)	C5-C6	1.3888(10)	C8-F1C	1.344(2)
N1-C1	1.3313(9)	C6-C7	1.3938(9)	C8-F2C	1.344(2)
N2-N3	1.3335(8)	C6-C8	1.4952(10)	C8-F3C	1.348(2)
N2-C9	1.4228(8)	C7-H7	0.919(13)	C9-C10	1.3998(9)
N3-C2	1.3398(8)	C7-N4	1.3359(9)	C9-C14	1.3975(9)
C1-H1	0.955(14)	C8-F1	1.3391(14)	C10-C11	1.3931(9)
C1-C2	1.4034(9)	C8-F2	1.3644(15)	C11-H11	0.972(14)
O1-H1A	0.833(17)	C8-F3	1.3436(15)	C11-C12	1.3892(10)
O1-C4	1.3504(8)	C8-F1A	1.3415(17)	C12-H12	0.974(14)
C2-C3	1.4602(9)	C8-F2A	1.3448(17)	C12-C13	1.3913(11)
C3-C4	1.4095(9)	C8-F3A	1.3475(17)	C13-H13	0.964(14)
C3-N4	1.3433(8)	C8-F1B	1.3533(19)	C13-C14	1.3874(10)
C4-C5	1.3922(9)	C8-F2B	1.3460(19)	C14-H14	0.963(13)

Table S7: Hydrogen bond parameters for **13d**, Å and °.

D-H...A	d(D-H)	d(H...A)	d(D...A)	<(DHA)
O1-H1A...N3	0.833(17)	1.971(17)	2.6981(8)	145.3(16)
